# An episomal DNA vector platform for the persistent genetic modification of pluripotent stem cells and their differentiated progeny

**DOI:** 10.1016/j.stemcr.2021.11.011

**Published:** 2021-12-22

**Authors:** Alicia Roig-Merino, Manuela Urban, Matthias Bozza, Julia D. Peterson, Louise Bullen, Marleen Büchler-Schäff, Sina Stäble, Franciscus van der Hoeven, Karin Müller-Decker, Tristan R. McKay, Michael D. Milsom, Richard P. Harbottle

**Affiliations:** 1 DNA Vectors, German Cancer Research Center (DKFZ), Heidelberg 69120, Germany; 2 Stem Cell Biology, Manchester Metropolitan University (MMU), Manchester M1 5GD, UK; 3 Heidelberg Institute for Stem Cell Technology and Experimental Medicine (Hi-STEM), Heidelberg 69120, Germany; 4 Division of Experimental Hematology, DKFZ, Heidelberg 69120, Germany; 5 Translational Cancer Epigenomics, Division of Translational Medical Oncology, DKFZ, Heidelberg 69120, Germany; 6 Transgenics Service, DKFZ, Heidelberg 69120, Germany; 7 Tumor Models, DKFZ, Heidelberg 69120, Germany

**Keywords:** embryonic stem cell, induced pluripotent stem cell, DNA vector, episome, nonintegrative, SMAR, self-renewal, transgenesis, differentiation, reprogramming

## Abstract

The genetic modification of stem cells (SCs) is typically achieved using integrating vectors, whose potential integrative genotoxicity and propensity for epigenetic silencing during differentiation limit their application. The genetic modification of cells should provide sustainable levels of transgene expression, without compromising the viability of a cell or its progeny. We developed nonviral, nonintegrating, and autonomously replicating minimally sized DNA nanovectors to persistently genetically modify SCs and their differentiated progeny without causing any molecular or genetic damage. These DNA vectors are capable of efficiently modifying murine and human pluripotent SCs with minimal impact and without differentiation-mediated transgene silencing or vector loss. We demonstrate that these vectors remain episomal and provide robust and sustained transgene expression during self-renewal and targeted differentiation of SCs both *in vitro* and *in vivo* through embryogenesis and differentiation into adult tissues, without damaging their phenotypic characteristics.

## Highlights

• Nanovectors are used to engineer SCs efficiently, safely, and persistently
• Isogenic SC lines retain their capacity for self-renewal and pluripotency
• Nanovectors survive reprogramming and differentiation without loss or silencing
• Nanovectors are a universal genetic tool for the modification of any cell

## Graphical abstract

**Figure:**
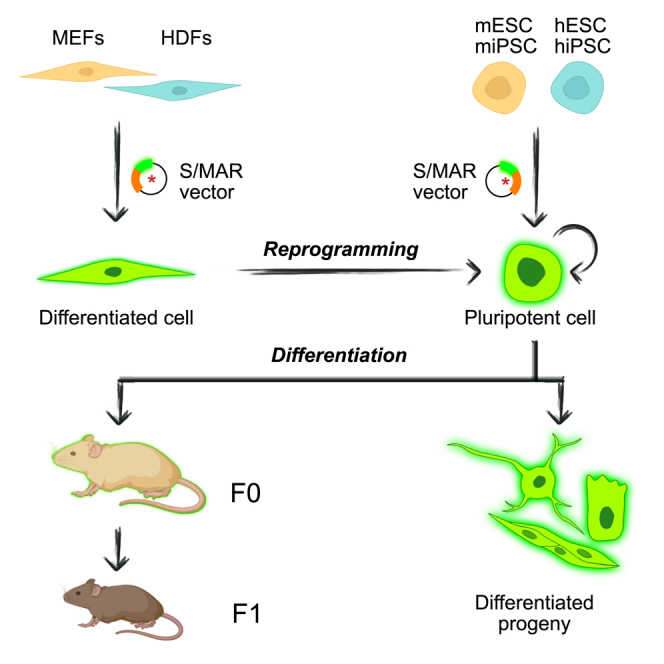

## Teaser

In this study, Harbottle and colleagues show that nonviral, nonintegrating, and autonomously replicating minimally sized DNA nanovectors can be used to persistently modify SCs genetically without causing any molecular or genetic damage. The DNA vectors remain episomal during self-renewal and differentiation *in vitro* and *in vivo*, while providing robust and sustained transgene expression.

## Introduction

Pluripotent stem cells (PSCs) are an invaluable source of cells for regenerative therapies due to their capacity for proliferation, self-renewal, and their potential for multi-lineage differentiation (He et al., 2009; Schwanke et al., 2014). Induced PSCs (iPSCs) can be derived from somatic cells (Takahashi and Yamanaka, 2006) and isolated using minimally invasive techniques. This not only limits concerns regarding the use of embryonic SCs (ESCs) but the risk of immune rejection as an autologous therapy. Therefore, iPSCs are an attractive tool for personalized medicine, drug screening, and the generation of disease models (Takahashi and Yamanaka, 2013).

SCs are notoriously difficult to modify genetically; they are typically refractory to transfection, their extensive proliferation leads to vector dilution, and the dramatic changes in the cellular milieu following differentiation can lead to transgene silencing.

A variety of methods are used to persistently genetically modify and derive SCs (Table 1), but most rely on integrating lentiviral vectors. Despite advances, low transduction efficiency and silencing are still observed using retroviruses in hematopoietic (HSCs) and mesenchymal SCs (MSCs) (Zhang et al., 2002). Additionally, problems associated with random insertion into untranscribed regulatory regions (5′UTR) and consequent dysregulation of neighboring genes (Cattoglio et al., 2007) (Kotterman et al., 2015) affect the use of lentiviruses in SCs (Herbst et al., 2012).

**Table 1:** Overview of gene therapy vectors

	γ-retrovirus	EBNA vectors	Transposons	Minicircles	SMAR minicircles	SMAR plasmids (pSMAR)	SMAR nanovectors (nSMAR)
**Capacity**	Medium	High	High	High	High	High	High
**Maintenance**	Yes	Yes	Yes	No	Yes	Yes	Yes
**Integrative**	Yes	Possibly	Yes	No	No	No	No
**Replicative**	Yes	Yes	Yes	No	Yes	Yes	Yes
**Oncogenic**	High	High	Medium	Low	Low	Low	Low
**Manufacturing**	Difficult	Easy	Easy	Difficult	Difficult	Easy	Easy
**Immunogenic**	High	High	Medium	Medium	Medium	Medium	Low
**Bacterial antibiotic free**	No	No	Yes	Yes	Yes	No	Yes

Vectors such as transposons can be used to genetically engineer PSCs (Park et al., 2018; Querques et al., 2019), while sustaining transgene expression during differentiation (Chen et al., 2009; Orbán et al., 2009; Wilber et al., 2007). However, they can randomly integrate, potentially interfering with the cells' integrity. They can also be engineered using sequence-specific nucleases (Czerwińska et al., 2019; Song and Ramakrishna, 2018). However, despite intensive research, undesired off-target effects and editing efficiency remain an issue requiring thorough screening and genomic characterization (Kim et al., 2017). SC engineering and iPSC derivation can also be achieved using episomal plasmids, which predominantly comprise viral components such as Epstein-Barr virus Nuclear Antigen 1 *(EBNA-1)* (Sugden et al., 1985; Thyagarajan et al., 2009; Yates et al., 1985) or the large T antigen from Simian Virus 40 *(SV40)*. EBNA-based systems rely on the oncoprotein EBNA-1 (Humme et al., 2003), which interacts with the *MYC* promoter (Canaan et al., 2009; Sung and Pagano, 1995), and can dysregulate genes associated with cell growth, rendering them potentially oncogenic (Canaan et al., 2009). Thus, using an episomal vector devoid of viral elements for its maintenance is highly desirable.

The plasmid pEPI can function as an episome using genetic elements known as scaffold/matrix attachment regions (SMARs) (Piechaczek et al., 1999). SMARs interact with transcription factories, influencing gene expression by controlling the recruitment of transcription factors, chromatin structure, and accessibility (Hagedorn et al., 2013). In a plasmid, SMARs facilitate episomal replication and maintenance (Stehle et al., 2003) in various cells (Hagedorn et al., 2012), including human HSC (Papapetrou et al., 2006), and prevent epigenetic silencing, while enhancing transgene expression (Piechaczek et al., 1999). Upon delivery, vector molecules reach the nucleus and are stochastically established depending on their proximity to nuclear compartments (Hagedorn et al., 2017; Stehle et al., 2007). Vectors are episomally maintained at low copy numbers (Stehle et al., 2007), are stable in the absence of selection (Piechaczek et al., 1999), are co-segregated with chromosomes during mitosis, and have unlimited cloning capacity (Lufino et al., 2007).

SMAR vectors have been systematically modified to improve their application (Hagedorn and Lipps, 2013) by swapping the original promoter for *in vivo* applications (Manzini et al., 2010; Wong et al., 2011; Argyros et al., 2008), by reducing potential immunogenicity, by reducing or removing the vector backbone's CpG content (Haase et al., 2010), or by generating SMAR minicircles (Argyros et al., 2011). Minicircle production is inefficient, difficult, and costly, resulting in heterogeneous DNA. SMAR nanovectors based on an RNA-Out technology (Luke et al., 2009) are produced more simply with higher purity.

Here, we describe a nonviral, nonintegrating, and autonomously replicating SMAR vectors that can be used to persistently engineer SCs without causing molecular or genetic damage, while providing sustained transgene expression during differentiation and reprogramming. Within this study, we generated two novel vectors, pSMAR and nSMAR, by refining their composition and functional elements. We compared our new vectors' behavior to the original pEPI vector and evaluated their episomal replication, establishment efficiency, long-term maintenance, and transgene expression. Both newly designed vectors outperform the originals by every measure.

## Results

### pSMAR and nSMAR generate highly expressing stable SC lines while remaining episomal

**Figure 1:**
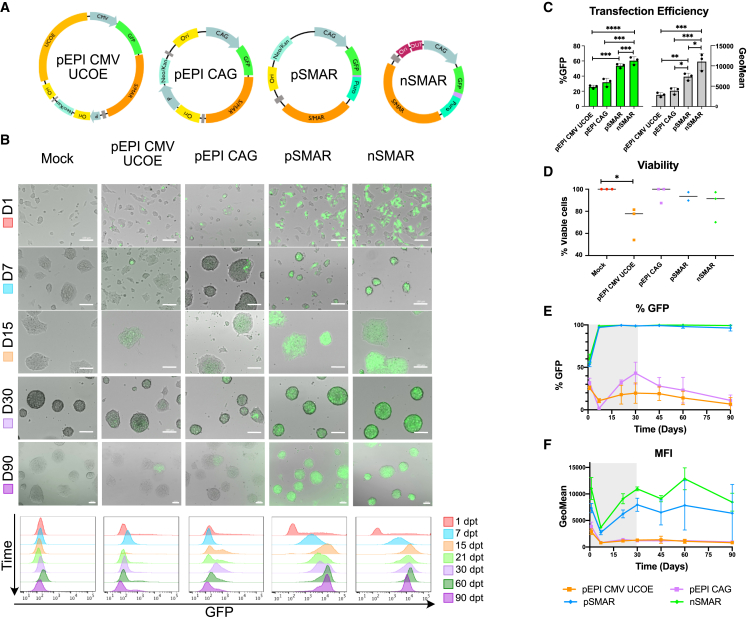
Increased vector performance is reflected by improved vector design (A) Schematics of DNA vectors used in this study. (B) Monitoring and quantification of GFP expression in mESC by microscopy and FACS analysis. GFP fluorescence gated on the alive population. Images and histograms from three (n = 3) independent experiments (scale bars = 100 μm). (C) Transfection efficiency (24 hpt) of transfected mESC. Results expressed as mean ± SD of %GFP + cells and MFI (GeoMean) from three (n = 3) independent experiments. Statistical analysis was performed as follows: GFP and MFI: Shapiro-Wilk normality test passed, 1-way ANOVA with Tukey's multiple comparison test. GFP: pEPI-CMV-UCOE versus pEPI-CAG, p-val = ns; pEPI-CMV-UCOE versus pSMAR, ^∗∗∗^p = 00.002; pEPI-CMV-UCOE versus nSMAR, ^∗∗∗∗^p < 00.001; pEPI-CAG versus pSMAR, ^∗∗∗^p = 00.009; pEPI-CAG versus nSMAR, ^∗∗∗^p = 00.001; pSMAR versus nSMAR, p = ns). MFI: pEPI-CMV-UCOE versus pEPI-CAG, p = ns; pEPI-CMV-UCOE versus pSMAR, ^∗∗^p = 00.094; pEPI-CMV-UCOE versus nSMAR, ^∗∗∗^p = 00.002; pEPI-CAG versus pSMAR, ^∗^p = 00.361; pEPI-CAG versus nSMAR, ^∗∗∗^p = 00.005; pSMAR versus nSMAR, ^∗^p = 00.255). (D) Cell viability of transfected mESC at 24 hpt from three (n = 3) independent experiments. The results are expressed as % alive transfected cells normalized to alive mock cells transfected without DNA. Statistical analysis was performed using Shapiro-Wilk normality test and 1-way ANOVA with Dunnett's multiple comparison test (^∗^p = 0.0258). (E) Plot showing %GFP + cells over 3 months. The grayed-out area corresponds to the antibiotic selection period. The results are expressed as mean ± SD from three (n = 3) independent experiments. (F) Plot showing the MFI (GeoMean) variation over time. The grayed-out area corresponds to the antibiotic selection period. The results are expressed as mean ± SD from three (n = 3) independent experiments.

Refined SMAR vectors are based on pEPI-CMV-UCOE (Hagedorn et al., 2013) (Figure 1A). The SMAR element was retained and the CMV promoter replaced with the CAG (Fregien and Davidson, 1986; Miyazaki et al., 1989) to provide robust transgene expression (pEPI-CAG), their composition reorganized by directly coupling the selection marker to the expression cassette and SMAR motif (pSMAR) (Bozza et al., 2020). We generated minimally sized nanovectors (nSMAR) by eliminating bacterial sequences and reducing the backbone to 431 bp, a reduction of 17.41%. Each vector encoded the reporter gene *GFP* (Figure 1B) and was directly compared to determine efficiency, stability, and durability of expression.

Electroporation of mouse ESCs (mESCs) with pEPI-CMV-UCOE resulted in transfection efficiency of 25.8% ± 2.2% compared with slightly increased efficiency of 31.8% ± 5.5% with pEPI-CAG. Transfection efficiency and fluorescence intensity dramatically increased when using pSMAR (53.6% ± 2.8%) and nSMAR (60.4% ± 5.2%) compared with pEPI vectors (Figure 1C). pEPI-CMV-UCOE resulted in the lowest transfection efficiency and reduced cell viability (71% ± 15%) (Figure 1D).

The vectors' ability to form stable cell lines was evaluated by following their expression for 30 days under selection and monitoring transgene maintenance >60 days in its absence. After 7 days, we observed a decrease in GFP + cells in pEPI-CMV-UCOE (10.7% ± 2.4%) and pEPI-CAG (1.8% ± 0.5%). However, GFP-neomycin-resistant colonies grew further. In contrast, pSMAR and nSMAR provided robust and stable transgene expression and GFP + cells could be observed throughout the experiment, even after >60 days with no selection (Figures 1E and 1F). Additionally, we validated the functionality of pSMAR and nSMAR in primary cells, such as murine embryonic fibroblasts (MEFs) (Figure S1A), murine iPSCs (miPSCs) (Figure S1B), and human iPSCs (hiPSCs) (Figures S1C and S1D). pEPI vectors could not generate stable GFP-hiPSCs (Figure S1C), and neomycin-resistant GFP clones remained growing. We obtained stable and highly expressing GFP-hiPSCs with pSMAR (98.5%) and nSMAR (99.6%), even after 3 months with no selection (Figure S1D). For translational applications, we generated pSMAR and nSMAR expressing hiPSC lines derived from urine iPSCs (UiPSCs) in the absence of selection. We observed stable and persistent GFP expression >170 days (Figure S1E).

Differences in vector performance were due to the vectors' composition not DNA purity (Figure S1F and S1G). We evaluated their integrity and stability by Southern blot from stably transfected mESCs, in which we observed two unique bands of 7,162 bp (pSMAR) and 5,915 bp (nSMAR) (Figure S1H). To further confirm the vectors' episomal status, we performed plasmid rescue, in which circular episomal forms could be retrieved in 90% of the cases (Figure S1I). We confirmed the rescued vector's integrity and sequence by restriction digestion and PCR amplification for both transgene and SMAR motif.

pSMAR and nSMAR vectors outperformed pEPI vectors in delivering high levels of stable transgene expression in rapidly proliferating cells while remaining episomal. Given the poor performance and rapid loss of transgene expression of pEPI-transfected cells, further experiments were only performed using pSMAR and nSMAR.

### SMAR vectors show minimal impact on hESCs

**Figure 2:**
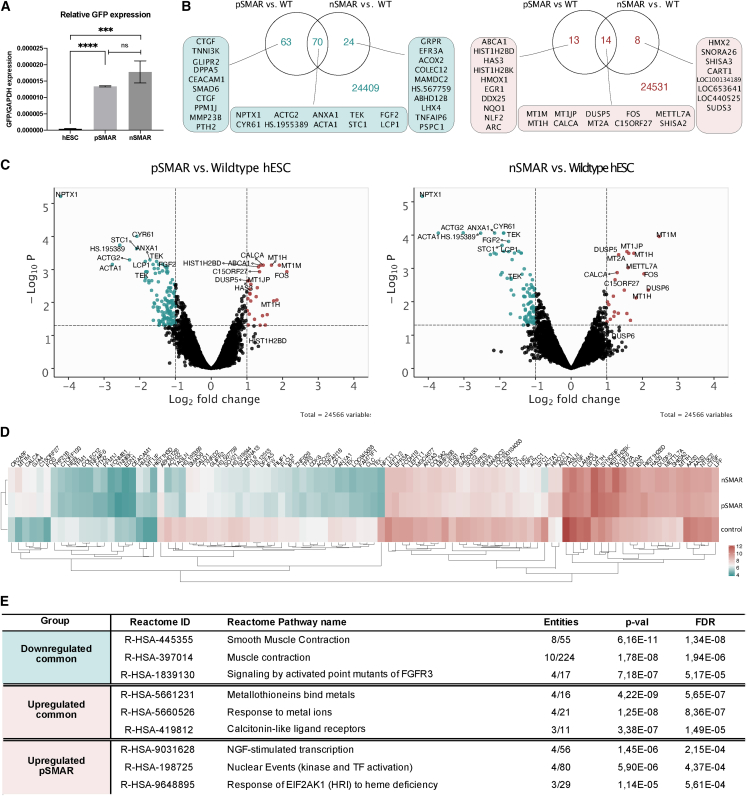
SMAR nanovectors have minimal impact on cells' transcriptome hESCs were electroporated with pSMAR and nSMAR. RNA was DNaseI-treated before microarray analysis (IlluminaHuman12 chip). RNA extractions from three different cell samples (n = 3) were used. Expression profiles were background corrected, quantile normalized, and log_2_ transformed using the Limma package from R. Linear modeling was performed, and the empirical Bayes method was used to assess differential expression. (A) Comparison of transgene expression in transfected hESC. qPCR analysis of GFP normalized to GAPDH. One-way ANOVA (p < 0.0001) and unpaired 2-tailed T test (^∗∗∗∗^p < 0.0001, ^∗∗∗^p = 0.0009, ns = 0.0870). (B) Venn Diagrams indicate the number of unique or similarly dysregulated genes between each pairwise comparison with adjusted p < 0.05 and FC > 2. The top ten differentially expressed genes within each category are listed. (C) Volcano plots display pairwise comparisons of expression profiles from pSMAR-hESC and nSMAR-hESCs versus wild-type cells. Adjusted p < 0.05 (-log_10_P of 1.3) and an FC > 2 (log_2_FC of 1). Green = downregulated and red = upregulated genes. The top ten differentially expressed genes are listed. (D) Hierarchical clustering was performed using the average normalized expression values from the top 100 differentially expressed genes using Euclidean as a distancing measure and median as a clustering method for each group (n = 3). (E) Reactome analysis was performed on the list of common or vector-specific dysregulated genes.

To investigate the impact of SMAR vectors on SCs, hESC engineered with either pSMAR or nSMAR were subjected to microarray analysis. Their transcriptional profiles were compared with those of untransfected cells. We observed 160 and 116 differentially expressed genes, respectively. Sixty-three downregulated genes are unique to pSMAR modification, while only 24 are unique to nSMAR modification; 13 upregulated genes are unique to pSMAR modification, while only eight are unique to nSMAR modification (Figures 2B and 2C).

GFP levels were similar in pSMAR- and nSMAR-modified hESCs, suggesting that the differences found between the transcriptional profiles of the modified hESC are not attributed to the intensity of transgene expression (Figure 2A). Subsequent gene cluster analysis of the top 100 dysregulated genes indicates a closer relationship between pSMAR and nSMAR, while nonmodified hESCs have the furthest relationship from both (Figure 2D). We performed Reactome and GO TERM analysis on the unique and common dysregulated genes. Common downregulated genes are involved with muscle contraction and FGF3 signaling, while commonly upregulated genes are associated with metallothioneins and response to metal ions. Upregulated pSMAR-specific genes belong to NGF-stimulated transcription, kinase and transcription factor activation, and response to heme deficiency. We were unable to find statistically significant enriched gene sets for nSMAR-specific genes (Figure 2E). For detailed information refer to Figure S2 and Table S1.

These results suggest that SMAR vectors have a minimal impact on the host cell's endogenous transcription, and nSMAR causes the least disturbance to cells' molecular integrity, resulting in no significant dysregulation.

### SMAR vectors can genetically modify murine and human primary fibroblasts and persist during reprogramming

**Figure 3:**
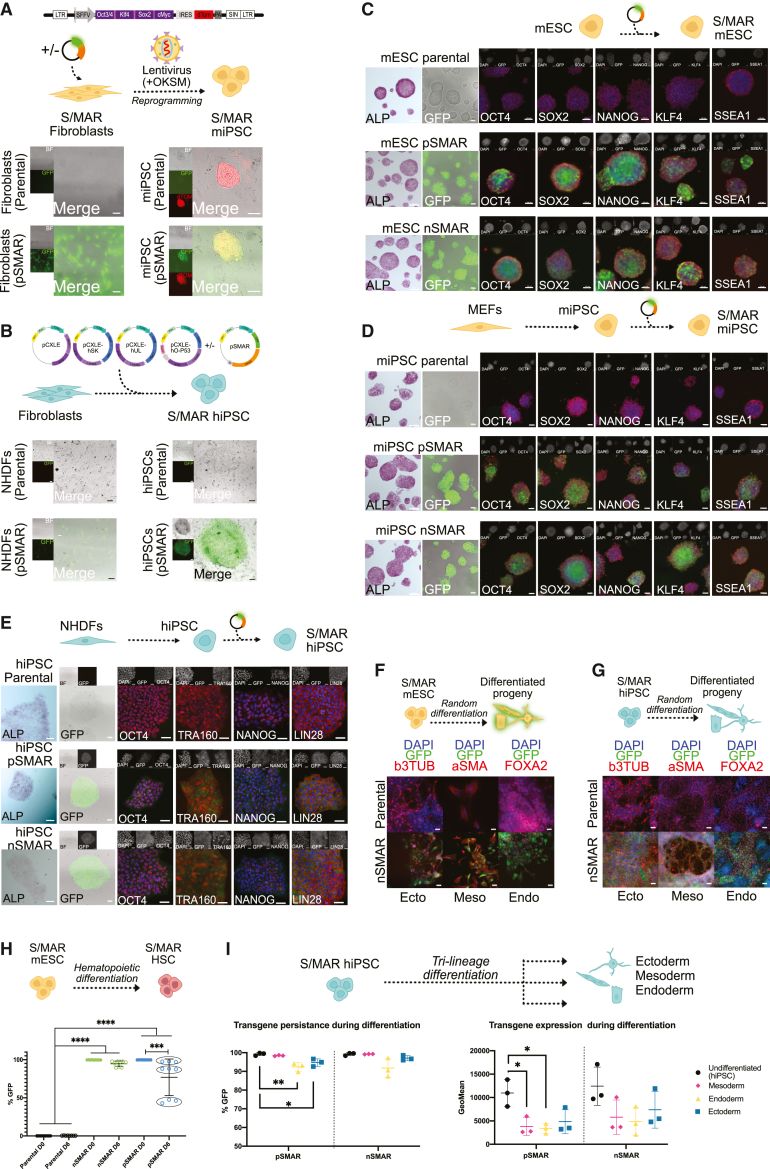
Maintenance of transgene expression through reprogramming and differentiation in miPSCs and hiPSCs (A) Genetic modification of MEFs with pSMAR (GFP) and further reprogramming to miPSCs upon transduction with pWPI 4-in-1 lentiviral particles, expressing the reprogramming factors OKSM and dTOM (scale bars = 100 μm). (B) Simultaneous labeling with pSMAR (GFP) and reprogramming of hiPSCs using EBNA-1 episomal vectors (scale bars = 100 μm). (C) Immunofluorescence (IF) of pluripotency markers of parental, pSMAR, and nSMAR stable mESCs. Expression and localization of OCT4, NANOG, SSEA-1, Alkaline Phosphatase, and endogenous GFP (scale bars = 100 μm). (D) IF of pluripotency markers of miPSCs generated from CF1-MEFs, genetically modified at the SC stage (scale bars = 100 μm). (E) IF staining of hiPSC modified at the SC stage. Pluripotency markers (OCT4, LIN28, NANOG, TRA-160) and endogenous GFP (scale bars = 100 μm). (F) IF staining of Mesoderm (αSMA), Ectoderm (β3TUB), and Endonderm (FOXA2) in randomly differentiated mESCs. Endogenous GFP was preserved (scale bars = 100 μm). (G) IF staining of guided three-germ layer differentiation of parental and stable modified hiPSCs (scale bars = 100 μm). (H) Hematopoietic differentiation of parental (passage 14, n = 3), pSMAR (passage 20, 14, 5; n = 3), and nSMAR mESC clones (passage 20, 14, 5; n = 3). The plot represents pooled biological replicates for the same vector. The GFP expression of each clone was analyzed using flow cytometry before (mESCs, day 0) and after (HSC, day 6) differentiation. The circles represent three technical replicates (n = 3) of the same clone. One-way ANOVA and Tukey's multiple comparison test were used for statistical analysis (^∗∗∗∗^p < 00.001; ^∗∗∗^p = 00.002). (I) Quantification of transgene expression and persistence during differentiation. hiPSC modified with pSMAR (n = 3) or nSMAR (n = 3) were differentiated into Ectoderm, Mesoderm, or Endoderm. Transgene expression was measured by FACS in the differentiated lineages compared with their respective undifferentiated control (hiPSC). Two-tailed unpaired T tests were used for statistical analysis (^∗∗^p = 00.081, ^∗^p = 0.021, ^∗^p = 0.022, ^∗^p = 0.012).

We then evaluated the SMAR vectors' suitability, performance, and survival during reprogramming to iPSCs. Mouse lung fibroblasts were modified with pSMAR and selected to generate stable GFP-fibroblasts (Figure 3A). The episomal state of pSMAR was confirmed by plasmid rescue (Figure S3A). Then, pSMAR-fibroblasts were reprogrammed using pWPI-4in1, encoding the reprogramming factors *OKSM* and *dTOMATO* (Maetzig et al., 2014; Warlich et al., 2011). After 14 days, morphologically distinct dome-shaped colonies emerged, expressing both GFP and dTOM, indicating the presence of SMAR vectors and the reprogramming lentivirus, respectively. GFP expression could be observed during reprogramming, proving the vectors' resistance to epigenetic silencing (Figure 3A).

In a translational approach, we validated the persistence of transgene expression in hiPSCs derived from dermal fibroblasts following co-transfection of pSMAR with the well-established EBNA-1/OriP reprogramming system (Okita et al., 2011). Morphologically distinct GFP-hiPSC colonies were obtained, indicating the presence and survival of pSMAR during reprogramming (Figure 3B).

### SMAR vectors genetically modify murine and human cells without impairing pluripotency

We assessed if genetic modification of PSCs with SMAR vectors impacts pluripotency measuring the effect of SMAR vectors on the functionality and pluripotency of transfected mESCs using pSMAR and nSMAR. The cell lines were alkaline phosphatase (ALP)-positive and expressed all pluripotency markers in immunofluorescence (IF) stainings and Western blots (Figure S3B) and expression of GFP (Figure 3C).

To address the genetic modification of miPSCs, MEFs were reprogrammed using a pWPI-4in1 lentivirus, as described above, and miPSCs were electroporated with pSMAR and nSMAR (refer to Figure S1B). Pluripotency of modified miPSCs was confirmed via ALP and IF stainings for pluripotency markers (Figure 3D) and Western blot (Figure S3B).

Similarly, we modified hiPSCs derived from NHDFs with pSMAR and nSMAR. The pluripotency of the engineered hiPSCs remained intact, as cells were positive for all pluripotency markers tested (Figures 3E and S3C). We observe no differences in expression when we compared the pluripotency factors OCT4 and NANOG in similarly expressing pSMAR or nSMAR-hiPSCs and the parental hiPSC line (Figure S3D).

### SMAR vectors survive *in vitro* random differentiation

Engineered mESCs were subjected to random differentiation; EBs were monitored and imaged regularly to check for GFP expression and hence the presence and function of the vectors during differentiation (Figure S3F). Independent experiments showed that stable pSMAR and nSMAR mESCs formed compact EBs and differentiated into structures such as neurons or contracting myocytes. Cells were fixed and stained for ectoderm (β3-TUBULIN), mesoderm (αSMA), and endoderm (FOXA2) markers while sustaining high levels of transgene expression throughout the differentiation process (Figures 3F, S3F, and S3G). Similar results were observed and confirmed in engineered miPSCs (Figures S3H and S3I).

Then, we addressed the survival of SMAR vectors in engineered hiPSCs through trilineage differentiation into ectoderm (β3-TUBULIN), mesoderm (αSMA), and endoderm (FOXA2). Successful differentiation and sustained levels of transgene expression through endogenous GFP expression were observed (Figure 3G).

Finally, we investigated if SMAR vectors survive sequential reprogramming followed by differentiation. We used miPSCs and hiPSCs derived from pSMAR-modified fibroblasts, which already maintained SMAR vector expression during reprogramming (Figures 3A and 3B). Similarly, we demonstrated that SMAR vectors provide sustained and high levels of transgene expression during differentiation in hiPSCs (Figures S3J and S3K) and miPSCs (Figures S3L and S3M), and genetically modified cells at the fibroblast level can also differentiate into representatives of the three germ layers. In summary, we not only showed that our modified episomal vectors do not compromise the functional potency of iPSCs, but also, they show an unprecedented resistance to epigenetic silencing.

### SMAR vectors survive directed *in vitro* differentiation

After confirming the pluripotent capabilities of SMAR-engineered SCs and transgene maintenance during random differentiation, we sought to quantify the transgene expression of modified cells during directed differentiation.

First, mESCs engineered with pSMAR or nSMAR were subjected to hematopoietic differentiation. Three clones at different passages of stably transfected mESCs with pSMAR or nSMAR were forced to collapse into EBs under hypoxic conditions (5%O2). Unmodified mESCs were used as a control. After 6 days, successful differentiation was confirmed by the presence of a CD41 + cKIT + hematopoietic precursor population. GFP expression was quantified at the mESCs (day 0) and HSC (day 6) stage. Notably, a significant decrease in fluorescence was observed in pSMAR-labeled cells (99.76% to 76.90%), and this reduction correlated with the age of the clones, while nSMAR-labeled cells maintained GFP expression during the experiment (99.70% to 94.62%), in all clones (Figure 3H). Interestingly, the highest decrease in fluorescence corresponded with cells labeled with vectors containing bacterial sequences (pSMAR) and was less prominent when nanovectors were used (nSMAR).

Then, we quantified persistence (%GFP) and expression levels (MFI) of engineered hiPSCs during differentiation into the three germ layers. Endoderm, mesoderm, and ectoderm derivatives were analyzed and compared with undifferentiated cells (Figures 3I and S3N). In line with the murine hematopoietic differentiation, we observed a slight but significant decrease in the %GFP of pSMAR cells, particularly in the endoderm (−6.83% ± 2.75%) and ectoderm (−4.53% ± 1.30%). Additionally, we observed a slight decrease in the MFI of mesoderm and endoderm derivatives in pSMAR-hiPSCs. Notably, no significant decrease in persistence or expression was observed in nSMAR-hiPSCs.

Together, these data demonstrate the minimal impact of SMAR vectors on modified SCs, as the cells express all pluripotent markers tested and exhibit full differentiation potential. Additionally, SMAR vectors retained transgene expression during *in vitro* differentiation into derivatives of the three germ layers, as well as hematopoietic precursors.

### SMAR vectors survive *in vivo* differentiation and generate chimeric mice

**Figure 4:**
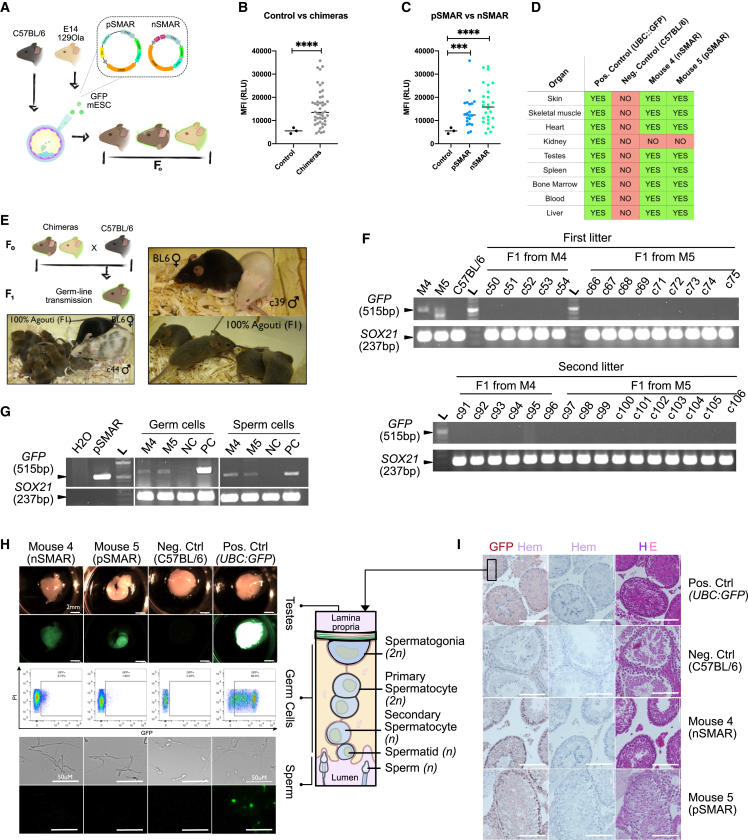
SMAR mESCs contribute to form chimeras (F0), but the genetic modification does not affect the progeny (F1) (A) Chimeras generated with pSMAR (clone v71c22) or nSMAR (clone v85c17) mESCs. Between 6 and 12, stably transfected mESCs were microinjected into C57BL/6NxB6D2F1 embryos, resulting in the formation of chimeras, as observed by the agouti/chinchilla coat color. (B) Transgenic GFP expression of ear biopsies (n = 49) at the time of weaning compared with C57BL/6N control mice (n = 3). The MFI from fluorescent images is expressed as relative light units (RLU). The statistical analysis was performed using an unpaired T test with Welch's correction (^∗∗∗∗^p < 00.001). (C) Comparison of fluorescence from ear biopsies of pSMAR (n = 23) and nSMAR (n = 26) chimeras with control mice (n = 3). The statistical analysis was performed using a Brown-Forsythe and Welsch ANOVA with Bunnett's T3 multiple comparison test (^∗∗∗^p = 00.004; ^∗∗∗∗^p < 00.001). (D) Summary of transgene expression from representative chimeric organs. See Table S3 for a complete dataset. (E) SC contribution to the germline of two male chimeric mice generated with mESCs engineered with nSMAR (mouse 4) and pSMAR (mouse 5). The males were backcrossed with C57BL/6J females and generated 100% agouti litters. (F) Genotyping PCR from tail biopsies of chimeric mice (F0) and their respective litters (F1). C57BL/6 was used as a negative control. The amplicon corresponds to a 515 bp GFP band. An internal mammalian-conserved *SOX21* sequence (237 bp) was used as an internal control. (G) PCR amplification of the transgene before (germ cells) and after (sperm) meiosis. The *GFP* amplicon corresponds to the 515 bp band. An internal mammalian conserved *SOX21* sequence (237 bp) was used as an internal control. (H) Fluorescent images and FACS analysis depicting transgene expression across gametogenesis. GFP fluorescent testis of mouse 4 (nSMAR) and mouse 5 (pSMAR). Constitutively expressing *UBC*:*GFP* mouse (Jackson lab, Pos Ctrl) and a C57BL/6N mouse (Neg Ctrl). (Leica M205FA, exposure 1s, amp gain 1.9x, digital exposure 4, scale bars = 2 mm and 50 μm). FACS analysis was performed in germinal cells from digested seminiferous tubes. The sperm was collected and imaged using a Nikon Ti microscope. Cartoon adapted from (Falcone and Hurd, 2007). (I) Immunohistochemistry of seminiferous tubules' sections (left = GFP staining with hematoxylin counterstaining, middle = unstained control, right = hematoxylin-eosin staining, scale bars = 100 μm).

An emphatic evaluation of the vectors' mitotic stability and a more stringent measure of pluripotency was performed by assessing the SCs' ability to form chimeras when injected into early-stage embryos. GFP-mESC clones (chinchilla) generated with pSMAR or nSMAR were injected into *morulae* of C57BL/6N x B6D2F1 embryos. Forty-nine chimeric pups were born, in which the presence and contribution of engineered mESCs could be observed by the agouti/chinchilla coat chimerism over the black background (Figure 4A). All pups showed varying degrees of chimerism, reaching in some cases a 100% chinchilla coat color, suggesting that a high proportion of the chimera was contributed by the genetically modified mESCs (Table S2). We then addressed the presence of SMAR vectors and GFP expression in chimeric pups by analyzing 49 ear punches taken at the time of weaning. The overall MFI was significantly higher in the chimeric biopsies compared with BL6 negative controls (Figures 4B and S4A). No difference was observed in the MFI between pSMAR- and nSMAR-generated chimeras (Figure 4C). We then confirmed via PCR that fluorescence was caused by GFP presence instead of autofluorescence, as GFP could be amplified in 26/49 biopsies (Figure S4B).

Next, we selected five chimeras to analyze the transgene expression in chimeric organs derived from the three germ layers (Figure 4D). GFP was highly expressed in the muscle, skin, and liver and to a lesser extent in the heart and kidney (Figure S4C and Table S3). More interestingly, GFP was also expressed in highly regenerating hematopoietic tissues (i.e., bone marrow, blood, and spleen). Chimeric blood ranged from 17.30% to 63.20%, while bone marrow contained between 3.53% and 56.00% GFP + cells and the chimeric spleens between 4.74% and 55.80% (Figure S4D).

Taken together, these data show the capability of SMAR vectors to survive *in vivo* differentiation from a fertilized egg to a fully developed living organism while persistently expressing the transgene. The injection of engineered mESCs into embryos allowed the generation of *bona fide* chimeras, in some cases reaching almost complete coat chimerism.

### SMAR-modified mESCs form chimeras (F0), but genetic modification is not sustained in progeny (F1)

After confirming SMAR mESCs can form chimeras and retain vector expression during cell division and differentiation both *in vitro* and *in vivo*, we assessed whether modified SCs could contribute to the germline (SC transmission) and, most interestingly, whether SMAR vectors survived meiosis and could be passed on to the offspring (vector transmission). Although SMAR vectors are known to replicate episomally and segregate during mitosis (Jenke et al., 2002; Stehle et al., 2007), their ability to persist through meiosis was unknown.

Six chimeras were bred with C57BL/6J mice, and their offspring were analyzed. Males 4 (c39) and 5 (c44), which displayed almost 100% of chinchilla coat color, showed SC transmission, as all their offspring were agouti (Figure 4E), because of the SMAR-modified cells' contribution to the germline. We investigated vector germline transmission by assessing the presence and expression of SMAR vectors in offspring tissues. We did not detect SMAR vectors (*GFP* amplification) in tail biopsies of agouti litters from mouse 4 or 5 (Figure 4F), regardless of the litter (two litters were analyzed per mice). These results suggested that episomal germline transmission was blocked in meiosis, suggesting that the vector was lost during gametogenesis, regardless of which vector was used.

The presence and expression of the vectors were evaluated before and after gametogenesis. For this, testes and sperm from these chimeras were collected and analyzed for both presence (PCR amplification) and expression (fluorescence) of SMAR vectors.

GFP was observed and amplified (Figure 4G) in the testes of both chimeras (Figure 4H top). To exclude that fluorescence was detected from the external testicular membrane or *Tunica Albuginea*, the seminiferous tubules were homogenized to isolate the germinal cells, comprising spermatogonia, spermatocytes, and spermatids. The fluorescence from germinal cells was analyzed by flow cytometry (Figure 4H middle), which revealed between 1.83% and 8.72% of GFP + cells.

Sperm collected from the epididymis was also analyzed. Surprisingly, we could amplify SMAR vectors from sperm lysates (Figure 4G), although fluorescence could only be observed in sperm from a positive control (*UBC*:*GFP*) mouse, but not from SMAR-generated chimeras (Figure 4H bottom), suggesting that the vectors are present throughout spermatogenesis but become silenced during meiosis.

We then analyzed at which stage of meiosis the loss of expression occurred. For this, we performed immunohistochemistry of the germinal epithelia (Figure 4I). Both chimeras showed GFP expression in the most peripheral cell layer (diploid spermatogonia). No GFP could be detected in more advanced meiotic cells, such as spermatocytes, spermatids, or sperm cells. The negative controls showed no GFP expression, whereas constitutively expressing GFP mice showed GFP expression across the germinal epithelia.

These data support our findings that SC pluripotency is not hampered by genetic modification with SMAR vectors, as engineered SCs can generate reproductive organs and contribute to the germline. Additionally, modified mESCs result in viable offspring, suggesting that SMAR vectors do not damage the chromosomal stability. We did not detect SMAR vectors in the F1 generation, suggesting that the vectors do not integrate.

## Discussion

A genetic engineering platform that provides safe, efficient, and persistent generation of isogenic SCs has broad application and stands as an alternative to currently used randomly integrating vectors.

Studies using retroviral-mediated modification of SCs often result in poor transduction efficiencies, short-lasting transgene expression (Zhang et al., 2002), or transgene silencing during differentiation (Herbst et al., 2012; Laker et al., 1998), which can be circumvented by adding chromosomal insulators, such as UCOE elements, resulting in maintained transgene expression during hematopoietic differentiation (Müller-Kuller et al., 2015; Pfaff et al., 2013). Other limitations of retroviral vectors include their production and limited cargo capacity (Tiscornia et al., 2006) and the inherent genotoxic risks associated with insertional mutagenesis (Hacein-Bey-Abina et al., 2003).

The DNA vectors described here represent a unique and novel platform and an additional tool for SC modification, offering an advantage not only for clinical and therapeutic applications but also for disease modeling and molecular analysis.

### The new generation of SMAR vectors

pEPI (Piechaczek et al., 1999) has been used to modify human hematopoietic progenitors, although transgene silencing was observed in murine SCs due to histone deacetylation (Papapetrou et al., 2006). We also observed a substantial decline in transgene expression in mESCs transfected with pEPI, although episomal forms could be detected in a Southern blot (Figure S1), suggesting vector silencing through similar mechanisms. We refined and improved every component of these vectors, resulting in pSMAR and nSMAR, which can efficiently transfect murine and human SCs, providing stable transgene expression and episomal persistence for up to 170 days (Figures 1 and S1). Removing potentially genotoxic sequences from the bacterial backbone from these vectors reduced perturbation of the host's transcription, improving performance (Figure 2).

### SMAR vectors—universal genetic tools

SMAR vectors can modify various primary cells at different stages of differentiation; they can modify fibroblasts while surviving cellular reprogramming, producing genetically modified iPSCs that display all expected pluripotent capabilities (Figure 3). For the first time, we demonstrate that a vector of this class can directly modify iPSCs in their pluripotent state. Additionally, SMAR vectors provide sustained transgene expression through *in vitro* differentiation into specific cell types, producing persistently expressing differentiated progeny (Figure S3). In a more stringent test, we demonstrate that SMAR vectors can survive *in vivo* differentiation, resulting in viable chimeras displaying high levels of transgene expression across their organs (Figures 4 and S4, Table S3). Finally, we demonstrate the vector's flexibility of use across different delivery platforms commonly used in the lab, including chemical or physical transfection methods, such as Neon, Amaxa, or MaxCyte electroporators (Table 2).

**Table 2:** S/MAR vectors are compatible across multiple transfection technologies

Cells	Technology	Vector	Efficiency	Viability	Conditions
hESC hiPSC	MaxCyte® ExPERT®	pSMAR	82%–85%	87%–91%	Optimization 8 1 × 10^7^ cells/ml (50 ul) 200–300ug/ml plasmid
	Lipofectamine STEM	pSMAR nSMAR	NA	NA	Clump transfection 1–2 ul LipoSTEM + 25 ul OptiMEM 500 ng plasmid + 25 ul OptiMEM
	Lipofectamine STEM	pSMAR nSMAR	25%–85%	85%–98%	5 × 10^4^ single cells/well in 24 wp 1–2 ul LipoSTEM + 25 ul OptiMEM 500 ng plasmid + 25 ul OptiMEM
NHDF	Amaxa II	pSMAR nSMAR	25%–84%	95%–96%	NHDF kit (Program P-022) 5 × 10^5^ cells 2–10 ug plasmid (100 ul)
	Neon	pSMAR nSMAR	65%–67%	95%–96%	1,650 V, 10 ms 3 pulses 1 × 10^6^ cells 2 ug plasmid (100 ul)
MEF	Amaxa II	pSMAR nSMAR	40% 54%	96% 93%	NHDF kit (Program U-020) 5 × 10^5^ cells 2–10 ug plasmid (100 ul)
mESC miPSC	Amaxa II	pSMAR nSMAR	50%–56% 55%–66%	90%–97% 90%–97%	Mouse ESC kit (Program A-013) 5 × 10^5^ cells 2–10 ug plasmid (100 ul)

### Genomic stability and generation of isogenic cells

The genomic stability of SMAR-modified SCs was demonstrated both functionally and molecularly. Cells retain all pluripotent features and differentiate *in vitro* into representatives of all germ layers, while retaining high levels of transgene expression (Figures 3 and S3), and can contribute to generating chimeras (Figures 4 and S4). Removing bacterial sequences from the vector backbone results in higher and more stable transgenic expression during differentiation (Figures 3H and 3I). SMAR vectors safely and persistently modify SCs, while delivering stable levels of transgene expression during *in vitro* (Figures 3I and S3N) and *in vivo* differentiation—including expression into regenerative hematopoietic organs (Figures 3H and S4).

Modified SCs show little transcriptomic variation, especially when bacterial sequences are removed (Figure 2). We also show that SMAR vectors are maintained as extrachromosomal entities without integration, as we could rescue circular DNA molecules and could not detect any indication of genomic integrations (Figure S1). Analysis of SMAR-generated chimeras also suggest that SMAR vectors are not integrated but are rather sustained episomally, as no vector was transmitted to offspring (Figure 4).

### SC versus vector transmission

SMAR vectors have been used to generate transgenic animals (Manzini et al., 2006) (Wagner et al., 2019), where pEPI was directly delivered using sperm-mediated gene transfer. However, the germline transmission of these vectors has not been previously investigated, and the behavior of SMAR vectors during meiosis is not yet understood. We show that SMAR-modified SCs can differentiate into functional gonads (testes), produce functional germ cells (sperm) carrying the vector, and contribute to the offspring (Figure 4). This process, which we refer to as “SC transmission,” results in viable F1, providing evidence that the SMAR engineered SCs are not damaged by the vector. However, the inheritance of episomal SMAR vectors during meiosis—“vector transmission”—does not occur. Data indicate that SMAR vectors are present and expressed in reproductive organs (testes), but that transgene expression is gradually lost in meiosis during the spermatogonia/spermatid transition, perhaps by epigenetic mechanisms involved during spermatogenesis (Schagdarsurengin et al., 2012). The result is a mature sperm cell with few silenced copies of SMAR vectors. We believe that the sperm acts as a vector shuttle, delivering copies of the DNA vector into the oocyte, but these fail to establish. Upon fertilization, SMAR vectors need to re-establish to function in this new cellular entity. However, the low copy number and the stochastic nature of establishment represent a very low chance for vector re-establishment after fertilization and contribute to the dilution of few episomal vector copies as the embryo develops, resulting in the loss of replicating SMAR vectors in the F1 generation. Further investigation of SMAR inheritance, including female oogenesis, might provide a deeper understanding of this poorly understood process.

In summary, SMAR vectors can be used as a universal genetic tool for the modification of potentially any cell type, including primary cells, which are typically refractory to genetic manipulation. These vectors can be easily produced in large amounts and can be efficiently delivered to cells with efficiencies above 60%, resulting in the safe generation of genetically engineered isogenic SCs.

## Experimental procedures

Routinely used protocols and materials are included in the supplemental information.

### DNA vectors

All DNA vectors were cloned using InFusion HD cloning (Clontech) and following the manufacturer's instructions. An amount of 100 ng of vector and 50 ng of the insert were mixed with water containing the 5x InFusion mix, containing the appropriate buffer and enzyme to allow for homologous recombination between the 15 bp of homology. It was essential that the volume of insert + vector did not exceed 7 μL. In such cases, the InFusion reaction volume was doubled. The recombination took place at 50°C for 15 min. Finally, 2.5 μL of InFusion reaction was transformed into *E*. *coli* Stellar competent cells (Clontech), following the manufacturers’ instructions.

pSMARt CAG:GFP-2A-Puro-SMAR (pSMAR): was generated in three steps. 1) pMAX_SMAR was generated by amplification of the SMAR motif from pEPI-CMV-UCOE (Hagedorn et al., 2013) with primers 1 and 2 and inserted into pMAX_coGFP (Lonza) digested with *SacI* and *PciI*. 2) pMAX_SMAR was digested with *BglII*, and the expression cassette was modified by adding a 2A-Puromycin after the coGFP (primers 3 and 4) to generate pSMARt. 3) The CMV promoter was swapped by the chimeric CAG promoter in the pSMAR vector using the primers 5 and 6 (Table S4).

nSMARt CAG:GFP-2A-Puro-SMAR (nSMAR) was generated by Nature Technology Corporation (NTX).

pSMARt SV40LT-GFP: pSMARt was digested with *BsrGI* to add the insulating Element40, amplified using primers 11 and 12. Then, the 2A-*Puromycin* from pSMARt was cut out with *BmgBI* and *XhoI* and replaced by the *SV40 large T antigen*, amplified with primers 13 and 14 (Table S4).

### Cell line generation

A range of different transfection technologies, including Neon, Amaxa, Lipofectamine STEM, and MaxCyte, was tested in this study. Comparative results are shown in Table 2.

Plasmid DNA was delivered (unless otherwise stated) by electroporation using the Amaxa II Nucleofector system (Lonza). For mESC, miPSC, and hESC, 500.000 cells were washed, trypsinized, and resuspended in Mouse ES Cell Nucleofector Kit (VPH-1001) solutions containing between 2 and 10 μg of plasmid DNA. For comparative experiments, equimolar concentrations of plasmid were delivered into cells. The programs A-013 (mESC/miPSC) and A-023 (hESC) were used. After electroporation, the cells were carefully transferred into feeder plates in media without antibiotics. After 24 h, the media were replaced by the respective complete media containing antibiotics and G418 (1 mg/ml) or 1 μg/ml Puromycin selection, if needed. The cells were kept under selection for a month, and the media were replaced every second day.

For feeder-free transfection of hiPSCs, cells were plated in small clumps at a density of 20%–30% or as 50.000 single cells in a 24-well plate. The next day, cells were transfected using Lipofectamine Stem as of manufacturers recommendation, using 2 μL or 1 μL of transfection reagent diluted in 25 μL OptiMEM, mixed with 500 ng DNA diluted in 25 μL OptiMEM. For comparative experiments, equimolar concentrations of vectors were used. For establishment using antibiotics, 24 h after transfection, cells were selected with media containing 0.5 μg/ml Puromycin and kept under selection for two weeks. For establishment with FACS, GFP + cells were sorted and further cultured 6, 12, 28, and 44 dpt. Media were replaced three times per week.

For fibroblasts (MEFs and HDFs), 500.000 cells were washed, trypisinized and resuspended in NHDF Electroporation Kit (VPD-1001) solutions containing between 2 and 10 μg of plasmid DNA. For comparative experiments, equimolar concentrations of plasmid were delivered into cells. Programs U-020 (MEFs) or P-022 (HDFs) were used in the Amaxa Nucleofector II device (Lonza). Finally, the electroporated cells were gently pipetted and transferred into a gelatin-coated 6-well plate with DMEM (Gibco) containing 10% FCS (Gibco) without selection nor antibiotics and allowed to recover. After 24 h, antibiotics were added to the media as well as G418 (1 mg/ml) or 1 μg/ml Puromycin selection, if needed. The cells were kept under selection for a month, and the media were replaced every second day.

### Quantification and data analysis

Unless otherwise stated, statistical analysis was performed using Graphpad Prism 8. T test (with or without Welch's correction) was used for statistical analysis unless otherwise specified. For all statistical analyzes, a value of p <0.05 was considered statistically significant.

### Contact for reagents and resource sharing

Further information and requests for resources and reagents should be directed to the Lead Contact, Richard Harbottle (r.harbottle@dkfz.de).

### Data and software availability

The microarray data discussed in this publication have been deposited in NCBI's gene Expression Omnibus (Edgar et al., 2002) and are accessible through GEO Series accession number GSE142299.

## Author contributions

R.P.H. and A.R.M. formulated the concept. M.Bo. and A.R.M. designed and generated the vectors. A.R.M. carried out the murine molecular and biological experiments, as well as *in vitro* and *in vivo* experiments. M.U. carried out the human molecular and biological experiments. M.Bu., S.S., and A.R.M. performed the hematopoietic differentiation and analysis of hematopoietic tissues. L.B. generated hESC lines for RNAseq, and J.P. performed the bioinformatic analysis. F.v.H. performed the pronuclear and blastocyst injections. K.M.D. processed the transgenic tissues and provided technical support. T.M. and M.M. provided technical support and scientific advice. A.R.M., M.U., and R.P.H. prepared the manuscript.

## Conflicts of interests

M.Bo. and R.H. have patent applications related to this work filed by the DKFZ and NTC (WO2019057774A1, filed 19 September 2018, published 28 March 2019). (WO2019060253A1, filed 17 September 2018, published 28 March 2019).

A.R.M. currently works at MaxCyte Inc.

## Supporting citations

The following references appear in the supplemental information: Blighe et al., 2021; Evans and Kaufman, 1981; Gu et al., 2016; Hooper et al., 1987; Koressaar and Remm, 2007; Koutsoudakis et al., 2006; Maetzig et al., 2014; Mulder et al., 2020; Neufang et al., 2001; Phipson et al., 2016; Schindelin et al., 2012; Ritchie et al., 2015; Shi et al., 2010; Tesar, 2005; Untergasser et al., 2012; Varisli et al., 2013; Willemsen et al., 2017; Zufferey et al., 1998; Zufferey et al., 1997.

## Supplemental information

Document S1. Supplemental experimental procedures, Figures S1–S4, and Tables S2 and S3

Table S1. Downregulated and upregulated genes upon delivery of pSMAR and nSMAR vectors

Table S4. Reagents, resources, and sequences

Document S2. Article plus supplemental information

## Footnotes

Supplemental information can be found online at https://doi.org/10.1016/j.stemcr.2021.11.011.
