# Supplementary material for: An episomal DNA vector platform for the persistent genetic modification of pluripotent stem cells and their differentiated progeny
Source: Stem Cell Reports. 2021 Dec 22;17(1):143–58. doi: 10.1016/j.stemcr.2021.11.011 (PMC8758943; doi:10.1016/j.stemcr.2021.11.011)
Supplement: Document S1. Supplemental experimental procedures, Figures S1–S4, and Tables S2 and S3 [file mmc1.pdf]

Stem Cell Reports, Volume *17*

Supplemental Information

An episomal DNA vector platform for the persistent genetic modification of pluripotent stem cells and their differentiated progeny

Alicia Roig-Merino, Manuela Urban, Matthias Bozza, Julia D. Peterson, Louise Bullen, Marleen Büchler-Schäff, Sina Stäble, Franciscus van der Hoeven, Karin Müller-Decker, Tristan R. McKay, Michael D. Milsom, and Richard P. Harbottle

SUPPLEMENTAL INFORMATION

Figure S1

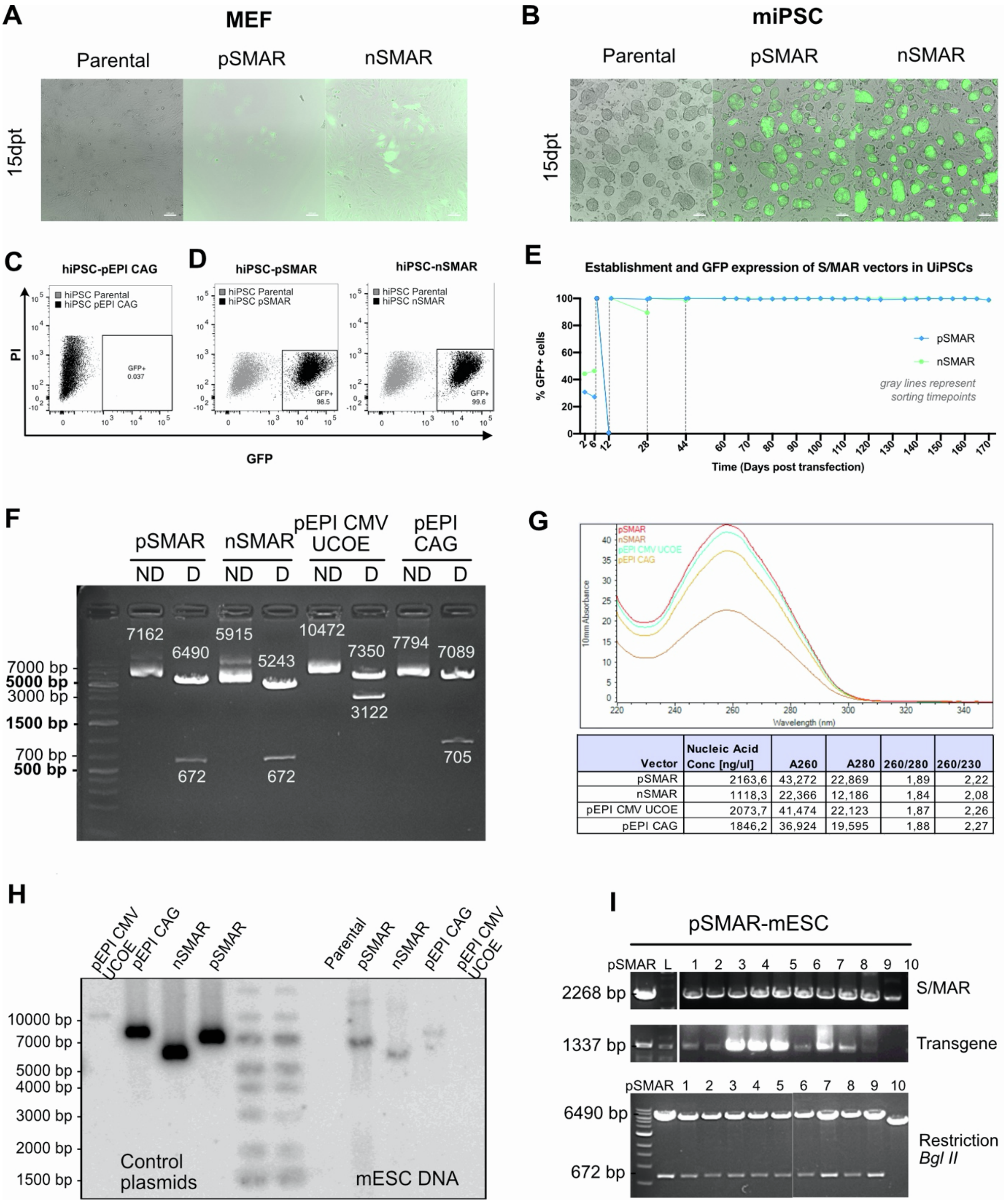

Vector	Nucleic Acid Conc [ng/ul]	A260	A280	260/280	260/230
pSMAR	2163,6	43,272	22,869	1,89	2,22
nSMAR	1118,3	22,366	12,186	1,84	2,08
pEPI CMV UCOE	2073,7	41,474	22,123	1,87	2,26
pEPI CAG	1846,2	36,924	19,595	1,88	2,27

Figure S1 : Confirmation of vector integrity and performance in other primary cells

(A) MEF cell line generated with pSMAR and nSMAR. Fluorescent images of %GFP+ cells and MFI, 15 days after transfection (Scale bars = 100µm).
(B) miPSC cell line generated with pSMAR and nSMAR. Fluorescent images of %GFP+ cells and MFI, 15 days after transfection (Scale bars = 100µm).
(C) Flow Cytometry analysis of outgrowing hiPSC-pEPI-CAG cells after G418 selection.
(D) NHDF-derived hiPSCs generated with pSMAR and nSMAR. Flow Cytometry analysis of GFP expression in parental and modified hiPSCs, 3 months after transfection and >2 months without selection.
(E) Passive establishment (antibiotic selection-free) and GFP expression of pSMAR and nSMAR vectors in urinary-derived hiPSC (UiPSC) over a period of 170 days. The gray lines represent FACS sorting timepoints.
(F) DNA electrophoresis assessing vector integrity. ND=non-digested, D=digested with *BglII*.
(G) Spectrophotometric analysis of DNA vector quality
(H) Southern Blot analysis of mESCs showing the integrity of the DNA vectors
(I) Plasmid rescue of pSMAR from stably transfected mESCs. PCR amplification of the SMAR motif and transgene (n=10 bacterial clones)

Figure S2

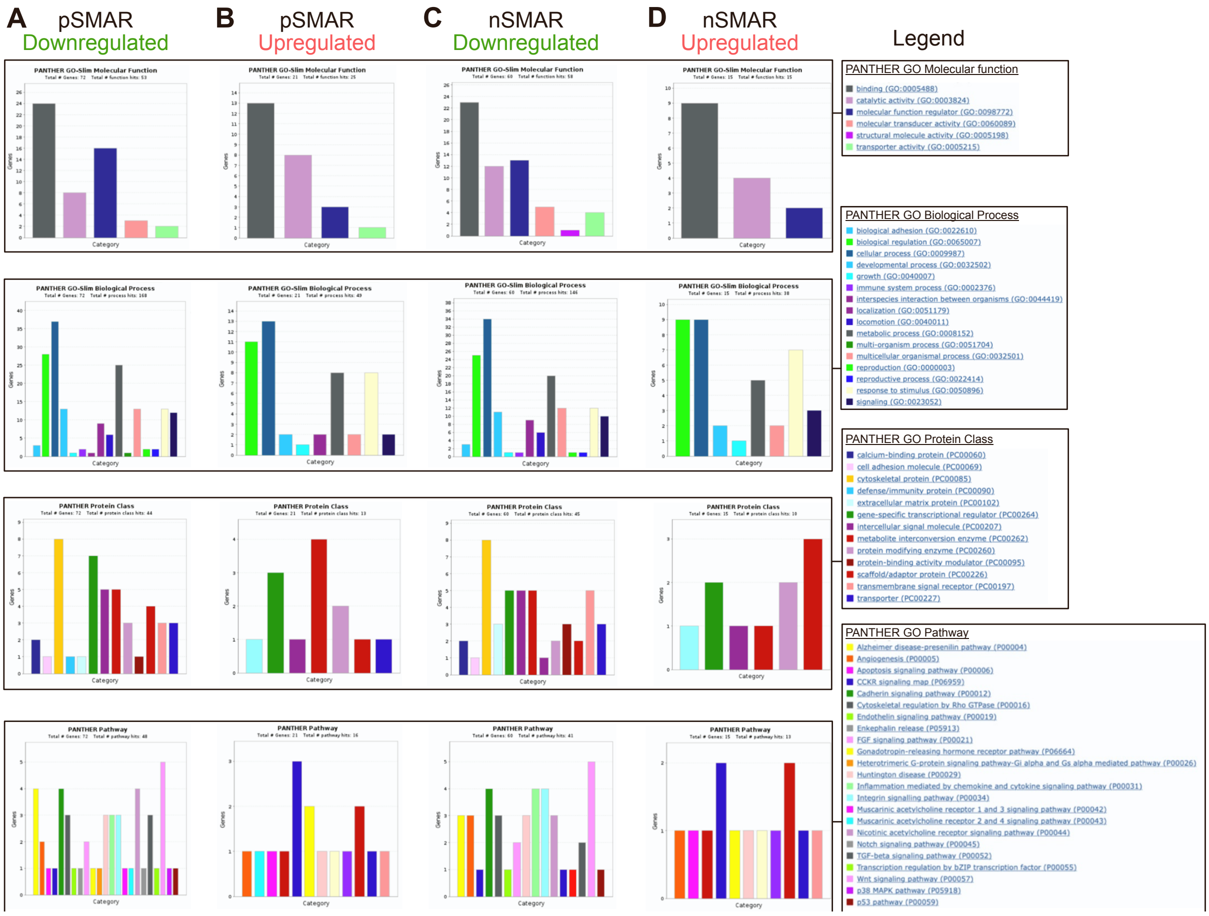

Figure S2: SMAR NanoVectors have a minimal impact on the cells' transcriptome

GO TERM analysis performed on the full list of significantly down or upregulated genes in pSMAR or nSMAR transfected hESCs using GO terms and the PANTHER classification system.

Figure S3

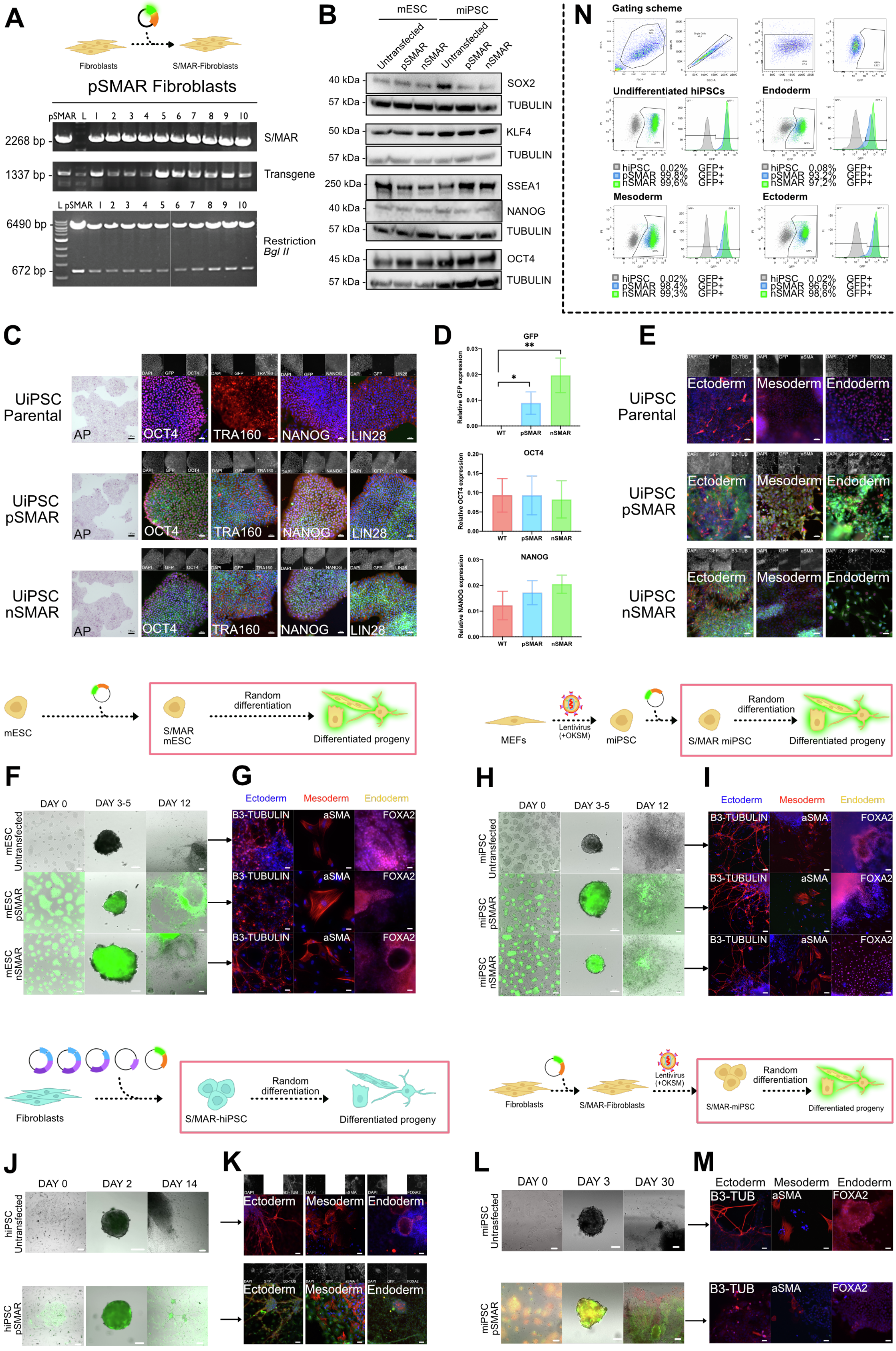
A
Fibroblasts
S/MAR-Fibroblasts
pSMAR Fibroblasts
pSMAR L 1 2 3 4 5 6 7 8 9 10
2268 bp
S/MAR
1337 bp
Transgene
L pSMAR 1 2 3 4 5 6 7 8 9 10
6490 bp
672 bp
Restriction Bgl II
B
mESC
miPSC
Untransfected
pSMAR
nSMAR
40 kDa
SOX2
57 kDa
TUBULIN
50 kDa
KLF4
250 kDa
SSEA1
NANOG
45 kDa
OCT4
N
Gating scheme
Undifferentiated hiPSCs
hiPSC 0,02% GFP+
pSMAR 99,8% GFP+
nSMAR 99,6% GFP+
Endoderm
hiPSC 0,08% GFP+
pSMAR 93,2% GFP+
nSMAR 97,2% GFP+
Mesoderm
hiPSC 0,02% GFP+
pSMAR 98,4% GFP+
nSMAR 99,3% GFP+
Ectoderm
hiPSC 0,02% GFP+
pSMAR 96,6% GFP+
nSMAR 98,6% GFP+
C
UiPSC Parental
UiPSC pSMAR
UiPSC nSMAR
AP
OCT4
TRA160
NANOG
LIN28
D
GFP
Relative GFP expression
OCT4
Relative OCT4 expression
NANOG
Relative NANOG expression
WT
pSMAR
nSMAR
E
Ectoderm
Mesoderm
Endoderm
mESC
S/MAR mESC
Random differentiation
Differentiated progeny
MEFs
Lentivirus (+OKSM)
miPSC
S/MAR miPSC
F
DAY 0
DAY 3-5
DAY 12
mESC Untransfected
mESC pSMAR
mESC nSMAR
G
B3-TUBULIN
aSMA
FOXA2
H
miPSC Untransfected
miPSC pSMAR
miPSC nSMAR
I
Fibroblasts
S/MAR-hiPSC
S/MAR-Fibroblasts
S/MAR-miPSC
J
DAY 2
DAY 14
hiPSC Untransfected
hiPSC pSMAR
K
L
DAY 3
DAY 30
miPSC Untrasfected
miPSC pSMAR
M
B3-TUB

Figure S3: SMAR vectors remain episomal and do not impair Stem Cells' pluripotency

(A) Plasmid rescue of pSMAR from stably transfected MEFs. PCR amplification of the SMAR motif and transgene. Restriction digestion of 10 clones with BglII
(B) Western Blot of pluripotency markers of mESCs and miPSCs generated from CF1-MEFs upon transduction with pWPI 4in1 Lentiviral vector
(C) Pluripotency staining of human UiPSCs reprogrammed using EBNA vectors and modified at the iPSC level (Scale bars = 100μm).
(D) Quantification of GFP and pluripotency markers in UiPSC. qRT-PCR analysis of transgene and the pluripotency markers Oct4 and Nanog, relative to GAPDH. 1-WAY ANOVA and Unpaired T-Tests were used for statistical analysis of three independent differentiations of the same cells (n=3, **p-val = 0,0073, *p-val=0,0236).
(E) Trilineage differentiation of UiPSC modified with pSMAR or nSMAR at the iPSC level (b3-Tubulin, ectoderm; aSMA, mesoderm; FoxA2, endoderm, Scale bars = 100μm).
(F) Random differentiation via EBs of parental and pSMAR-mESC, modified at the mESC level (Scale bars = 100μm).
(G) Exemplary immunofluorescence staining of germ layer derivatives (b3-Tubulin, ectoderm; aSMA, mesoderm; FoxA2, endoderm) from parental and pSMAR-mESCs (Scale bars = 100μm).
(H) Random differentiation via EBs of parental and pSMAR-miPSCs, derived from CF1-MEFs modified at the fibroblast level and reprogrammed using 4in1 Lentivirus (Scale bars = 100μm).
(I) Exemplary immunofluorescence staining of germ layer derivatives (b3-Tubulin, ectoderm; aSMA, mesoderm; FoxA2, endoderm) from parental and pSMAR-miPSCs (Scale bars = 100μm).
(J) Random differentiation via EBs of parental and pSMAR-hiPSC derived from NHDFs modified at the fibroblast level, and reprogrammed using EBNA vectors (Scale bars = 100μm).
(K) Exemplary immunofluorescence staining of germ layer derivatives (b3-Tubulin, ectoderm; aSMA, mesoderm; FoxA2, endoderm) from parental and pSMAR-hiPSCs (Scale bars = 100μm).
(L) Random differentiation via EBs of parental and pSMAR-miPSC, modified at the fibroblast level and reprogrammed using 4in1 Lentivirus (dTom) (Scale bars = 100μm).
(M) Exemplary immunofluorescence staining of germ layer derivatives (b3-Tubulin, ectoderm; aSMA, mesoderm; FoxA2, endoderm) from parental and pSMAR-miPSCs (Scale bars = 100μm).
(N) Quantification of transgene expression in differentiated progeny of genetically modified hiPSCs Gating scheme for GFP-quantification experiments. Dot plots and histograms depicting persistance of GFP expression from pSMAR or nSMAR engineered hiPSC, separated by germ-layer

Figure S4

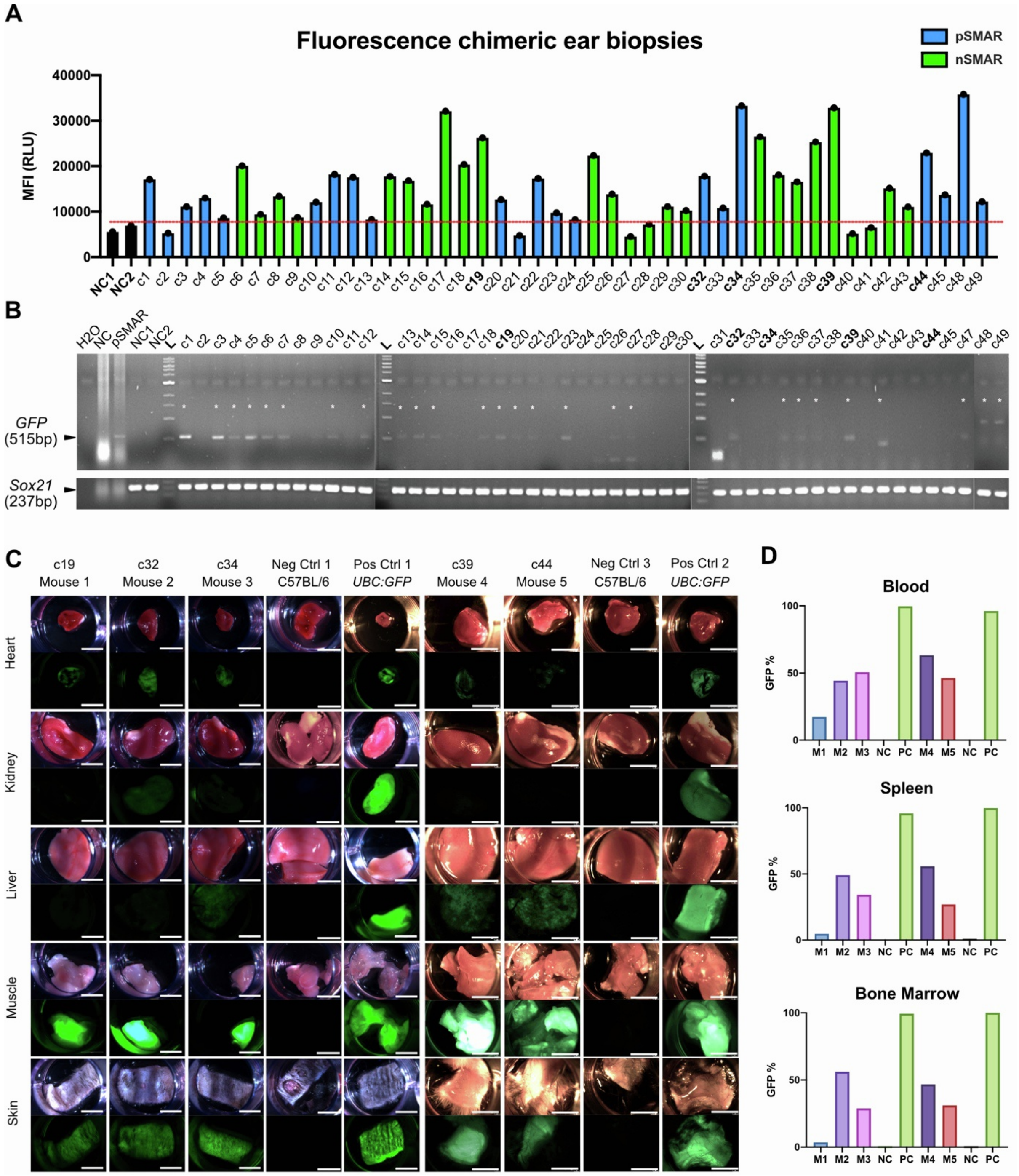

A
Fluorescence chimeric ear biopsies
pSMAR
nSMAR
MFI (RLU)
40000
30000
20000
10000
0
NC1 NC2 c1 c2 c3 c4 c5 c6 c7 c8 c9 c10 c11 c12 c13 c14 c15 c16 c17 c18 c19 c20 c21 c22 c23 c24 c25 c26 c27 c28 c29 c30 c32 c33 c34 c35 c36 c37 c38 c39 c40 c41 c42 c43 c44 c45 c48 c49
B
H2O NC pSMAR NC1 NC2 L c1 c2 c3 c4 c5 c6 c7 c8 c9 c10 c11 c12 L c13 c14 c15 c16 c17 c18 c19 c20 c21 c22 c23 c24 c25 c26 c27 c28 c29 c30 L c31 c32 c33 c34 c35 c36 c37 c38 c39 c40 c41 c42 c43 c44 c45 c47 c48 c49
GFP (515bp)
Sox21 (237bp)
C
c19 Mouse 1
c32 Mouse 2
c34 Mouse 3
Neg Ctrl 1 C57BL/6
Pos Ctrl 1 UBC:GFP
c39 Mouse 4
c44 Mouse 5
Neg Ctrl 3 C57BL/6
Pos Ctrl 2 UBC:GFP
Heart
Kidney
Liver
Muscle
Skin
D
Blood
Spleen
Bone Marrow
GFP %
100
50
0
M1 M2 M3 NC PC M4 M5 NC PC

Figure S4: SMAR vectors survive in vivo differentiation and contribute to form chimeras (related to Table S3)

(A) Transgenic (GFP) expression measured from fluorescent microscopy images of ear biopsies of born chimeric pups (n=49) at the time of weaning, and C57BL/6N control mice (n=2). The mean fluorescence is expressed as RLU. The red line represents the threshold for autofluorescence background. Bolded animals were selected for further analysis

(B) Genotyping PCR from ear biopsies of chimeric mice (n=49) at the time of weaning, and C57BL/6N control mice (NC, n=2). Water (H_2O) was used as technical negative control. DNA from HEK293 wildtype or transfected with pSMAR vector (GFP- and GFP+ lysate) were used as biological controls. The transgene (GFP) amplicon corresponds to a 515bp band. An internal mammalian conserved Sox21 sequence (237bp) was used as internal control.

(C) Fluorescent images of chimeric organs derived from the three germ layers in the selected chimeras (c19, c32, c34, c39 and c44). The experiment was performed in 2 days. Constitutively expressing UBC:GFP mice (Jackson lab) were used as positive controls and a C57BL/6N mice as negative controls. (Leica M205FA, exposure 1s, amp gain 1,9x, digital exposure 4, Scale bars = 5mm).

(D) Flow Cytometry analysis of transgene expression in blood, bone marrow and spleen. A panel of surface markers was used to subgroup GFP+ blood cells into T cells (CD4, CD8a), B cells (B220) and Myeloid cells (CD11b). The pan-blood surface marker CD45 was used to gate GFP+ viable cells in bone marrow and spleen (The experiment was performed in 2 days with the respective 2 sets of controls)

Table S1

Table S 1: Downregulated and Upregulated genes upon delivery of pSMAR and nSMAR vectors

Table S1 is provided as a separate excel file

Table S2

Table S 2:Coat chimerism of pSMAR and nSMAR chimeras

mESC clone	coat color	% chimerism
pSMAR 71c22	Agouti/chinchilla/black	40% Chinchilla
pSMAR 71c22	Agouti/chinchilla/black	5% Chinchilla
pSMAR 71c22	Agouti/chinchilla/black	65% Chinchilla
pSMAR 71c22	Agouti/chinchilla/black	40% Chinchilla
pSMAR 71c22	Agouti/chinchilla/black	40% Chinchilla
pSMAR 71c22	Agouti/chinchilla/black	5% Chinchilla + 50% Agouti
pSMAR 71c22	Agouti/chinchilla/black	20% Chinchilla
pSMAR 71c22	Agouti/chinchilla/black	15% Chinchilla
pSMAR 71c22	Agouti/chinchilla/black	10% Chinchilla
pSMAR 71c22	Agouti/chinchilla/black	5% Chinchilla
pSMAR 71c22	Agouti/chinchilla/black	90% Agouti
pSMAR 71c22	Agouti/chinchilla/black	30% Agouti
pSMAR 71c22	Agouti/chinchilla/black	30% Agouti
pSMAR 71c22	Agouti/chinchilla/black	30% Chinchilla
pSMAR 71c22	Agouti/chinchilla/black	20% Chinchilla
pSMAR 71c22	Agouti/chinchilla/black	10% Chinchilla
pSMAR 71c22	Agouti/chinchilla/black	50% Agouti
nSMAR 85c17	Agouti/chinchilla/black	15% Chinchilla
nSMAR 85c17	Agouti/chinchilla/black	10% Chinchilla
nSMAR 85c17	Agouti/chinchilla/black	5% Chinchilla
nSMAR 85c17	Agouti/black	70% Agouti
nSMAR 85c17	Agouti/black	20% Agouti
nSMAR 85c17	Agouti/chinchilla/black	60% Chinchilla
nSMAR 85c17	Agouti/chinchilla/black	30% Chinchilla
nSMAR 85c17	Agouti/chinchilla/black	5% Chinchilla
nSMAR 85c17	Agouti/black	100% Agouti
nSMAR 85c17	Agouti/black	50% Agouti
nSMAR 85c17	Agouti/black	20% Agouti
nSMAR 85c17	Agouti/chinchilla/black	55% Chinchilla
nSMAR 85c17	Agouti/chinchilla/black	30% Chinchilla
nSMAR 85c17	Agouti/chinchilla/black	5% Chinchilla + 65% Agouti
nSMAR 85c17	Agouti/chinchilla/black	90% Chinchilla
nSMAR 85c17	Agouti/chinchilla/black	65% Chinchilla
nSMAR 85c17	Agouti/chinchilla/black	40% Chinchilla
nSMAR 85c17	Agouti/chinchilla/black	30% Chinchilla
nSMAR 85c17	Agouti/chinchilla/black	20% Chinchilla
nSMAR 85c17	Agouti/chinchilla	95% Chinchilla
nSMAR 85c17	Agouti/chinchilla	90% Chinchilla
nSMAR 85c17	Agouti/chinchilla/black	85% Chinchilla
nSMAR 85c17	Agouti/chinchilla/black	50% Chinchilla
nSMAR 85c17	Agouti/chinchilla/black	30% Chinchilla
nSMAR 85c17	Agouti/chinchilla/black	5% Chinchilla + 50% Agouti

Table S3

Table S 3: Transgenic expression in chimeric organs

Bolded mice were selected as examples to represent in the main figures and were analyzed for germline transmission. Transgenic expression of heart, kidney, liver, skeletal muscle, skin, testis and sperm, was assessed using fluorescence microscopy. Transgenic expression and quantification of GFP in spermatogonias, blood, spleen and bone marrow, was assessed using flow cytometry

Mouse ID	c19 Mouse 1	c32 Mouse 2	c34 Mouse 3	c39 Mouse 4	c44 Mouse 5	Neg Ctrl 1	Neg Ctrl 2	Neg Ctrl 3	Pos Ctrol 1	Pos Ctrol 2
Vector	nSMAR	pSMAR	nSMAR	nSMAR	pSMAR	BL/6N	BL/6N	BL/6N	UBC:GFP	UBC:GFP
Heart	yes	yes	yes	**yes**	**yes**	no	n/a	**no**	yes	**yes**
Kidney	no	yes	no	**no**	**no**	no	n/a	**no**	yes	**yes**
Liver	no	yes	yes	**yes**	**yes**	no	n/a	**no**	yes	**yes**
Muscle	yes	yes	yes	**yes**	**yes**	no	n/a	**no**	yes	**yes**
Skin	yes	yes	yes	**yes**	**yes**	no	n/a	**no**	yes	**yes**
Testis	n/a	n/a	n/a	**yes**	**yes**	n/a	n/a	**no**	n/a	**yes**
Sperm	n/a	n/a	n/a	**no**	**no**	n/a	n/a	**no**	n/a	**yes**
Spermatogonias	n/a	n/a	n/a	**8,72%**	**1,83%**	n/a	n/a	**0,22%**	n/a	**82,60%**
Blood	17,30%	44,30%	50,60%	**63,20%**	**46,30%**	0,04%	0,05%	**0,26%**	99,70%	**96,10%**
Bone Marrow	3,53%	56,00%	28,80%	**46,70%**	**31,00%**	0,09%	0,09%	**0,11%**	99,40%	**100,00%**
Spleen	4,74%	49,10%	34,20%	**55,80%**	**26,90%**	0,06%	0,07%	**0,40%**	95,90%	**99,90%**
Testis	n/a	n/a	n/a	**yes**	**yes**	n/a	n/a	**no**	n/a	**yes**
Sperm	n/a	n/a	n/a	**yes**	**yes**	n/a	n/a	**no**	n/a	**yes**

SUPPLEMENTAL METHODS

Table S 4: Reagents, Resources and Sequences

Table S4 is provided as a separate excel file

DNA sequencing

Sequencing of DNA plasmids was performed by GATC Biotech AG (Eurofins Genomics). For this, 5 µl of >100 ng/µl plasmid DNA was mixed with 5 µl of the appropriate primer at a final concentration of 5 µM.

Fluorescence microscopy

To image engineered SCs with SMAR vectors, GFP expression was imaged with a 10X or 20X objective in a Nikon Eclipse Ti/X-Cite 120Led microscope.
To detect the presence of SMAR vectors in the organs of chimeric mice, GFP was imaged in sections of heart, kidney, liver, skin, skeletal muscle and testis were with a motorized fluorescent stereomicroscope (Leica M205 FA. Exposure 1 sec, Amp gain 1,9x).

Immunofluorescence

Stem cell colonies or differentiating structures were washed twice in cold PBS, fixed with 100% methanol for seven min at -20˚C followed by a quick rinse in ice-cold acetone for 20 sec (note that this fixation method quenches endogenous GFP fluorescence). Then, the cells were permeabilized with 0.1% Tween20 in PBS (PBST) for five min at room temperature and washed three times with cold PBST. Blocking was done in PBS with 1% FCS, 0.5% BSA, 0.1%TritonX100 for 30 min at room temperature. The primary antibody was diluted in blocking solution and incubated at 4˚C over night. Thereafter, samples were washed three times with blocking solution and then incubated with the respective secondary Alexa-Fluor-conjugated antibodies (Donkey anti-Mouse 594 and Donkey anti-Rabbit 647,1:1000 Abcam) and 2 µg/ml DAPI (Sigma) as a nuclear counterstaining for 1h at room temperature and protected from the light. Finally, the stainings were imaged with a 20x objective in a Nikon Eclipse Ti/X-Cite120Led microscope.
To enable endogenous GFP imaging, the IF protocol was changed to fixation with 4% PFA (Himedia) and all steps were performed under protection from light. Furthermore, cells were permeabilized using PBST for five min and washed 3x for 5min with 0.1% Triton-X-100 in PBS. Blocking was performed with 3% BSA in PBST for 1 h at room temperature. Primary antibodies were diluted in this blocking buffer and incubated over night at 4˚C. The next day, cells were washed three times with PBS and incubated with the respective secondary antibody coupled to an Alexa-Fluor and DAPI for 1 h at room temperature before three further washings with PBS and imaging.
For pluripotency stainings, the cells were incubated with the following primary antibodies diluted in the respective blocking buffer. For **murine SCs:** rabbit polyclonal OCT4 (Abcam, 1:500), rabbit polyclonal NANOG (Abcam, 1:500), mouse monoclonal SSEA1 (Santa Cruz, 1:200), rabbit polyclonal SOX2 (Merck, 1:500, rabbit polyclonal KLF4 (Santa Cruz, 1:200)) and for **human SCs:** (mouse monoclonal TRA-160 (Santa Cruz, 1:100), goat polyclonal OCT-3/4 (Santa Cruz, 1:100), mouse monoclonal LIN28 (Santa Cruz, 1:50) rat monoclonal SSEA3 (Santa

Cruz, 1:100), mouse monoclonal SSEA4 (Santa Cruz, 1:100) and mouse monoclonal NANOG (Santa Cruz, 1:150) were used.
For differentiating structures from the **three germ layers**, the primary antibodies used were: mouse monoclonal β3-TUBULIN (Santa Cruz, 1:100) for ectoderm, mouse monoclonal αSMA [(Santa Cruz, 1:100) for methanol/acetone fixation or (Invitrogen, 1:200) for PFA fixation] for mesoderm and mouse monoclonal FOXA2 (Santa Cruz, 1:100) for endoderm.

Immunohistochemistry and HE stainings

Organs derived from the three germ layers from chimeric and transgenic pups, as well as *UBC:GFP* mice (Jackson lab) and C57BL/6J were fixed in 4% PFA in PBS for 24h, paraffin embedded and sectioned into 5 µm thick specimens and dried at 37˚C for 2h. Prior to staining, the tissue sections were deparaffinized in xylol (twice for 10 min), and rehydrated with consecutive five min washes in 100%, 100%, 96%, 80%, 70% ethanol and finally in distilled water. Routine Hematoxylin-Eosin (HE) staining was as previously described (Neufang et al., 2001).
For immunohistochemistry, the epitopes were exposed by damp heat-induced epitope retrieval by boiling the samples 15 min in a steam pot with citrate buffer pH 6.0, followed by a 30 min cooling period and a rinse with water. Then the sections were blocked with Avidin/Biotin (Avidin/Biotin blocking kit, SP-2001, Vector Laboratories) following the manufacturers' instructions. Then, the samples were incubated with the primary antibody rabbit anti-coGFP (Abcam, 1:500) diluted in Dako Real antibody diluent (Dako) for 30 min at room temperature followed by a rinse in PBST. The secondary antibody (Goat anti-rabbit from the Dako Peroxidase/AEC detection kit, Dako) was incubated for 20 min at room temperature. The endogenous peroxidase activity was blocked by incubation in Dako Real Peroxidase blocking solution (Dako) for five min. Then, the streptavidin-peroxidase HRP (Dako) was added and incubated for 20 min. Finally, the chromogen was added and the reaction was monitored under the microscope. The sections were counterstained for one min with hematoxylin, rinsed in tap water and mounted in Aquatex mounting media.

Microarray

RNA from hESC pellets was extracted using RNeasy Mini Kit (Qiagen), following the manufacturer's instructions. DNaseI treatment was performed before downstream applications (Ambion DNA-free Kit, Invitrogen), following the manufacturer's instructions. The array was performed in the DKFZ Genomic and Proteomic Core Facility using the IlluminaHuman12 chip and the normalization across the samples was performed there.
For the analysis, biological triplicates were used, and the gene expression was background corrected, quantile normalized, and $\log_2$ transformed using the Limma package from R (Ritchie et al., 2015; Shi et al., 2010). Linear modelling was performed and the Empirical Bayes method was used to assess differential expression (Phipson et al., 2016). *P* values were adjusted using the Benjamini-Hochberg adjustment method. Top differentially expressed genes between pairwise comparisons were determined using F-statistics and top differentially expressed genes within a pairwise comparison were determined using B-statistics. Median was used as a clustering method for hierarchical clustering as it gave the largest cophenetic correlation coefficient from the 8 methods tested. Volcano plots were designed using EnhancedVolcano from R (Blighe et al., 2019) with using an adjusted *P* value<0.05 ($-\log_{10}P$ of 1.3) and a FC>2 ($\log_2$FC of 1). Venn diagrams between each pairwise

comparison were adjusted with a P value<0.05 and FC> 2. Hierarchical clustering was performed using Euclidean as a distancing measure and Median as a clustering method. The average normalized expression from the top 100 differentially expressed genes, determined by F values, was plotted for each treatment. Hierarchical clustering heat maps were designed using ComplexHeatmap from R (Gu et al., 2016).

Animals care and use

Mice were kept in IVC cages under SPF conditions in the central animal facility at the German Cancer Research Center (DKFZ, Heidelberg). All animal experiments were performed following institutional and governmental regulations and were approved by the local authorities (Regierungspräsidium Karlsruhe, Germany). Mice of both sexes were used, and experimental mice were housed with 4-5 mice per greenline cage. All mice were immune-competent and healthy. Tail biopsies or ear punches were taken at the time of weaning and were used for genotyping. Necropsy was done under license DKFZ 345.

Derivation and culture of mES cells

The derivation of mESCs was done according to (Tesar, 2005) in the presence of 2i (**GSK3 inhibitor** CHIR99021, **MEK inhibitor** PD0325901)**.** ESC culture was done according to the protocols at www.eummcr.org
E14 129Ola mESCs were obtained from the EMBL, Heidelberg) and established in culture as described (Evans and Kaufman, 1981; Hooper et al., 1987)

Embryo collection

To obtain fertilized oocytes (zygotes), 5-8-week-old females were superovulated upon intraperitoneal hormone administration: 7 I.U. gonadotropin (PMSG, Pregnant Mares serum gonadotropin), 46-48h later 7 I.U. choriogonadotropin (hCG, human chorionic gonadotropin), each dissolved in 0.1 ml of physiological saline. At least one hour after the last hormone administration, the females were paired with males of the same strain. Females with vaginal plug (VP) were sacrificed the day after by cervical dislocation, their fallopian tubes were removed, and the eggs were rinsed from the ampulla.
To obtain blastocysts, VP-positive females were sacrificed by cervical dislocation 2,5 or 3.5 days after mating. The fallopian tubes and part of the uterus were removed and the morulae (E2.5) or blastocysts (E3.5) were isolated by flushing of the fallopian tube/uterus. Transgenic mESCs were injected into the blastocoel of E3.5 blastocysts.

Blastocyst injections

Blastocyst injections were performed under the License Number G-148/13 by the DKFZ Transgenic Service. 129Ola E14-1 Mouse Embryonic Stem Cells (mESCs) were electroporated *in vitro* as described below, either with pSMAR-CAG:GFP-2A-Puro-SMAR (pSMAR); or nSMAR-CAG:GFP-2A-Puro-SMAR (nSMAR). Electroporated cells were cultured as described and between 6 - 12 stem cells were injected into either *morulae* (E2.5) of CD1 embryos or blastocysts (E3.5) of C57BL/6N x B6D2F1 embryos. On the day after injection (for morula-injection or on the day of injection (for blastocysts), the embryos were transferred into pseudo-pregnant females (CD1) that were previously mated with sterile males (CD1). The

embryos were brought to term, and the pups were checked for coat chimerism as well as for GFP expression.

Pronuclear injections

Pronuclear injections were performed under the License Number: G-97/12 by the DKFZ Transgenic Service.
Before injection, 100 µl of plasmid DNA (vector 71) at a concentration 1000 ng/µl were filtered through a Millipore Millex-GV 0.22 µm by using a disposable 1ml syringe and with an air-bubble to fill up the rest of the volume. Then, 50 µl of filtered DNA was placed on top of a floating Millipore membrane VMWPO2500 (0.025 µm pore size) and desalted via dialysis in 50 - 100 ml of Dialysis Buffer (100 ml Ampuwa ddH_2O, 10 mM Tris and 0.1 mM EDTA) for 3 hours at 4˚C to avoid evaporation and DNA loss. After 3h, the DNA drop was carefully recovered, and both quality and quantity of DNA were assessed via spectrophotometry (Nanodrop) and Agarose Gel electrophoresis. DNA recoveries ranging from 70 - 80% were achieved. The DNA solution was stored at -20˚C or used for downstream applications.
The day of injection, 1 - 2 picoliters of plasmid DNA at a concentration of 1 – 3 ng/µl were injected into the pronucleus of E0.5 murine C57BL/6N zygotes. After injection, the zygotes were transferred into pseudo-pregnant females (CD1) that were previously mated with sterile males (CD1). Some embryos (n=12) were kept in culture in KSOM (Millipore) to check for transgene toxicity and developmental abnormalities. The rest of the embryos were brought to term and the pups were checked for GFP expression.

Transgenic sperm collection

Sperm collection was performed by the DKFZ Cryopreservation Service.
Two BL/6J males were euthanized with CO_2 inhalation and cervical dislocation. After an abdominal incision and a peritoneal cut, the testes and *cauda epididymis* were exposed, cut, and separately placed in PBS. With the aid of a stereomicroscope and tweezers, the *cauda epididymis* was cut, and the sperm was released into the buffer. Modified protocol from (Varisli et al., 2013).
For imaging, 10 µl of sperm in PBS were placed on a slide and covered with a coverslip. Then recordings of motility or fluorescence images were taken using a Nikon Ti microscope and a 20x objective. When the sperm could not be freshly imaged, it could be kept alive and motile at room temperature for up to 2 hours. The remaining sperm was pelleted and frozen for total DNA extraction and downstream applications.

Human cells

Normal human dermal fibroblasts (NHDFs, 3-year old male) were cultured on gelatin-coated dishes (0.1% gelatin in distilled water) and grown in DMEM (4500 mg/L glucose, L-glutamine, sodium bicarbonate, without sodium pyruvate), supplemented with 10% FBS (Gibco), 1% Pen/Strep (Gibco) and 1% NEAA (Gibco). The cells were cultured in a humidified atmosphere at 37˚C and 5% CO_2 and passaged when reaching >80% confluence. HDFs were purchased from PromoCell (C-12300), where authentication has been performed. **Urine Cells (UCs)** were obtained from healthy donors (ethical approval from the ethics committee of the medical faculty of the University of Heidelberg, study number S-550/2019) as described before (Mulder et al., 2020).

hESCs and hiPSCs, were grown on mitotically inactivated MEFs (passage 4), using Mitomycin-C at a final concentration of 10 ug/ml in complete DMEM, and 0.1% gelatin-coated plates. They were cultured in hESC media containing DMEM/F-12 + Glutamax (Gibco) supplemented with 20% KnockOut Serum Replacement (Gibco), 1% Pen/Strep (Gibco), 1% NEAA (Gibco) and 0.1mM β-mercaptoethanol (Gibco). A final concentration of 10-30 ng/µl of FGF2 (Peprotech) was added freshly. The media was replaced every second day and the cells were passaged by manual excision once a week. hiPSCs were derived from HDFs (3-year old male) or urine cells (male and female) by electroporation of pCXLE EBNA reprogramming vectors using feeder dependent or feeder-free reprogramming. hESCs were male.
For genetic modification at the SC stage, hiPSCs were transferred to a feeder-free culture system using iMatrix Laminin-511 (Amsbio) as coating reagent and Stemfit Basic02 media (Ajinomoto) supplemented with 10-30 ng/ul FGF2 (Peprotech). There, cells were passaged in clumps using ReLesR (Stem Cell Technologies) or as single cells using TrypLE (Gibco) or ProStem Accutase (Gibco) and media was changed three times per week.

Murine cells

MEFs and **lung fibroblasts** were cultured on gelatin-coated dishes (0.1% gelatin in distilled water) and grown in DMEM (with 4500 mg/L glucose, L-glutamine, and sodium bicarbonate, without sodium pyruvate), supplemented with 10% FBS (Gibco) and 1% Pen/Strep (Gibco). The cells were cultured in a humidified atmosphere at 37˚C and 5% CO_2. The cells were passaged on demand. CF1-MEFs (male, passage 3) were purchased from Merck. Primary lung fibroblasts were obtained from female C57BL/6N mice (Charles River, Sulzfeld, Germany) and generated as described before (Willemsen et al., 2017).
Immortalized Mouse Embryonic Fibroblasts (iMEFs) were used as feeder layers for co-culture of murine stem cells and reprogramming of human fibroblast. The cells were cultured as described above and grown until 90% confluency. Then the cells were washed, trypsinized and mitotically inactivated upon exposure to 60 Gy of γ-irradiation (Gamma cell 1000).
Mitotically inactivated MEFs (reference, Passage 4) using Mitomycin-C at a final concentration of 10 ug/ml in complete DMEM were used as feeder cells and co-cultured with human stem cells.
E14 129Ola mESC and miPSC were grown on feeder layers (on 0.1% gelatin-coated plates) and in KnockOut DMEM (Gibco) supplemented with 15% FCS superior (Biochrom), 1% Pen/Strep (Gibco), 1% NEAA (Gibco), 1% L-glutamine (Gibco), 0,1 mM β-mercaptoethanol (Gibco) and 1000 U/ml of Leukemia Inhibitory Factor (Merck). The differentiation inhibitors CHIR99021 and PD0325901 (Sigma) were added fresh to the media before use at a final concentration of 3 µM and 1 µM, respectively. The cells were cultured in a humidified atmosphere at 37˚C and 5% CO_2. The media was changed every second day and the cells were passaged twice a week. In order to separate mESCs from feeder layers, an intermediate differential sedimentation step was performed for 20 minutes at 37˚C and 5% CO_2 in IMDM media (Gibco) supplemented with 10% FCS (Gibco) and 1% Pen/Strep (Gibco). If needed, the cells were adapted into a feeder-free culturing system. mESC (E14 129Ola strain, male), miPSC were derived from CF1-MEF (Merck, male) or lung fibroblasts (female) by transduction with pWPI-4in1 lentiviral particles.

Lentiviral reprogramming

MEFs at a low passage (p3 - 5) were seeded (25.000 - 50.000 cells) per well of gelatinized 24 well plate. Transduction was performed in three rounds. The next morning (day 1), the media was aspirated, and the cells were infected with 200µl of viral particles suspended in DMEM media. The infection was repeated on the evening of the same day (day 1) and the following morning (day 2). Finally, the viral supernatant was aspirated, and without washing steps, the cells were fed with standard DMEM supplemented with 10% FCS and 1% Pen/Strep.

DNA Vector reprogramming

HDFs were dissociated with trypsin-EDTA (sigma) and 500.000 cells were transfected with either 2 µg of each episomal EBNA reprogramming plasmid (pCXLE-hUL, pCXLE-hSK, pCXLE-hOCT3/4-shp53-F, and pCXWB-EBNA1; Addgene) and if indicated, co-transfected together with labeling GFP vectors (pSMAR or nSMAR). Alternatively, HDFs were transfected with 2-3µg of SMAR reprogramming vectors (pPOP or nPOP) and co-transfected with 2 µg of the GFP-SMAR labeling vector pSMARt-shP53. The plasmid DNA was diluted in electroporation buffer (Lonza) and the cells were electroporated using the program P-022 and the Amaxa II Nucleofector device (Lonza) following the manufacturer's instructions. Following transfection, the cells were seeded on a six-well plate containing DMEM supplemented with 10% FCS (Gibco), 1% NEAA (Gibco) without PenStrep (Gibco), to allow the cells to recover. The PenStrep was added 24h after transfection and the media was changed every second day. At 8 days after transfection, 30.000 cells were seeded per well in triplicates in a six-well plate coated with 0.1% gelatin and containing feeder cells in hiPSC media containing DMEM/F12 with Glutamax supplement (Gibco), 20% Knockout Serum replacement (Gibco), 0.1 mM β-mercaptoethanol (Gibco), 1% Pen/Strep (Gibco), 1% NEAA (Gibco) and 10 ng/ml FGF2 (Peprotech). The hiPSC media was replaced every second day until hiPSC colonies emerged. Every experiment was repeated at least twice.
UCs were reprogrammed by transfection of episomal EBNA vectors as described elsewhere (Mulder et al., 2020) and transferred to feeder-free hiPSC conditions after 4 days and kept in these conditions.

Random differentiation

Murine random differentiation was achieved through the formation of EBs. For that, mESCs were passaged as described above and diluted in EB medium, containing KnockOut DMEM (Gibco), 15% KnockOut Serum Replacement (Gibco), 1% L-Glutamine (Gibco), 1% NEAA (Gibco), 1% Pen/Strep (Gibco) and 50 µg/ml Ascorbic acid (Sigma), devoid of LIF and the 2i inhibitors, to a density of $3x10^4$ cells/ml (or 600 cells per 20 µl). With the help of a multichannel pipette, 80-100 drops of 20 µl were placed on the lid of a bacterial petri dish, and the dish was filled with PBS to preserve the humidity. The drops were incubated at 37˚C and 5%CO_2 for 3 days. During this time, the cells collapsed in the bottom of the drop and formed undifferentiated aggregates. After 3 days, the drops from one lid were harvested and pooled with 4 ml of EB media, and the aggregates were cultured in suspension in EB media for additional 3 days. Then, the aggregates were transferred into a 15 ml conical tube with EB media and were sedimented at room temperature for 10 minutes. They were resuspended in fresh EB media and 1-2 aggregates were transferred per well of a gelatin-coated µ-plate 96 well (iBidi). The plate was incubated for 1-2 weeks and the EB media was

replaced every second day. The embryonic Bodies were checked regularly for differentiation and when obvious differentiated structures formed (*e.g.:* beating cardiomyocytes, neurons…), they were fixed and stained with antibodies for markers of the three germ layers (β3-TUBULIN, αSMA and FOXA2).
Human tri-germ layer differentiation was achieved using the StemMACS trilineage differentiation kit (Miiltenyi Biotec) following the manufacturer's recommendations. Briefly, feeder-free maintained hiPSCs were detached as single cells using StemPro Accutase (Gibco) and plated in the described respective density in stem cell media containing 10 µM ROCK inhibitor. The cells were cultivated for 7 days with the respective germ layer induction media and successful induction was verified via immunofluorescence staining. For quantification of GFP expression after 7 days of differentiation, GFP+ population was determined in single, alive cells using fluorescence cytometry and compared to the respective hiPSC lines maintained without differentiation.

Hematopoietic differentiation

mESCs were grown to confluency, washed, trypsinized and separated from feeder cells. Then, 75.000 cells were counted and plated in Ultra low attachment T25 flasks (Corning) containing EB-HSC differentiation medium (IMDM, 15% EB FCS (PAA Laboratories), 1% Pen/Strep (Gibco), 1% L-Glutamine (Gibco), 50 µg/ml Ascorbic acid, 4.5 mM monothioglycerol (Sigma) and 200 µg/ml Holotransferin (Sigma)), and were incubated for 2.5 days (60h) under hypoxic conditions (5% CO_2 and 5% O_2). After 60h, 5 ng/ml of each cytokine BMP-4 (R&D), Activin A (R&D), VEGF (R&D) and FGF2 (R&D) were added to the media and the cells were further incubated for 60h in hypoxia. At day 5, the EB were allowed to settle at the bottom of the flask. Then, the majority of the media containing cytokines was removed and centrifuged to pellet cell debris. Fresh EB media without cytokines was added to the embryonic bodies, and the conditioned media was added back to the flask, which was then incubated for another 24h in hypoxic conditions.
At day 6, the EBs were collected, washed and dissociated in 250 µl Enyzme mix containing 10 mg/ml Collagenase, 0.02 g/ml Hyaluronidase and 80 Units/ml DNaseI in 1 ml of PBS and incubated at 37˚C for 20 min with occasional swirling of the tube. Another 8 ml of enzyme-free dissociation media were added to the cells, which were then incubated for five minutes at room temperature. Finally, the cells were mechanically dissociated and washed with PBS, collected by centrifugation (1500 rpm for five min at 4˚C) and used for downstream analysis such as FACS.
The hematopoietic differentiation panel of antibodies used, included: CD41 (1:300), CD144/VE-Cadherin (1:400), CD117/c-Kit (1:2000) and 7-AAD (1:200) as life/death staining.

Bacterial strains

Bacterial competent cells DH5α (Life Technologies), DH10β (Life Technologies), Stbl3 (Life Technologies), and Stellar competent cells (Clontech), were grown in Luria-Bertani (LB) media or LB-agar plates with the corresponding antibiotic (Ampicillin 100 µg/ml; Carbenicillin 50 µg/ml; Kanamycin 30 µg/ml) at 37˚C.

Viral vectors

Lentiviral particles were generated in the laboratory of Dr. Marco Binder (DKFZ, Germany). Briefly, HEK293T cells were infected with viral vectors derived from the plasmid pWPI-BLR. The lentiviral particles were produced as described elsewhere (Koutsoudakis et al., 2006) by calcium phosphate transfection of the three individual plasmids at a 3:1:3 ratio: (i) pCMV-

ΔR8.91, coding for HIV Gag-Pol; (ii) pMD.2G, encoding the VSV-G glycoprotein; and (iii) the lentiviral vector pRRL.PPT.SF.hOKSM-IRES-dTOM or empty pWPI-Puro. (pCMV-ΔR8.91 and pMD.2G) were kind gifts from Didier Trono, Lausanne (Zufferey et al., 1997). Finally, the supernatants of HEK293T cells were collected, filtered, and frozen for further use, without viral particle titration.

Genotyping

DNA from tail biopsies or ear punches was extracted with the Phire Tissue Direct PCR Master Mix (Thermo Fischer) according to the manufacturer's instructions, in a total volume of 20 µl per reaction. Degenerate primers amplifying a non-coding mammalian genomic DNA region of *SOX21* were provided with the kit and used as internal controls (Primers G11-12). GFP primers for amplification of *coGFP* (515bp) or *eGFP* (237bp) were designed using Primer3 and validated *in silico (*Primers G7 - G8 for *coGFP* and G9 -G10 for *eGFP).* The PCR reactions for GFP and the internal controls were performed separately to get better amplification of GFP bands.

Alkaline Phosphatase (AP) staining

AP stainings were performed in black µ-Plate 96 Well (Ibidi) coated with gelatin or iMatrix Laminin-511 or in regular coated 24-well plates. The staining was performed using Alkaline Phosphatase Staining Kit II (Stemgent) as of manufacturer's instructions. The images were taken with a Keyence microscope (Keyence).

FACS

Flow Cytometry analysis was performed on a LSR Fortessa (BD Biosciences) and the data acquisition was done in a BD CellQuest Pro^{TM} or BD FACSDiva^{TM} software (BD Biosciences). Briefly, cells were washed, detached using trypsin or accutase and resuspended in 200ul PBS. Propidium Iodide (Life technologies) or 7-AAD (Invitrogen) were used as life-death discrimination and added to the cells shortly before FACS (PI, 1:1000; 7-AAD, 1:200). Data analysis was done with the software FlowJo. For transfected cells, no staining was needed as endogenous GFP expression could be detected. EB staining: CD41-PE-Cy7 (eBioscience, 1:300), CD144/VE-Cadherin-PE (BD Pharmigen, 1:400), CD117/c-Kit-Alexa 780 (eBioscience, 1:2000) and 7-AAD (1:200) as life/death staining. Blood Staining: CD45.1-APC-Cy7 (eBioscience, 1:300), CD11b - APC (eBioscience, 1:2000, B220-APC (eBioscience, 1:500), CD4-PE-Cy7 (eBioscience, 1:2000), and CD8a-PE-Cy7 (eBioscience, 1:3000), antibodies were prepared in 2% FCS/PBS. 7-AAD/2% FCS/PBS (5µl/ml, 1:200 dilution). Spleen staining: spleen was smashed through a filter (40 µm EASYstrainer, Greiner bio-one) and collected in 2% FCS/PBS. The cells were then stained with CD45-PB (eBioscience, 1:1000). Bone marrow: bones were cleaned from muscle, and the bone marrow was flushed out in 2 ml 2% FCS/PBS with the help of a syringe. The bone marrow cells were then stained with CD45-PB (eBioscience, 1:1000).

Plasmid rescue

500 - 1000ng of genomic DNA extracted from stable cells were used for transformation into DH10β *E.coli* competent cells (Life Technologies) in a Bio-Rad Gene Pulser using a standard pulse for bacteria. Transformed colonies were selected on agar plates containing the appropriate antibiotic. DNA was isolated from individual resistant clones, subjected to restriction analysis with the appropriate enzymes, and analyzed by gel electrophoresis on 1% agarose gels.

Southern Blot

Genomic DNA was extracted from cells using the DNeasy blood and tissue kit (Qiagen) following the manufacturers' instructions and was digested with a DNA vector single-cutter (usually *BamHI or AgeI*) for at least 4h at 37˚C. Additionally, 2 – 5 ng of parental plasmids were digested as positive controls. The digested genomic and plasmid DNA fragments were separated on a 0.8% agarose gel overnight and transferred into a Hybond-XL nylon membrane (Amersham Biosciences). DNA probes used for Southern blot analysis were radioactively labeled with [32P]dATP (3000 Ci/mmol) or [32P]dCTP (3000 Ci/mmol) using Prime-it II Random Primer Labeling Kit (Agilent) and following the manufacturer's instructions. 100 µl of radioactively labeled DNA probe was denatured, diluted in 1 ml of Church buffer and hybridized overnight at 65˚C.

Western Blot

Between $5x10^5$ and $1x10^6$ cells were lysed in 40 µl RIPA lysis buffer containing 2% SDS, 10 mM Tris pH 7.5 and 0.1 mg/ml protease inhibitor (Roche) and centrifuged at 14000 g for 30 min at 4˚ C. The protein concentration of the cell lysate was determined using the Pierce™ BCA Protein Assay Kit (Thermo Fischer) and equal amounts (30 µg) were separated on a 4-12% SDS-PAGE gel (Bio-Rad) with a protein marker PageRuler Plus Prestained Protein Ladder (Fermentas). Following separation, total protein was transferred into PVDF iBlot 2 Transfer Stacks (Invitrogen) using an iBlot2 device (Invitrogen). The membrane was blocked in 5% non-fat milk in TBS-Tween20, and incubated with primary antibodies at the appropriate dilution, overnight at 4˚C. For detection, secondary antibodies conjugated to HRP were incubated with the membrane at the appropriate dilution for 1h at room temperature. The membrane was developed using SignalFire™ ECL Reagent (Cell Signaling Technology) by enhanced chemiluminescent detection system by FusionSL Vilber Lourmat system.

Pluripotency Western analysis was performed using: primary rabbit polyclonal KLF4 antibody (Santa Cruz, 1:500 dilution), goat polyclonal OCT3/4 (Santa Cruz, 1:500), rabbit polyclonal SOX2 (Merck, 1:1000), mouse monoclonal C-MYC (Santa Cruz, 1:150); mouse monoclonal LIN28 (Santa Cruz, 1:200), mouse monoclonal α-TUBULIN (Thermo Fischer, 1:10.000) or mouse monoclonal GAPDH (Santa Cruz, 1:500) as loading controls; and the secondary antibodies anti-mouse-HRP, anti-rabbit-HRP and anti-goat-HRP (Life Technolgies,1:10.000). For FancA western blots: the primary antibody rabbit polyclonal FANCA (Merck, 1:5000), mouse monoclonal GAPDH (Santa Cruz, 1:500) and the secondary antibodies anti-mouse-HRP, anti-rabbit-HRP and anti-goat-HRP (Life Technolgies, 1:10.000).

qRT-PCR

For qRT-PCR, approximately half a million cells were harvested and lysed in 500ul trizol. RNA was isolated as of manufacturers recommendation by addition of 100ul chloroform, separation of aqueous phase and precipitation using isopropanol. RNA was digested with DNase I using the DNA-free DNA removal kit (Invitrogen). Subsequent cDNA synthesis was performed using the RevertAid H minus first-strand cDNA synthesis kit (Invitrogen) and a mixture of oligo dT and random primers. For qRT-PCR, cDNA was diluted to 5ng/ul and 2ul was combined with a 23ul master mix composed of 12.5ul primaQuant cybr blue 2x qPCR sybrgreen master mix (Steinbrenner, SL-9902B), 8.5ul water, and 1ul of forward and reverse primer. (5uM) The reaction was run on a LightCycler96 instrument (Roche).
