# Supplementary material for: An episomal DNA vector platform for the persistent genetic modification of pluripotent stem cells and their differentiated progeny
Source: Stem Cell Reports. 2021 Dec 22;17(1):143–58. doi: 10.1016/j.stemcr.2021.11.011 (PMC8758943; doi:10.1016/j.stemcr.2021.11.011)
Supplement: Document S2. Article plus supplemental information [file mmc4.pdf]

Stem Cell Reports
Article
OPEN ACCESS

An episomal DNA vector platform for the persistent genetic modification of pluripotent stem cells and their differentiated progeny

Alicia Roig-Merino,[1,8] Manuela Urban,[1,8] Matthias Bozza,[1] Julia D. Peterson,[1] Louise Bullen,[2] Marleen Büchler-Schäff,[3,4] Sina Stäble,[3,5] Franciscus van der Hoeven,[6] Karin Müller-Decker,[7] Tristan R. McKay,[2] Michael D. Milsom,[3,4] and Richard P. Harbottle[1,9,*]
[1]DNA Vectors, German Cancer Research Center (DKFZ), Heidelberg 69120, Germany
[2]Stem Cell Biology, Manchester Metropolitan University (MMU), Manchester M1 5GD, UK
[3]Heidelberg Institute for Stem Cell Technology and Experimental Medicine (Hi-STEM), Heidelberg 69120, Germany
[4]Division of Experimental Hematology, DKFZ, Heidelberg 69120, Germany
[5]Translational Cancer Epigenomics, Division of Translational Medical Oncology, DKFZ, Heidelberg 69120, Germany
[6]Transgenics Service, DKFZ, Heidelberg 69120, Germany
[7]Tumor Models, DKFZ, Heidelberg 69120, Germany
[8]These authors contributed equally
[9]Lead contact
*Correspondence: r.harbottle@dkfz.de
https://doi.org/10.1016/j.stemcr.2021.11.011

SUMMARY

The genetic modification of stem cells (SCs) is typically achieved using integrating vectors, whose potential integrative genotoxicity and propensity for epigenetic silencing during differentiation limit their application. The genetic modification of cells should provide sustainable levels of transgene expression, without compromising the viability of a cell or its progeny. We developed nonviral, nonintegrating, and autonomously replicating minimally sized DNA nanovectors to persistently genetically modify SCs and their differentiated progeny without causing any molecular or genetic damage. These DNA vectors are capable of efficiently modifying murine and human pluripotent SCs with minimal impact and without differentiation-mediated transgene silencing or vector loss. We demonstrate that these vectors remain episomal and provide robust and sustained transgene expression during self-renewal and targeted differentiation of SCs both *in vitro* and *in vivo* through embryogenesis and differentiation into adult tissues, without damaging their phenotypic characteristics.

INTRODUCTION

Pluripotent stem cells (PSCs) are an invaluable source of cells for regenerative therapies due to their capacity for proliferation, self-renewal, and their potential for multi-lineage differentiation (He et al., 2009; Schwanke et al., 2014). Induced PSCs (iPSCs) can be derived from somatic cells (Takahashi and Yamanaka, 2006) and isolated using minimally invasive techniques. This not only limits concerns regarding the use of embryonic SCs (ESCs) but the risk of immune rejection as an autologous therapy. Therefore, iPSCs are an attractive tool for personalized medicine, drug screening, and the generation of disease models (Takahashi and Yamanaka, 2013).

SCs are notoriously difficult to modify genetically; they are typically refractory to transfection, their extensive proliferation leads to vector dilution, and the dramatic changes in the cellular milieu following differentiation can lead to transgene silencing.

A variety of methods are used to persistently genetically modify and derive SCs (Table 1), but most rely on integrating lentiviral vectors. Despite advances, low transduction efficiency and silencing are still observed using retroviruses in hematopoietic (HSCs) and mesenchymal SCs (MSCs) (Zhang et al., 2002). Additionally, problems associated with random insertion into untranscribed regulatory regions (5′UTR) and consequent dysregulation of neighboring genes (Cattoglio et al., 2007) (Kotterman et al., 2015) affect the use of lentiviruses in SCs (Herbst et al., 2012).

Vectors such as transposons can be used to genetically engineer PSCs (Park et al., 2018; Querques et al., 2019), while sustaining transgene expression during differentiation (Chen et al., 2009; Orbán et al., 2009; Wilber et al., 2007). However, they can randomly integrate, potentially interfering with the cells' integrity. They can also be engineered using sequence-specific nucleases (Czerwińska et al., 2019; Song and Ramakrishna, 2018). However, despite intensive research, undesired off-target effects and editing efficiency remain an issue requiring thorough screening and genomic characterization (Kim et al., 2017). SC engineering and iPSC derivation can also be achieved using episomal plasmids, which predominantly comprise viral components such as Epstein-Barr virus Nuclear Antigen 1 *(EBNA-1)* (Sugden et al., 1985; Thyagarajan et al., 2009; Yates et al., 1985) or the large T antigen from Simian Virus 40 *(SV40)*. EBNA-based systems rely on the oncoprotein EBNA-1 (Humme et al., 2003), which interacts

This is an open access article under the CC BY license (http://creativecommons.org/licenses/by/4.0/).

Table 1. Overview of gene therapy vectors

	γ-retrovirus	EBNA vectors	Transposons	Minicircles	SMAR minicircles	SMAR plasmids (pSMAR)	SMAR nanovectors (nSMAR)
**Capacity**	Medium	High	High	High	High	High	High
**Maintenance**	Yes	Yes	Yes	No	Yes	Yes	Yes
**Integrative**	Yes	Possibly	Yes	No	No	No	No
**Replicative**	Yes	Yes	Yes	No	Yes	Yes	Yes
**Oncogenic**	High	High	Medium	Low	Low	Low	Low
**Manufacturing**	Difficult	Easy	Easy	Difficult	Difficult	Easy	Easy
**Immunogenic**	High	High	Medium	Medium	Medium	Medium	Low
**Bacterial antibiotic free**	No	No	Yes	Yes	Yes	No	Yes

with the *MYC* promoter (Canaan et al., 2009; Sung and Pagano, 1995), and can dysregulate genes associated with cell growth, rendering them potentially oncogenic (Canaan et al., 2009). Thus, using an episomal vector devoid of viral elements for its maintenance is highly desirable.

The plasmid pEPI can function as an episome using genetic elements known as scaffold/matrix attachment regions (SMARs) (Piechaczek et al., 1999). SMARs interact with transcription factories, influencing gene expression by controlling the recruitment of transcription factors, chromatin structure, and accessibility (Hagedorn et al., 2013). In a plasmid, SMARs facilitate episomal replication and maintenance (Stehle et al., 2003) in various cells (Hagedorn et al., 2012), including human HSC (Papapetrou et al., 2006), and prevent epigenetic silencing, while enhancing transgene expression (Piechaczek et al., 1999). Upon delivery, vector molecules reach the nucleus and are stochastically established depending on their proximity to nuclear compartments (Hagedorn et al., 2017; Stehle et al., 2007). Vectors are episomally maintained at low copy numbers (Stehle et al., 2007), are stable in the absence of selection (Piechaczek et al., 1999), are co-segregated with chromosomes during mitosis, and have unlimited cloning capacity (Lufino et al., 2007).

SMAR vectors have been systematically modified to improve their application (Hagedorn and Lipps, 2013) by swapping the original promoter for *in vivo* applications (Manzini et al., 2010; Wong et al., 2011; Argyros et al., 2008), by reducing potential immunogenicity, by reducing or removing the vector backbone's CpG content (Haase et al., 2010), or by generating SMAR minicircles (Argyros et al., 2011). Minicircle production is inefficient, difficult, and costly, resulting in heterogeneous DNA. SMAR nanovectors based on an RNA-Out technology (Luke et al., 2009) are produced more simply with higher purity.

Here, we describe a nonviral, nonintegrating, and autonomously replicating SMAR vectors that can be used to persistently engineer SCs without causing molecular or genetic damage, while providing sustained transgene expression during differentiation and reprogramming. Within this study, we generated two novel vectors, pSMAR and nSMAR, by refining their composition and functional elements. We compared our new vectors' behavior to the original pEPI vector and evaluated their episomal replication, establishment efficiency, long-term maintenance, and transgene expression. Both newly designed vectors outperform the originals by every measure.

RESULTS

pSMAR and nSMAR generate highly expressing stable SC lines while remaining episomal

Refined SMAR vectors are based on pEPI-CMV-UCOE (Hagedorn et al., 2013) (Figure 1A). The SMAR element was retained and the CMV promoter replaced with the CAG (Fregien and Davidson, 1986; Miyazaki et al., 1989) to provide robust transgene expression (pEPI-CAG), their composition reorganized by directly coupling the selection marker to the expression cassette and SMAR motif (pSMAR) (Bozza et al., 2020). We generated minimally sized nanovectors (nSMAR) by eliminating bacterial sequences and reducing the backbone to 431 bp, a reduction of 17.41%. Each vector encoded the reporter gene *GFP* (Figure 1B) and was directly compared to determine efficiency, stability, and durability of expression.

Electroporation of mouse ESCs (mESCs) with pEPI-CMV-UCOE resulted in transfection efficiency of 25.8% ± 2.2% compared with slightly increased efficiency of 31.8% ± 5.5% with pEPI-CAG. Transfection efficiency and

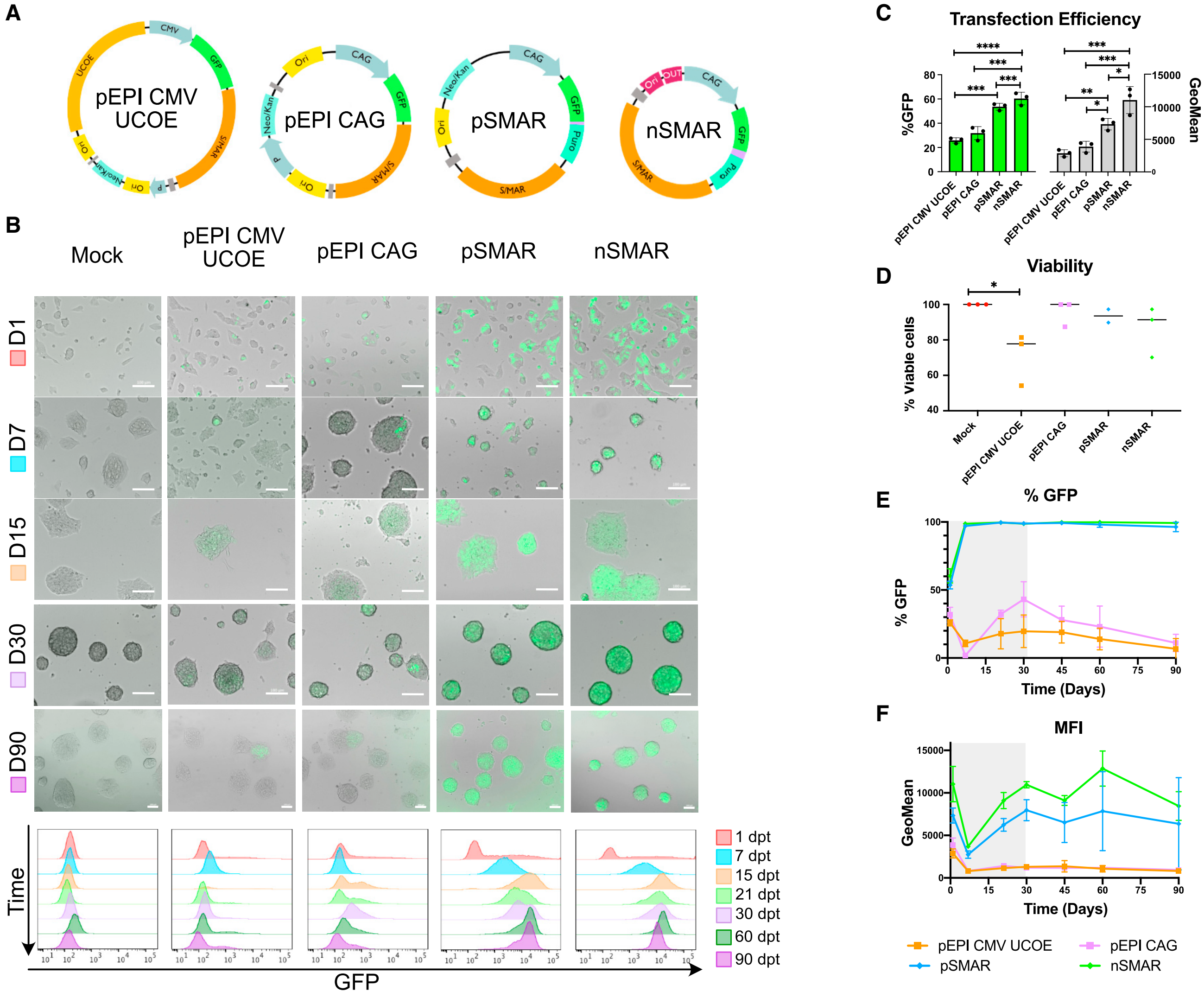

Figure 1. Increased vector performance is reflected by improved vector design

(A) Schematics of DNA vectors used in this study.

(B) Monitoring and quantification of GFP expression in mESC by microscopy and FACS analysis. GFP fluorescence gated on the alive population. Images and histograms from three (n = 3) independent experiments (scale bars = 100 μm).

(C) Transfection efficiency (24 hpt) of transfected mESC. Results expressed as mean ± SD of %GFP + cells and MFI (GeoMean) from three (n = 3) independent experiments. Statistical analysis was performed as follows: GFP and MFI: Shapiro-Wilk normality test passed, 1-way ANOVA with Tukey's multiple comparison test. GFP: pEPI-CMV-UCOE versus pEPI-CAG, p-val = ns; pEPI-CMV-UCOE versus pSMAR, ***p = 00.002; pEPI-CMV-UCOE versus nSMAR, ****p < 00.001; pEPI-CAG versus pSMAR, ***p = 00.009; pEPI-CAG versus nSMAR, ***p = 00.001; pSMAR versus nSMAR, p = ns). MFI: pEPI-CMV-UCOE versus pEPI-CAG, p = ns; pEPI-CMV-UCOE versus pSMAR, **p = 00.094; pEPI-CMV-UCOE versus nSMAR, ***p = 00.002; pEPI-CAG versus pSMAR, *p = 00.361; pEPI-CAG versus nSMAR, ***p = 00.005; pSMAR versus nSMAR, *p = 00.255).

(D) Cell viability of transfected mESC at 24 hpt from three (n = 3) independent experiments. The results are expressed as % alive transfected cells normalized to alive mock cells transfected without DNA. Statistical analysis was performed using Shapiro-Wilk normality test and 1-way ANOVA with Dunnett's multiple comparison test (*p = 0.0258).

(E) Plot showing %GFP + cells over 3 months. The grayed-out area corresponds to the antibiotic selection period. The results are expressed as mean ± SD from three (n = 3) independent experiments.

(F) Plot showing the MFI (GeoMean) variation over time. The grayed-out area corresponds to the antibiotic selection period. The results are expressed as mean ± SD from three (n = 3) independent experiments.

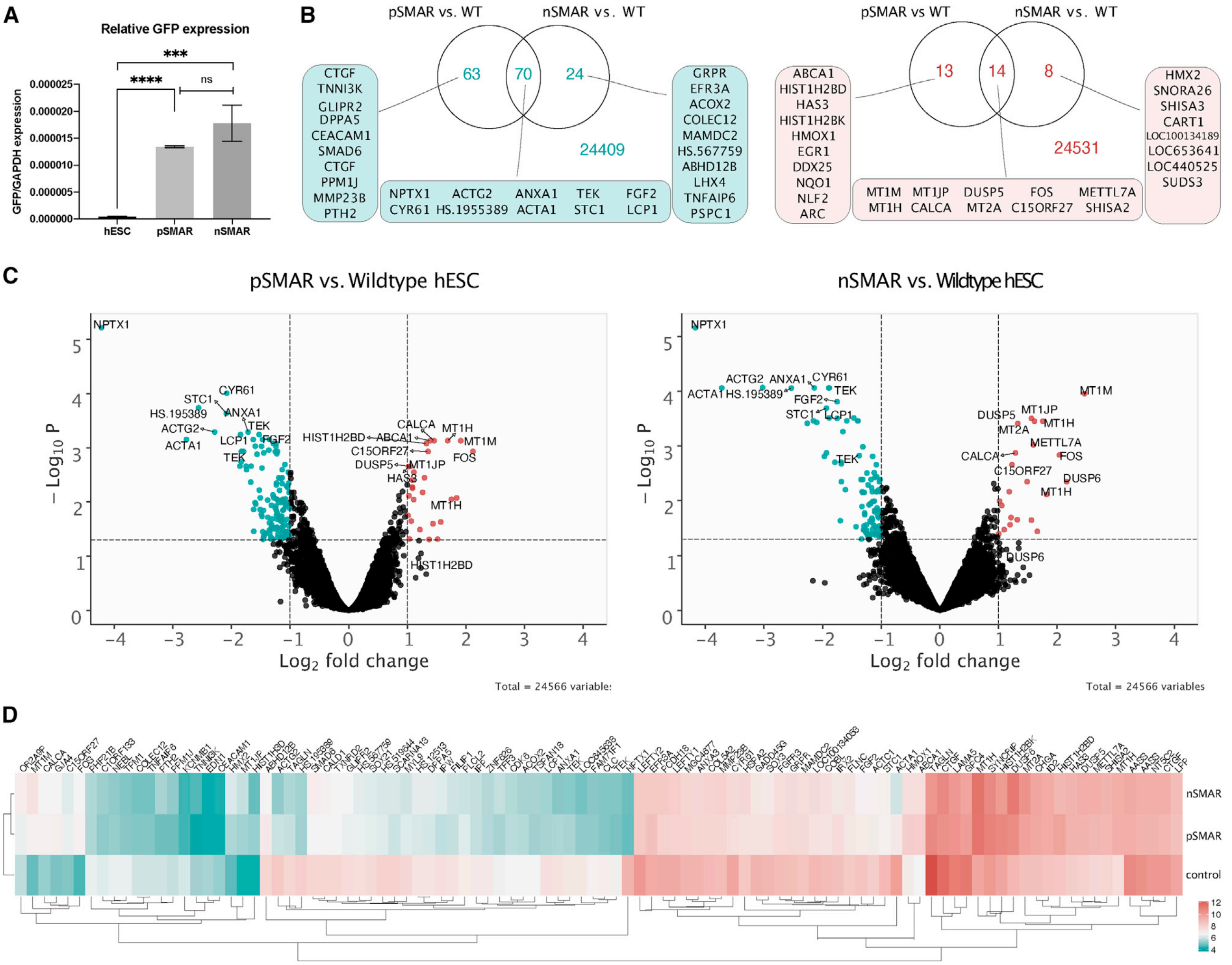

E

Group	Reactome ID	Reactome Pathway name	Entities	p-val	FDR
**Downregulated common**	R-HSA-445355	Smooth Muscle Contraction	8/55	6,16E-11	1,34E-08
	R-HSA-397014	Muscle contraction	10/224	1,78E-08	1,94E-06
	R-HSA-1839130	Signaling by activated point mutants of FGFR3	4/17	7,18E-07	5,17E-05
**Upregulated common**	R-HSA-5661231	Metallothioneins bind metals	4/16	4,22E-09	5,65E-07
	R-HSA-5660526	Response to metal ions	4/21	1,25E-08	8,36E-07
	R-HSA-419812	Calcitonin-like ligand receptors	3/11	3,38E-07	1,49E-05
**Upregulated pSMAR**	R-HSA-9031628	NGF-stimulated transcription	4/56	1,45E-06	2,15E-04
	R-HSA-198725	Nuclear Events (kinase and TF activation)	4/80	5,90E-06	4,37E-04
	R-HSA-9648895	Response of EIF2AK1 (HRI) to heme deficiency	3/29	1,14E-05	5,61E-04

Figure 2. SMAR nanovectors have minimal impact on cells' transcriptome

hESCs were electroporated with pSMAR and nSMAR. RNA was DNaseI-treated before microarray analysis (IlluminaHuman12 chip). RNA extractions from three different cell samples ($n = 3$) were used. Expression profiles were background corrected, quantile normalized, and $\log_2$ transformed using the Limma package from R. Linear modeling was performed, and the empirical Bayes method was used to assess differential expression.

(A) Comparison of transgene expression in transfected hESC. qPCR analysis of GFP normalized to GAPDH. One-way ANOVA ($p < 0.0001$) and unpaired 2-tailed T test (****$p < 0.0001$, ***$p = 0.0009$, ns = 0.0870).

(B) Venn Diagrams indicate the number of unique or similarly dysregulated genes between each pairwise comparison with adjusted $p < 0.05$ and FC > 2. The top ten differentially expressed genes within each category are listed.

(legend continued on next page)

fluorescence intensity dramatically increased when using pSMAR (53.6% ± 2.8%) and nSMAR (60.4% ± 5.2%) compared with pEPI vectors (Figure 1C). pEPI-CMV-UCOE resulted in the lowest transfection efficiency and reduced cell viability (71% ± 15%) (Figure 1D).

The vectors' ability to form stable cell lines was evaluated by following their expression for 30 days under selection and monitoring transgene maintenance >60 days in its absence. After 7 days, we observed a decrease in GFP + cells in pEPI-CMV-UCOE (10.7% ± 2.4%) and pEPI-CAG (1.8% ± 0.5%). However, GFP-neomycin-resistant colonies grew further. In contrast, pSMAR and nSMAR provided robust and stable transgene expression and GFP + cells could be observed throughout the experiment, even after >60 days with no selection (Figures 1E and 1F). Additionally, we validated the functionality of pSMAR and nSMAR in primary cells, such as murine embryonic fibroblasts (MEFs) (Figure S1A), murine iPSCs (miPSCs) (Figure S1B), and human iPSCs (hiPSCs) (Figures S1C and S1D). pEPI vectors could not generate stable GFP-hiPSCs (Figure S1C), and neomycin-resistant GFP clones remained growing. We obtained stable and highly expressing GFP-hiPSCs with pSMAR (98.5%) and nSMAR (99.6%), even after 3 months with no selection (Figure S1D). For translational applications, we generated pSMAR and nSMAR expressing hiPSC lines derived from urine iPSCs (UiPSCs) in the absence of selection. We observed stable and persistent GFP expression >170 days (Figure S1E).

Differences in vector performance were due to the vectors' composition not DNA purity (Figure S1F and S1G). We evaluated their integrity and stability by Southern blot from stably transfected mESCs, in which we observed two unique bands of 7,162 bp (pSMAR) and 5,915 bp (nSMAR) (Figure S1H). To further confirm the vectors' episomal status, we performed plasmid rescue, in which circular episomal forms could be retrieved in 90% of the cases (Figure S1I). We confirmed the rescued vector's integrity and sequence by restriction digestion and PCR amplification for both transgene and SMAR motif.

pSMAR and nSMAR vectors outperformed pEPI vectors in delivering high levels of stable transgene expression in rapidly proliferating cells while remaining episomal. Given the poor performance and rapid loss of transgene expression of pEPI-transfected cells, further experiments were only performed using pSMAR and nSMAR.

SMAR vectors show minimal impact on hESCs

To investigate the impact of SMAR vectors on SCs, hESC engineered with either pSMAR or nSMAR were subjected to microarray analysis. Their transcriptional profiles were compared with those of untransfected cells. We observed 160 and 116 differentially expressed genes, respectively. Sixty-three downregulated genes are unique to pSMAR modification, while only 24 are unique to nSMAR modification; 13 upregulated genes are unique to pSMAR modification, while only eight are unique to nSMAR modification (Figures 2B and 2C).

GFP levels were similar in pSMAR- and nSMAR-modified hESCs, suggesting that the differences found between the transcriptional profiles of the modified hESC are not attributed to the intensity of transgene expression (Figure 2A). Subsequent gene cluster analysis of the top 100 dysregulated genes indicates a closer relationship between pSMAR and nSMAR, while nonmodified hESCs have the furthest relationship from both (Figure 2D). We performed Reactome and GO TERM analysis on the unique and common dysregulated genes. Common downregulated genes are involved with muscle contraction and FGF3 signaling, while commonly upregulated genes are associated with metallothioneins and response to metal ions. Upregulated pSMAR-specific genes belong to NGF-stimulated transcription, kinase and transcription factor activation, and response to heme deficiency. We were unable to find statistically significant enriched gene sets for nSMAR-specific genes (Figure 2E). For detailed information refer to Figure S2 and Table S1.

These results suggest that SMAR vectors have a minimal impact on the host cell's endogenous transcription, and nSMAR causes the least disturbance to cells' molecular integrity, resulting in no significant dysregulation.

SMAR vectors can genetically modify murine and human primary fibroblasts and persist during reprogramming

We then evaluated the SMAR vectors' suitability, performance, and survival during reprogramming to iPSCs. Mouse lung fibroblasts were modified with pSMAR and selected to generate stable GFP-fibroblasts (Figure 3A). The episomal state of pSMAR was confirmed by plasmid rescue (Figure S3A). Then, pSMAR-fibroblasts were reprogrammed using pWPI-4in1, encoding the reprogramming factors *OKSM* and *dTOMATO* (Maetzig et al., 2014; Warlich

(C) Volcano plots display pairwise comparisons of expression profiles from pSMAR-hESC and nSMAR-hESCs versus wild-type cells. Adjusted $p < 0.05$ ($-\log_{10}P$ of 1.3) and an FC > 2 ($\log_2FC$ of 1). Green = downregulated and red = upregulated genes. The top ten differentially expressed genes are listed.
(D) Hierarchical clustering was performed using the average normalized expression values from the top 100 differentially expressed genes using Euclidean as a distancing measure and median as a clustering method for each group (n = 3).
(E) Reactome analysis was performed on the list of common or vector-specific dysregulated genes.

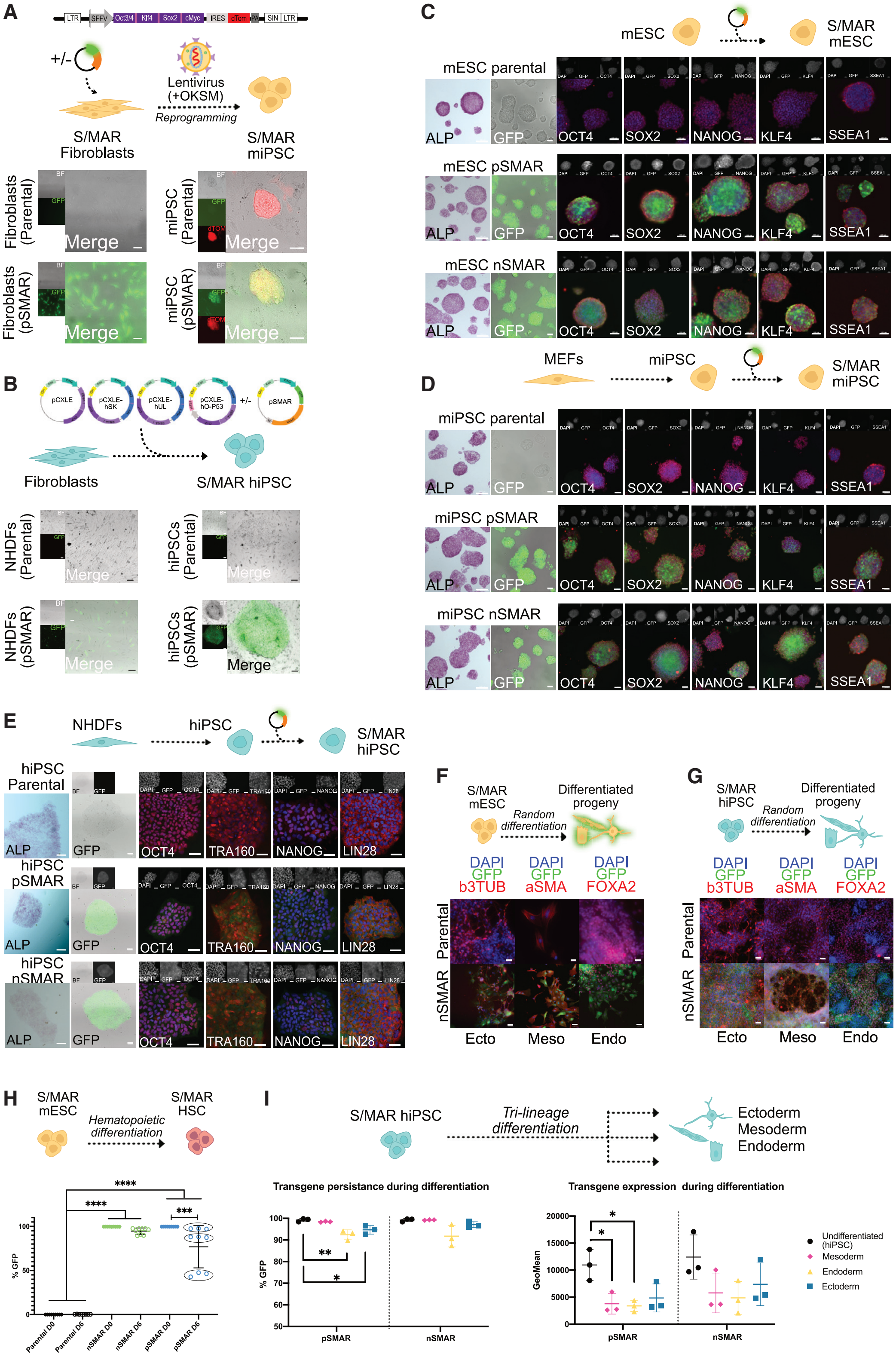

(legend on next page)

et al., 2011). After 14 days, morphologically distinct dome-shaped colonies emerged, expressing both GFP and dTOM, indicating the presence of SMAR vectors and the reprogramming lentivirus, respectively. GFP expression could be observed during reprogramming, proving the vectors' resistance to epigenetic silencing (Figure 3A).

In a translational approach, we validated the persistence of transgene expression in hiPSCs derived from dermal fibroblasts following co-transfection of pSMAR with the well-established EBNA-1/OriP reprogramming system (Okita et al., 2011). Morphologically distinct GFP-hiPSC colonies were obtained, indicating the presence and survival of pSMAR during reprogramming (Figure 3B).

SMAR vectors genetically modify murine and human cells without impairing pluripotency

We assessed if genetic modification of PSCs with SMAR vectors impacts pluripotency measuring the effect of SMAR vectors on the functionality and pluripotency of transfected mESCs using pSMAR and nSMAR. The cell lines were alkaline phosphatase (ALP)-positive and expressed all pluripotency markers in immunofluorescence (IF) stainings and Western blots (Figure S3B) and expression of GFP (Figure 3C).

To address the genetic modification of miPSCs, MEFs were reprogrammed using a pWPI-4in1 lentivirus, as described above, and miPSCs were electroporated with pSMAR and nSMAR (refer to Figure S1B). Pluripotency of modified miPSCs was confirmed via ALP and IF stainings for pluripotency markers (Figure 3D) and Western blot (Figure S3B).

Similarly, we modified hiPSCs derived from NHDFs with pSMAR and nSMAR. The pluripotency of the engineered hiPSCs remained intact, as cells were positive for all pluripotency markers tested (Figures 3E and S3C). We observe no differences in expression when we compared the pluripotency factors OCT4 and NANOG in similarly expressing pSMAR or nSMAR-hiPSCs and the parental hiPSC line (Figure S3D).

SMAR vectors survive *in vitro* random differentiation

Engineered mESCs were subjected to random differentiation; EBs were monitored and imaged regularly to check for GFP expression and hence the presence and function of the vectors during differentiation (Figure S3F). Independent experiments showed that stable pSMAR and nSMAR mESCs formed compact EBs and differentiated into structures such as neurons or contracting myocytes. Cells were fixed and stained for ectoderm (β3-TUBULIN), mesoderm (αSMA), and endoderm (FOXA2) markers while sustaining high levels of transgene expression throughout the differentiation process (Figures 3F, S3F, and S3G). Similar results were observed and confirmed in engineered miPSCs (Figures S3H and S3I).

Then, we addressed the survival of SMAR vectors in engineered hiPSCs through trilineage differentiation into ectoderm (β3-TUBULIN), mesoderm (αSMA), and endoderm (FOXA2). Successful differentiation and sustained levels of transgene expression through endogenous GFP expression were observed (Figure 3G).

Finally, we investigated if SMAR vectors survive sequential reprogramming followed by differentiation. We used miPSCs and hiPSCs derived from pSMAR-modified fibroblasts, which already maintained SMAR vector expression during reprogramming (Figures 3A and 3B). Similarly, we demonstrated that SMAR vectors provide sustained and high levels of transgene expression during differentiation

Figure 3. Maintenance of transgene expression through reprogramming and differentiation in miPSCs and hiPSCs

(A) Genetic modification of MEFs with pSMAR (GFP) and further reprogramming to miPSCs upon transduction with pWPI 4-in-1 lentiviral particles, expressing the reprogramming factors OKSM and dTOM (scale bars = 100 μm).

(B) Simultaneous labeling with pSMAR (GFP) and reprogramming of hiPSCs using EBNA-1 episomal vectors (scale bars = 100 μm).

(C) Immunofluorescence (IF) of pluripotency markers of parental, pSMAR, and nSMAR stable mESCs. Expression and localization of OCT4, NANOG, SSEA-1, Alkaline Phosphatase, and endogenous GFP (scale bars = 100 μm).

(D) IF of pluripotency markers of miPSCs generated from CF1-MEFs, genetically modified at the SC stage (scale bars = 100 μm).

(E) IF staining of hiPSC modified at the SC stage. Pluripotency markers (OCT4, LIN28, NANOG, TRA-160) and endogenous GFP (scale bars = 100 μm).

(F) IF staining of Mesoderm (αSMA), Ectoderm (β3TUB), and Endonderm (FOXA2) in randomly differentiated mESCs. Endogenous GFP was preserved (scale bars = 100 μm).

(G) IF staining of guided three-germ layer differentiation of parental and stable modified hiPSCs (scale bars = 100 μm).

(H) Hematopoietic differentiation of parental (passage 14, n = 3), pSMAR (passage 20, 14, 5; n = 3), and nSMAR mESC clones (passage 20, 14, 5; n = 3). The plot represents pooled biological replicates for the same vector. The GFP expression of each clone was analyzed using flow cytometry before (mESCs, day 0) and after (HSC, day 6) differentiation. The circles represent three technical replicates (n = 3) of the same clone. One-way ANOVA and Tukey's multiple comparison test were used for statistical analysis (****$p < 00.001$; ***$p = 00.002$).

(I) Quantification of transgene expression and persistence during differentiation. hiPSC modified with pSMAR (n = 3) or nSMAR (n = 3) were differentiated into Ectoderm, Mesoderm, or Endoderm. Transgene expression was measured by FACS in the differentiated lineages compared with their respective undifferentiated control (hiPSC). Two-tailed unpaired T tests were used for statistical analysis (**$p = 00.081$, *$p = 0.021$, *$p = 0.022$, *$p = 0.012$).

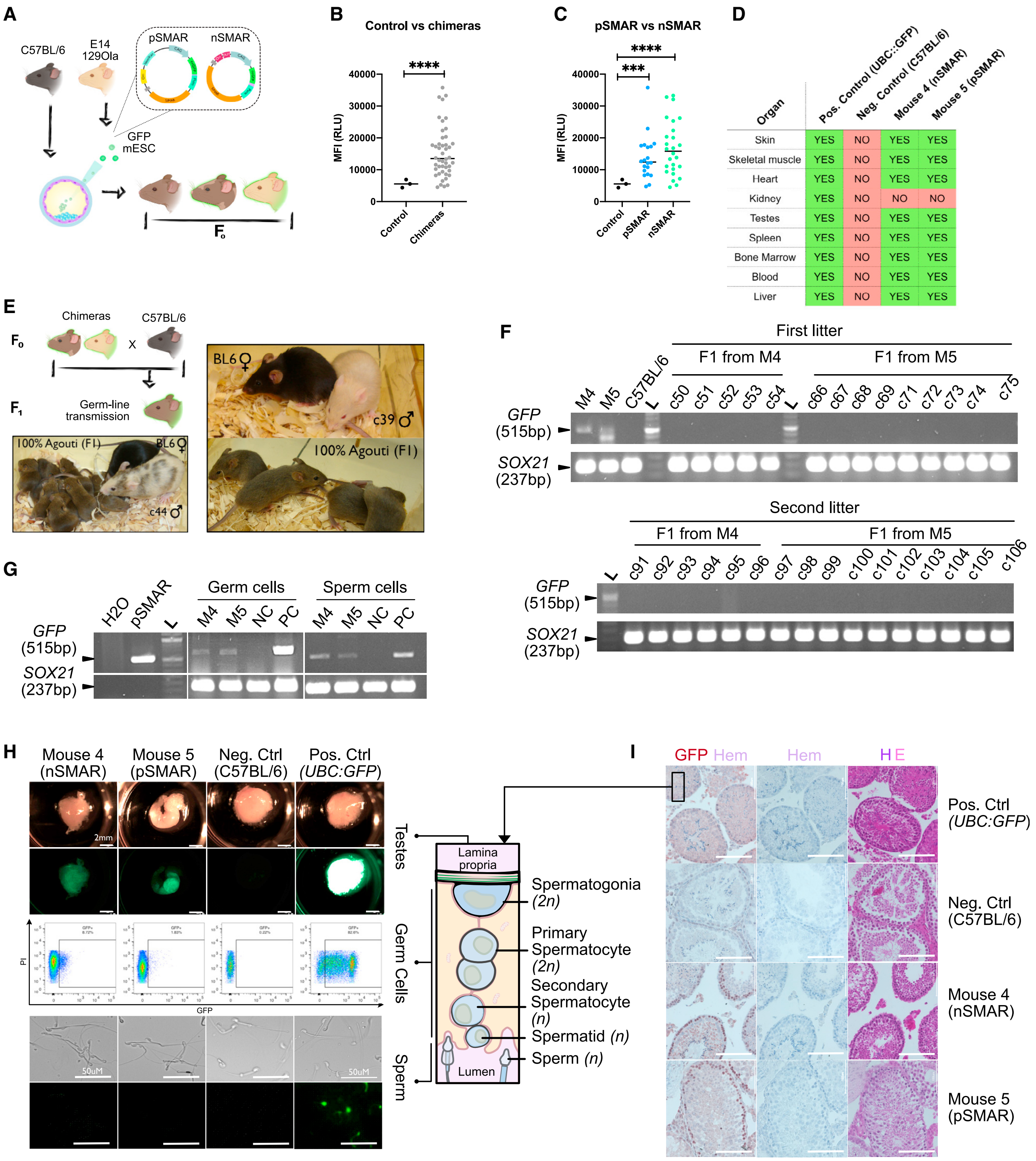

Figure 4. SMAR mESCs contribute to form chimeras (F0), but the genetic modification does not affect the progeny (F1)
(A) Chimeras generated with pSMAR (clone v71c22) or nSMAR (clone v85c17) mESCs. Between 6 and 12, stably transfected mESCs were microinjected into C57BL/6NxB6D2F1 embryos, resulting in the formation of chimeras, as observed by the agouti/chinchilla coat color.
(B) Transgenic GFP expression of ear biopsies (n = 49) at the time of weaning compared with C57BL/6N control mice (n = 3). The MFI from fluorescent images is expressed as relative light units (RLU). The statistical analysis was performed using an unpaired T test with Welch's correction (****p < 00.001).

(legend continued on next page)

in hiPSCs (Figures S3J and S3K) and miPSCs (Figures S3L and S3M), and genetically modified cells at the fibroblast level can also differentiate into representatives of the three germ layers. In summary, we not only showed that our modified episomal vectors do not compromise the functional potency of iPSCs, but also, they show an unprecedented resistance to epigenetic silencing.

SMAR vectors survive directed *in vitro* differentiation

After confirming the pluripotent capabilities of SMAR-engineered SCs and transgene maintenance during random differentiation, we sought to quantify the transgene expression of modified cells during directed differentiation.

First, mESCs engineered with pSMAR or nSMAR were subjected to hematopoietic differentiation. Three clones at different passages of stably transfected mESCs with pSMAR or nSMAR were forced to collapse into EBs under hypoxic conditions (5%O2). Unmodified mESCs were used as a control. After 6 days, successful differentiation was confirmed by the presence of a CD41 + cKIT + hematopoietic precursor population. GFP expression was quantified at the mESCs (day 0) and HSC (day 6) stage. Notably, a significant decrease in fluorescence was observed in pSMAR-labeled cells (99.76% to 76.90%), and this reduction correlated with the age of the clones, while nSMAR-labeled cells maintained GFP expression during the experiment (99.70% to 94.62%), in all clones (Figure 3H). Interestingly, the highest decrease in fluorescence corresponded with cells labeled with vectors containing bacterial sequences (pSMAR) and was less prominent when nanovectors were used (nSMAR).

Then, we quantified persistence (%GFP) and expression levels (MFI) of engineered hiPSCs during differentiation into the three germ layers. Endoderm, mesoderm, and ectoderm derivatives were analyzed and compared with undifferentiated cells (Figures 3I and S3N). In line with the murine hematopoietic differentiation, we observed a slight but significant decrease in the %GFP of pSMAR cells, particularly in the endoderm (−6.83% ± 2.75%) and ectoderm (−4.53% ± 1.30%). Additionally, we observed a slight decrease in the MFI of mesoderm and endoderm derivatives in pSMAR-hiPSCs. Notably, no significant decrease in persistence or expression was observed in nSMAR-hiPSCs.

Together, these data demonstrate the minimal impact of SMAR vectors on modified SCs, as the cells express all pluripotent markers tested and exhibit full differentiation potential. Additionally, SMAR vectors retained transgene expression during *in vitro* differentiation into derivatives of the three germ layers, as well as hematopoietic precursors.

SMAR vectors survive *in vivo* differentiation and generate chimeric mice

An emphatic evaluation of the vectors' mitotic stability and a more stringent measure of pluripotency was performed by assessing the SCs' ability to form chimeras when injected into early-stage embryos. GFP-mESC clones (chinchilla) generated with pSMAR or nSMAR were injected into *morulae* of C57BL/6N x B6D2F1 embryos. Forty-nine chimeric pups were born, in which the presence and contribution of engineered mESCs could be observed by the agouti/chinchilla coat chimerism over the black background (Figure 4A). All pups showed varying degrees of chimerism, reaching in some cases a 100% chinchilla coat color, suggesting that a high proportion of the chimera was contributed by the genetically modified mESCs (Table S2). We then addressed the presence of SMAR vectors and GFP expression in chimeric pups by analyzing 49 ear punches taken at the time of weaning. The overall MFI was significantly higher in the chimeric biopsies compared with BL6 negative controls (Figures 4B and S4A). No difference was observed in the MFI between

(C) Comparison of fluorescence from ear biopsies of pSMAR (n = 23) and nSMAR (n = 26) chimeras with control mice (n = 3). The statistical analysis was performed using a Brown-Forsythe and Welsch ANOVA with Bunnett's T3 multiple comparison test (***p = 00.004; ****p < 00.001).

(D) Summary of transgene expression from representative chimeric organs. See Table S3 for a complete dataset.

(E) SC contribution to the germline of two male chimeric mice generated with mESCs engineered with nSMAR (mouse 4) and pSMAR (mouse 5). The males were backcrossed with C57BL/6J females and generated 100% agouti litters.

(F) Genotyping PCR from tail biopsies of chimeric mice (F0) and their respective litters (F1). C57BL/6 was used as a negative control. The amplicon corresponds to a 515 bp GFP band. An internal mammalian-conserved *SOX21* sequence (237 bp) was used as an internal control.

(G) PCR amplification of the transgene before (germ cells) and after (sperm) meiosis. The *GFP* amplicon corresponds to the 515 bp band. An internal mammalian conserved *SOX21* sequence (237 bp) was used as an internal control.

(H) Fluorescent images and FACS analysis depicting transgene expression across gametogenesis. GFP fluorescent testis of mouse 4 (nSMAR) and mouse 5 (pSMAR). Constitutively expressing *UBC*:*GFP* mouse (Jackson lab, Pos Ctrl) and a C57BL/6N mouse (Neg Ctrl). (Leica M205FA, exposure 1s, amp gain 1.9x, digital exposure 4, scale bars = 2 mm and 50 μm). FACS analysis was performed in germinal cells from digested seminiferous tubes. The sperm was collected and imaged using a Nikon Ti microscope. Cartoon adapted from (Falcone and Hurd, 2007).

(I) Immunohistochemistry of seminiferous tubules' sections (left = GFP staining with hematoxylin counterstaining, middle = unstained control, right = hematoxylin-eosin staining, scale bars = 100 μm).

pSMAR- and nSMAR-generated chimeras (Figure 4C). We then confirmed via PCR that fluorescence was caused by GFP presence instead of autofluorescence, as GFP could be amplified in 26/49 biopsies (Figure S4B).

Next, we selected five chimeras to analyze the transgene expression in chimeric organs derived from the three germ layers (Figure 4D). GFP was highly expressed in the muscle, skin, and liver and to a lesser extent in the heart and kidney (Figure S4C and Table S3). More interestingly, GFP was also expressed in highly regenerating hematopoietic tissues (i.e., bone marrow, blood, and spleen). Chimeric blood ranged from 17.30% to 63.20%, while bone marrow contained between 3.53% and 56.00% GFP + cells and the chimeric spleens between 4.74% and 55.80% (Figure S4D).

Taken together, these data show the capability of SMAR vectors to survive *in vivo* differentiation from a fertilized egg to a fully developed living organism while persistently expressing the transgene. The injection of engineered mESCs into embryos allowed the generation of *bona fide* chimeras, in some cases reaching almost complete coat chimerism.

SMAR-modified mESCs form chimeras (F0), but genetic modification is not sustained in progeny (F1)

After confirming SMAR mESCs can form chimeras and retain vector expression during cell division and differentiation both *in vitro* and *in vivo*, we assessed whether modified SCs could contribute to the germline (SC transmission) and, most interestingly, whether SMAR vectors survived meiosis and could be passed on to the offspring (vector transmission). Although SMAR vectors are known to replicate episomally and segregate during mitosis (Jenke et al., 2002; Stehle et al., 2007), their ability to persist through meiosis was unknown.

Six chimeras were bred with C57BL/6J mice, and their offspring were analyzed. Males 4 (c39) and 5 (c44), which displayed almost 100% of chinchilla coat color, showed SC transmission, as all their offspring were agouti (Figure 4E), because of the SMAR-modified cells' contribution to the germline. We investigated vector germline transmission by assessing the presence and expression of SMAR vectors in offspring tissues. We did not detect SMAR vectors (*GFP* amplification) in tail biopsies of agouti litters from mouse 4 or 5 (Figure 4F), regardless of the litter (two litters were analyzed per mice). These results suggested that episomal germline transmission was blocked in meiosis, suggesting that the vector was lost during gametogenesis, regardless of which vector was used.

The presence and expression of the vectors were evaluated before and after gametogenesis. For this, testes and sperm from these chimeras were collected and analyzed for both presence (PCR amplification) and expression (fluorescence) of SMAR vectors.

GFP was observed and amplified (Figure 4G) in the testes of both chimeras (Figure 4H top). To exclude that fluorescence was detected from the external testicular membrane or *Tunica Albuginea*, the seminiferous tubules were homogenized to isolate the germinal cells, comprising spermatogonia, spermatocytes, and spermatids. The fluorescence from germinal cells was analyzed by flow cytometry (Figure 4H middle), which revealed between 1.83% and 8.72% of GFP + cells.

Sperm collected from the epididymis was also analyzed. Surprisingly, we could amplify SMAR vectors from sperm lysates (Figure 4G), although fluorescence could only be observed in sperm from a positive control (*UBC*:*GFP*) mouse, but not from SMAR-generated chimeras (Figure 4H bottom), suggesting that the vectors are present throughout spermatogenesis but become silenced during meiosis.

We then analyzed at which stage of meiosis the loss of expression occurred. For this, we performed immunohistochemistry of the germinal epithelia (Figure 4I). Both chimeras showed GFP expression in the most peripheral cell layer (diploid spermatogonia). No GFP could be detected in more advanced meiotic cells, such as spermatocytes, spermatids, or sperm cells. The negative controls showed no GFP expression, whereas constitutively expressing GFP mice showed GFP expression across the germinal epithelia.

These data support our findings that SC pluripotency is not hampered by genetic modification with SMAR vectors, as engineered SCs can generate reproductive organs and contribute to the germline. Additionally, modified mESCs result in viable offspring, suggesting that SMAR vectors do not damage the chromosomal stability. We did not detect SMAR vectors in the F1 generation, suggesting that the vectors do not integrate.

DISCUSSION

A genetic engineering platform that provides safe, efficient, and persistent generation of isogenic SCs has broad application and stands as an alternative to currently used randomly integrating vectors.

Studies using retroviral-mediated modification of SCs often result in poor transduction efficiencies, short-lasting transgene expression (Zhang et al., 2002), or transgene silencing during differentiation (Herbst et al., 2012; Laker et al., 1998), which can be circumvented by adding chromosomal insulators, such as UCOE elements, resulting in maintained transgene expression during hematopoietic differentiation (Müller-Kuller et al., 2015; Pfaff et al., 2013). Other limitations of retroviral vectors include their production and limited cargo capacity (Tiscornia et al.,

Table 2. S/MAR vectors are compatible across multiple transfection technologies

Cells	Technology	Vector	Efficiency	Viability	Conditions
hESC<br>hiPSC	MaxCyte® ExPERT®	pSMAR	82%–85%	87%–91%	Optimization 8<br>1×10^7 cells/ml (50 ul)<br>200–300ug/ml plasmid
	Lipofectamine STEM	pSMAR<br>nSMAR	NA	NA	Clump transfection<br>1–2 ul LipoSTEM + 25 ul OptiMEM<br>500 ng plasmid + 25 ul OptiMEM
	Lipofectamine STEM	pSMAR<br>nSMAR	25%–85%	85%–98%	5×10^4 single cells/well in 24 wp<br>1–2 ul LipoSTEM + 25 ul OptiMEM<br>500 ng plasmid + 25 ul OptiMEM
NHDF	Amaxa II	pSMAR<br>nSMAR	25%–84%	95%–96%	NHDF kit (Program P-022)<br>5×10^5 cells<br>2–10 ug plasmid (100 ul)
	Neon	pSMAR<br>nSMAR	65%–67%	95%–96%	1,650 V, 10 ms 3 pulses<br>1×10^6 cells<br>2 ug plasmid (100 ul)
MEF	Amaxa II	pSMAR<br>nSMAR	40%<br>54%	96%<br>93%	NHDF kit (Program U-020)<br>5×10^5 cells<br>2–10 ug plasmid (100 ul)
mESC<br>miPSC	Amaxa II	pSMAR<br>nSMAR	50%–56%<br>55%–66%	90%–97%<br>90%–97%	Mouse ESC kit (Program A-013)<br>5×10^5 cells<br>2–10 ug plasmid (100 ul)

2006) and the inherent genotoxic risks associated with insertional mutagenesis (Hacein-Bey-Abina et al., 2003).

The DNA vectors described here represent a unique and novel platform and an additional tool for SC modification, offering an advantage not only for clinical and therapeutic applications but also for disease modeling and molecular analysis.

The new generation of SMAR vectors

pEPI (Piechaczek et al., 1999) has been used to modify human hematopoietic progenitors, although transgene silencing was observed in murine SCs due to histone deacetylation (Papapetrou et al., 2006). We also observed a substantial decline in transgene expression in mESCs transfected with pEPI, although episomal forms could be detected in a Southern blot (Figure S1), suggesting vector silencing through similar mechanisms. We refined and improved every component of these vectors, resulting in pSMAR and nSMAR, which can efficiently transfect murine and human SCs, providing stable transgene expression and episomal persistence for up to 170 days (Figures 1 and S1). Removing potentially genotoxic sequences from the bacterial backbone from these vectors reduced perturbation of the host's transcription, improving performance (Figure 2).

SMAR vectors—universal genetic tools

SMAR vectors can modify various primary cells at different stages of differentiation; they can modify fibroblasts while surviving cellular reprogramming, producing genetically modified iPSCs that display all expected pluripotent capabilities (Figure 3). For the first time, we demonstrate that a vector of this class can directly modify iPSCs in their pluripotent state. Additionally, SMAR vectors provide sustained transgene expression through *in vitro* differentiation into specific cell types, producing persistently expressing differentiated progeny (Figure S3). In a more stringent test, we demonstrate that SMAR vectors can survive *in vivo* differentiation, resulting in viable chimeras displaying high levels of transgene expression across their organs (Figures 4 and S4, Table S3). Finally, we demonstrate the vector's flexibility of use across different delivery platforms commonly used in the lab, including chemical or physical transfection methods, such as Neon, Amaxa, or MaxCyte electroporators (Table 2).

Genomic stability and generation of isogenic cells

The genomic stability of SMAR-modified SCs was demonstrated both functionally and molecularly. Cells retain all pluripotent features and differentiate *in vitro* into representatives of all germ layers, while retaining high levels of transgene expression (Figures 3 and S3), and can contribute to generating chimeras (Figures 4 and S4). Removing bacterial sequences from the vector backbone results in higher and more stable transgenic expression during differentiation (Figures 3H and 3I). SMAR vectors safely and persistently modify SCs, while delivering stable levels of

transgene expression during *in vitro* (Figures 3I and S3N) and *in vivo* differentiation—including expression into regenerative hematopoietic organs (Figures 3H and S4).

Modified SCs show little transcriptomic variation, especially when bacterial sequences are removed (Figure 2). We also show that SMAR vectors are maintained as extrachromosomal entities without integration, as we could rescue circular DNA molecules and could not detect any indication of genomic integrations (Figure S1). Analysis of SMAR-generated chimeras also suggest that SMAR vectors are not integrated but are rather sustained episomally, as no vector was transmitted to offspring (Figure 4).

SC versus vector transmission

SMAR vectors have been used to generate transgenic animals (Manzini et al., 2006) (Wagner et al., 2019), where pEPI was directly delivered using sperm-mediated gene transfer. However, the germline transmission of these vectors has not been previously investigated, and the behavior of SMAR vectors during meiosis is not yet understood. We show that SMAR-modified SCs can differentiate into functional gonads (testes), produce functional germ cells (sperm) carrying the vector, and contribute to the offspring (Figure 4). This process, which we refer to as "SC transmission," results in viable F1, providing evidence that the SMAR engineered SCs are not damaged by the vector. However, the inheritance of episomal SMAR vectors during meiosis—"vector transmission"—does not occur. Data indicate that SMAR vectors are present and expressed in reproductive organs (testes), but that transgene expression is gradually lost in meiosis during the spermatogonia/spermatid transition, perhaps by epigenetic mechanisms involved during spermatogenesis (Schagdarsurengin et al., 2012). The result is a mature sperm cell with few silenced copies of SMAR vectors. We believe that the sperm acts as a vector shuttle, delivering copies of the DNA vector into the oocyte, but these fail to establish. Upon fertilization, SMAR vectors need to re-establish to function in this new cellular entity. However, the low copy number and the stochastic nature of establishment represent a very low chance for vector re-establishment after fertilization and contribute to the dilution of few episomal vector copies as the embryo develops, resulting in the loss of replicating SMAR vectors in the F1 generation. Further investigation of SMAR inheritance, including female oogenesis, might provide a deeper understanding of this poorly understood process.

In summary, SMAR vectors can be used as a universal genetic tool for the modification of potentially any cell type, including primary cells, which are typically refractory to genetic manipulation. These vectors can be easily produced in large amounts and can be efficiently delivered to cells with efficiencies above 60%, resulting in the safe generation of genetically engineered isogenic SCs.

EXPERIMENTAL PROCEDURES

Routinely used protocols and materials are included in the supplemental information.

DNA vectors

All DNA vectors were cloned using InFusion HD cloning (Clontech) and following the manufacturer's instructions. An amount of 100 ng of vector and 50 ng of the insert were mixed with water containing the 5x InFusion mix, containing the appropriate buffer and enzyme to allow for homologous recombination between the 15 bp of homology. It was essential that the volume of insert + vector did not exceed 7 μL. In such cases, the InFusion reaction volume was doubled. The recombination took place at 50°C for 15 min. Finally, 2.5 μL of InFusion reaction was transformed into *E. coli* Stellar competent cells (Clontech), following the manufacturers' instructions.

pSMARt CAG:GFP-2A-Puro-SMAR (pSMAR): was generated in three steps. 1) pMAX_SMAR was generated by amplification of the SMAR motif from pEPI-CMV-UCOE (Hagedorn et al., 2013) with primers 1 and 2 and inserted into pMAX_coGFP (Lonza) digested with *SacI* and *PciI*. 2) pMAX_SMAR was digested with *BglII*, and the expression cassette was modified by adding a 2A-Puromycin after the coGFP (primers 3 and 4) to generate pSMARt. 3) The CMV promoter was swapped by the chimeric CAG promoter in the pSMAR vector using the primers 5 and 6 (Table S4).

nSMARt CAG:GFP-2A-Puro-SMAR (nSMAR) was generated by Nature Technology Corporation (NTX).

pSMARt SV40LT-GFP: pSMARt was digested with *BsrGI* to add the insulating Element40, amplified using primers 11 and 12. Then, the 2A-*Puromycin* from pSMARt was cut out with *BmgBI* and *XhoI* and replaced by the *SV40 large T antigen*, amplified with primers 13 and 14 (Table S4).

Cell line generation

A range of different transfection technologies, including Neon, Amaxa, Lipofectamine STEM, and MaxCyte, was tested in this study. Comparative results are shown in Table 2.

Plasmid DNA was delivered (unless otherwise stated) by electroporation using the Amaxa II Nucleofector system (Lonza). For mESC, miPSC, and hESC, 500.000 cells were washed, trypsinized, and resuspended in Mouse ES Cell Nucleofector Kit (VPH-1001) solutions containing between 2 and 10 μg of plasmid DNA. For comparative experiments, equimolar concentrations of plasmid were delivered into cells. The programs A-013 (mESC/miPSC) and A-023 (hESC) were used. After electroporation, the cells were carefully transferred into feeder plates in media without antibiotics. After 24 h, the media were replaced by the respective complete media containing antibiotics and G418 (1 mg/ml) or 1 μg/ml Puromycin selection, if needed. The cells were kept under selection for a month, and the media were replaced every second day.

For feeder-free transfection of hiPSCs, cells were plated in small clumps at a density of 20%–30% or as 50.000 single cells in a 24-well plate. The next day, cells were transfected using Lipofectamine Stem as of manufacturers recommendation, using 2 μL or 1 μL of transfection reagent diluted in 25 μL OptiMEM, mixed with 500 ng DNA diluted in 25 μL OptiMEM. For comparative experiments, equimolar concentrations of vectors were used. For establishment using antibiotics, 24 h after transfection, cells were selected with media containing 0.5 μg/ml Puromycin and kept under selection for two weeks. For establishment with FACS, GFP + cells were sorted and further cultured 6, 12, 28, and 44 dpt. Media were replaced three times per week.

For fibroblasts (MEFs and HDFs), 500.000 cells were washed, trypisinized and resuspended in NHDF Electroporation Kit (VPD-1001) solutions containing between 2 and 10 μg of plasmid DNA. For comparative experiments, equimolar concentrations of plasmid were delivered into cells. Programs U-020 (MEFs) or P-022 (HDFs) were used in the Amaxa Nucleofector II device (Lonza). Finally, the electroporated cells were gently pipetted and transferred into a gelatin-coated 6-well plate with DMEM (Gibco) containing 10% FCS (Gibco) without selection nor antibiotics and allowed to recover. After 24 h, antibiotics were added to the media as well as G418 (1 mg/ml) or 1 μg/ml Puromycin selection, if needed. The cells were kept under selection for a month, and the media were replaced every second day.

Quantification and data analysis

Unless otherwise stated, statistical analysis was performed using Graphpad Prism 8. T test (with or without Welch's correction) was used for statistical analysis unless otherwise specified. For all statistical analyzes, a value of $p < 0.05$ was considered statistically significant.

Contact for reagents and resource sharing

Further information and requests for resources and reagents should be directed to the Lead Contact, Richard Harbottle (r.harbottle@dkfz.de).

Data and software availability

The microarray data discussed in this publication have been deposited in NCBI's gene Expression Omnibus (Edgar et al., 2002) and are accessible through GEO Series accession number GSE142299.

SUPPLEMENTAL INFORMATION

Supplemental information can be found online at https://doi.org/10.1016/j.stemcr.2021.11.011.

AUTHOR CONTRIBUTIONS

R.P.H. and A.R.M. formulated the concept. M.Bo. and A.R.M. designed and generated the vectors. A.R.M. carried out the murine molecular and biological experiments, as well as *in vitro* and *in vivo* experiments. M.U. carried out the human molecular and biological experiments. M.Bu., S.S., and A.R.M. performed the hematopoietic differentiation and analysis of hematopoietic tissues. L.B. generated hESC lines for RNAseq, and J.P. performed the bioinformatic analysis. F.v.H. performed the pronuclear and blastocyst injections. K.M.D. processed the transgenic tissues and provided technical support. T.M. and M.M. provided technical support and scientific advice. A.R.M., M.U., and R.P.H. prepared the manuscript.

CONFLICTS OF INTERESTS

M.Bo. and R.H. have patent applications related to this work filed by the DKFZ and NTC (WO2019057774A1, filed 19 September 2018, published 28 March 2019). (WO2019060253A1, filed 17 September 2018, published 28 March 2019).

A.R.M. currently works at MaxCyte Inc.

ACKNOWLEDGMENTS

A.R.M. and M.U. were supported by a DKFZ Graduate Program fellowship and funding from Nature Technology Corporation.

We thank Prof. Hans Lipps and Dr. Claudia Hagedorn (University of Witten) for providing us with pEPI-CMV-UCOE. We thank the following for their contributions to this paper: Prof Dr. Christine Engeland for her help with the ethical approval of the urinary cell study. Dr. Joschka Willemsen, Dr. Jamie Frankish, and Sandra Bastian (Virus Associated Carcinogenesis, DKFZ) for production of lentiviral particles; Andrea Pohl-Arnold and Stephanie Laier (Tumor Models department, DKFZ) for the dissection and processing of transgenic organs; Andrea Rausch, Heinrich Steinbauer, and Dr. Johannes Schenkel (Cryopreservation Service, DKFZ) for the collection of transgenic sperm; Ornella Kossi and Vanessa Vogel (Hi-STEM), for their support with immunohistochemistry stainings. Dr. Edward Green for his input during the writing of this manuscript. Finally, we thank the DKFZ Genomics and Proteomics Core Facility for providing the Illumina Whole Genome Expression Beadchips and related services. Some figures were created with BioRender.com.

Received: October 15, 2020
Revised: November 23, 2021
Accepted: November 24, 2021
Published: December 22, 2021

SUPPORTING CITATIONS

The following references appear in the supplemental information: Blighe et al., 2021; Evans and Kaufman, 1981; Gu et al., 2016; Hooper et al., 1987; Koressaar and Remm, 2007; Koutsoudakis et al., 2006; Maetzig et al., 2014; Mulder et al., 2020; Neufang et al., 2001; Phipson et al., 2016; Schindelin et al., 2012; Ritchie et al., 2015; Shi et al., 2010; Tesar, 2005; Untergasser et al., 2012; Varisli et al., 2013; Willemsen et al., 2017; Zufferey et al., 1998; Zufferey et al., 1997.

REFERENCES

Argyros, O., Wong, S.P., Fedonidis, C., Tolmachov, O., Waddington, S.N., Howe, S.J., Niceta, M., Coutelle, C., and Harbottle, R.P. (2011). Development of SMAR minicircles for enhanced and persistent transgene expression in the mouse liver. J. Mol. Med. *89*, 515–529.

Argyros, O., Wong, S.P., Niceta, M., Waddington, S.N., Howe, S.J., Coutelle, C., Miller, A.D., and Harbottle, R.P. (2008). Persistent episomal transgene expression in liver following delivery of a scaffold/matrix attachment region containing non-viral vector. Gene Ther. *15*, 1593–1605.

Blighe, K., Rana, S., and Lewis, M. (2021). EnhancedVolcano: Publication-ready volcano plots with enhanced colouring and labeling. R package version 1.12.0, https://github.com/kevinblighe/EnhancedVolcano. (Bioconductor).

Bozza, M., Green, E.W., Espinet, E., De Roia, A., Klein, C., Vogel, V., Offringa, R., Williams, J.A., Sprick, M., and Harbottle, R.P. (2020). Novel non-integrating DNA nano-SMAR vectors restore gene function in isogenic patient-derived pancreatic tumor models. Mol. Ther. *17*, 957–968.

Canaan, A., Haviv, I., Urban, A.E., Schulz, V.P., Hartman, S., Zhang, Z., Palejev, D., Deisseroth, A.B., Lacy, J., Snyder, M., et al. (2009). EBNA1 regulates cellular gene expression by binding cellular promoters. Proc. Natl. Acad. Sci. U S A *106*, 22421–22426.

Cattoglio, C., Facchini, G., Sartori, D., Antonelli, A., Miccio, A., Cassani, B., Schmidt, M., von Kalle, C., Howe, S., Thrasher, A., J., et al. (2007). Hot spots of retroviral integration in human CD34+ hematopoietic cells. Blood *110*, 1770–1778.

Chen, Y.T., Furushima, K., Hou, P.S., Ku, A.T., Deng, J.M., Jang, C.W., Fang, H., Adams, H.P., Kuo, M.L., Ho, H.N., et al. (2009). PiggyBac transposon-mediated, reversible gene transfer in human embryonic stem cells. Stem Cells Dev., 763–771. https://home.liebertpub.com/scd 19.

Czerwińska, P., Mazurek, S., Kołodziejczak, I., and Wiznerowicz, M. (2019). Gene delivery methods and genome editing of human pluripotent stem cells. Rep. Pract. Oncol. Radiother. *24*, 180–187.

Edgar, R., Domrachev, M., and Lash, A.E. (2002). Gene Expression Omnibus: NCBI gene expression and hybridization array data repository. Nucleic Acids Res. *30*, 207–210.

Evans, M.J., and Kaufman, M.H. (1981). Establishment in culture of pluripotential cells from mouse embryos. Nature *292*, 154–156.

Falcone, T., and Hurd, W.W. (2007). Clinical Reproductive Medicine and Surgery (Elsevier Health Sciences).

Fregien, N., and Davidson, N. (1986). Activating elements in the promoter region of the chicken beta-actin gene. Gene *48*, 1–11.

Gu, Z., Eils, R., and Schlesner, M. (2016). Complex heatmaps reveal patterns and correlations in multidimensional genomic data. Bioinformatics *32*, 2847–2849.

Haase, R., Argyros, O., Wong, S.P., Harbottle, R.P., Lipps, H.J., Ogris, M., Magnusson, T., Pinto, M., Haas, J., and Baiker, A. (2010). pEPito: a significantly improved non-viral episomal expression vector for mammalian cells. BMC Biotechnol. *10*, 14–20.

Hacein-Bey-Abina, S., Kalle, von, C., Schmidt, M., Le Deist, F., Wulffraat, N., McIntyre, E., Radford, I., Villeval, J.L., Fraser, C.C., Cavazzana-Calvo, M., et al. (2003). A serious adverse event after successful gene therapy for X-linked severe combined immunodeficiency. N. Engl. J. Med. *348*, 255–256.

Hagedorn, C., Antoniou, M.N., and Lipps, H.J. (2013). Genomic cis-acting sequences improve expression and establishment of a nonviral vector. Mol. Ther. Nucleic Acids *2*, e118. https://doi.org/10.1038/mtna.2013.47.

Hagedorn, C., Baiker, A., Postberg, J., Ehrhardt, A., and Lipps, H.J. (2012). Handling SMAR vectors. Cold Spring Harb Protoc. *2012*, 657–663.

Hagedorn, C., Gogol-Döring, A., Schreiber, S., Epplen, J.T., and Lipps, H.J. (2017). Genome-wide profiling of SMAR-based replicon contact sites. Nucleic Acids Res. *45*, 7841–7854.

Hagedorn, C., and Lipps, H.J. (2013). pEPI for gene therapy non viral episomes and their application in somatic gene therapy. J. Cell Sci. Ther. *04*, 1–8.

He, S., Nakada, D., and Morrison, S.J. (2009). Mechanisms of stem cell self-renewal. Annu. Rev. Cell Dev. Biol. *25*, 377–406.

Herbst, F., Ball, C., R., Tuorto, F., Nowrouzi, A., Wang, W., Zavidij, O., Dieter, S., M., Fessler, S., van der Hoeven, F., Kloz, U., et al. (2012). Extensive methylation of promoter sequences silences lentiviral transgene expression during stem cell differentiation in vivo. Mol. Ther. *20*, 1014–1021.

Hooper, M., Hardy, K., Handyside, A., Hunter, S., and Monk, M. (1987). HPRT-deficient (Lesch-Nyhan) mouse embryos derived from germline colonization by cultured cells. Nature *326*, 292–295.

Humme, S., Reisbach, G., Feederle, R., Delecluse, H.-J., Bousset, K., Hammerschmidt, W., and Schepers, A. (2003). The EBV nuclear antigen 1 (EBNA1) enhances B cell immortalization several thousandfold. Proc. Natl. Acad. Sci. U S A *100*, 10989–10994.

Jenke, B.H.C., Fetzer, C.P., Stehle, I.M., Jönsson, F., Fackelmayer, F.O., Conradt, H., Bode, J., and Lipps, H.J. (2002). An episomally replicating vector binds to the nuclear matrix protein SAF-A in vivo. EMBO Rep. *3*, 349–354.

Kim, E.J., Kang, K.H., and Ju, J.H. (2017). CRISPR-Cas9: a promising tool for gene editing on induced pluripotent stem cells. Korean J. Intern. Med. *32*, 42–61.

Koressaar, T., and Remm, M. (2007). Enhancements and modifications of primer design program Primer3. Bioinformatics *23*, 1289–1291.

Kotterman, M.A., Chalberg, T.W., and Schaffer, D.V. (2015). Viral vectors for gene therapy: translational and clinical outlook. Annu. Rev. Biomed. Eng. *17*, 63–89.

Koutsoudakis, G., Kaul, A., Steinmann, E., Kallis, S., Lohmann, V., Pietschmann, T., and Bartenschlager, R. (2006). Characterization of the early steps of hepatitis C virus infection by using luciferase reporter viruses. J. Virol. *80*, 5308–5320.

Laker, C., Meyer, J., Schopen, A., Friel, J., Heberlein, C., Ostertag, W., and Stocking, C. (1998). Host cis-mediated extinction of a retrovirus permissive for expression in embryonal stem cells during differentiation. J. Virol. *72*, 339–348.

Lufino, M.M.P., Manservigi, R., and Wade-Martins, R. (2007). An SMAR-based infectious episomal genomic DNA expression vector provides long-term regulated functional complementation of LDLR deficiency. Nucleic Acids Res. *35*, e98.

Luke, J., Carnes, A.E., Hodgson, C.P., and Williams, J.A. (2009). Improved antibiotic-free DNA vaccine vectors utilizing a novel RNA based plasmid selection system. Vaccine *27*, 6454–6459.

Manzini, S., Vargiolu, A., Seruggia, D., Cerrito, M., and Busnelli, M. (2010). SMAR trek: a new generation of non-viral episomal vectors to be used in gene transfer experiments. Transgenic Res., 307–355.

Maetzig, T., Kuehle, J., Schwarzer, A., Turan, S., Rothe, M., Chaturvedi, A., Morgan, M., Ha, T.C., Heuser, M., Hammerschmidt, W., et al. (2014). All-in-One inducible lentiviral vector systems based on drug controlled FLP recombinase. Biomaterials *35*, 4345–4356. https://doi.org/10.1016/j.biomaterials.2014.01.057.

Manzini, S., Vargiolu, A., Stehle, I.M., Bacci, M.L., Cerrito, M.G., Giovannoni, R., Zannoni, A., Bianco, M.R., Forni, M., Donini, P., et al. (2006). Genetically modified pigs produced with a nonviral episomal vector. Proc. Natl. Acad. Sci. U S A *103*, 17672–17677.

Miyazaki, J., Takaki, S., Araki, K., Tashiro, F., Tominaga, A., Takatsu, K., and Yamamura, K. (1989). Expression vector system based on the chicken beta-actin promoter directs efficient production of interleukin-5. Gene *79*, 269–277.

Mulder, J., Sharmin, S., Chow, T., Rodrigues, D.C., Hildebrandt, M.R., D'Cruz, R., Rogers, I., Ellis, J., and Rosenblum, N.D. (2020). Generation of infant- and pediatric-derived urinary induced pluripotent stem cells competent to form kidney organoids. Pediatr. Res. *87*, 647–655.

Müller-Kuller, U., Ackermann, M., Kolodziej, S., Brendel, C., Fritsch, J., Lachmann, N., Kunkel, H., Lausen, J., Schambach, A., Moritz, T., et al. (2015). A minimal ubiquitous chromatin opening element (UCOE) effectively prevents silencing of juxtaposed heterologous promoters by epigenetic remodeling in multipotent and pluripotent stem cells. Nucleic Acids Res. *43*, 1577–1592.

Neufang, G., Furstenberger, G., Heidt, M., Marks, F., and Müller-Decker, K. (2001). Abnormal differentiation of epidermis in transgenic mice constitutively expressing cyclooxygenase-2 in skin. Proc. Natl. Acad. Sci. U S A *98*, 7629–7634.

Okita, K., Matsumura, Y., Sato, Y., Okada, A., Morizane, A., Okamoto, S., Hong, H., Nakagawa, M., Tanabe, K., Tezuka, K.I., et al. (2011). A more efficient method to generate integration-free human iPS cells. Nat. Methods *8*, 409–412.

Orbán, T.I., Apáti, A., Németh, A., Varga, N., Krizsik, V., Schamberger, A., Szebényi, K., Erdei, Z., Várady, G., Karászi, É., et al. (2009). Applying a "double-feature" promoter to identify cardiomyocytes differentiated from human embryonic stem cells following transposon-based gene delivery. Stem Cells *27*, 1077–1087.

Papapetrou, E.P., Ziros, P.G., Micheva, I.D., Zoumbos, N.C., and Athanassiadou, A. (2006). Gene transfer into human hematopoietic progenitor cells with an episomal vector carrying an SMAR element. Gene Ther. *13*, 40–51.

Park, M.A., Jung, H.S., and Slukvin, I. (2018). Genetic engineering of human pluripotent stem cells using PiggyBac transposon system. Curr. Protoc. Stem Cell Biol. *47*, e63.

Pfaff, N., Lachmann, N., Ackermann, M., Kohlscheen, S., Brendel, C., Maetzig, T., Niemann, H., Antoniou, M.N., Grez, M., Schambach, A., et al. (2013). A ubiquitous chromatin opening element prevents transgene silencing in pluripotent stem cells and their differentiated progeny. Stem Cells *31*, 488–499.

Phipson, B., Lee, S., Majewski, I.J., Alexander, W.S., and Smyth, G.K. (2016). Robust hyperparameter estimation protects against hypervariable genes and improves power to detect differential expression. Ann. Appl. Stat. *10*, 946–963.

Piechaczek, C., Fetzer, C., Baiker, A., Bode, J., and Lipps, A.H.J. (1999). A vector based on the SV40 origin of replication and chromosomal SMARs replicates episomally in CHO cells. Nucleic Acids Res. *27*, 1–3.

Querques, et al. (2019). A highly soluble Sleeping Beauty transposase improves control of gene insertion. Nat. Biotechnol. *37*, 1502–1512.

Ritchie, M.E., Phipson, B., Wu, D., Hu, Y., Law, C.W., Shi, W., and Smyth, G.K. (2015). Limma powers differential expression analyses for RNA-sequencing and microarray studies. Nucleic Acids Res. *43*, e47.

Schagdarsurengin, U., Paradowska, A., and Steger, K. (2012). Analysing the sperm epigenome: roles in early embryogenesis and assisted reproduction. Nat. Rev. Urol. *9*, 609–619.

Schindelin, et al. (2012). Fiji: an open-source platform for biological-image analysis. Nat. Methods *9*, 676–682.

Schwanke, K., Merkert, S., Kempf, H., Hartung, S., Jara-Avaca, M., Templin, C., Göhring, G., Haverich, A., Martin, U., and Zweigerdt, R. (2014). Fast and efficient multitransgenic modification of human pluripotent stem cells. Hum. Gene Ther. Methods *25*, 136–153.

Shi, W., Oshlack, A., and Smyth, G.K. (2010). Optimizing the noise versus bias trade-off for Illumina whole genome expression BeadChips. Nucleic Acids Res. *38*, e204.

Song, M., and Ramakrishna, S. (2018). Genome editing in stem cells for disease therapeutics. Mol. Biotechnol. *60*, 329–338.

Stehle, I.M., Postberg, J., Rupprecht, S., Cremer, T., Jackson, D.A., and Lipps, H.J. (2007). Establishment and mitotic stability of an extra-chromosomal mammalian replicon. BMC Cell Biol. *6*, 8–33. https://doi.org/10.1186/1471-2121-8-33.

Stehle, I.M., Scinteie, M.F., Baiker, A., Jenke, A.C.W., and Lipps, H.J. (2003). Exploiting a minimal system to study the epigenetic control of DNA replication: the interplay between transcription and replication. Chromosome Res. *11*, 413–421.

Sugden, B., Marsh, K., and Yates, J. (1985). A vector that replicates as a plasmid and can be efficiently selected in B-lymphoblasts transformed by Epstein-Barr virus. Mol. Cell. Biol. *5*, 410–413.

Sung, N.S., and Pagano, J.S. (1995). Molecular mechanisms of transformation by epstein-barr virus. In DNA Tumor Viruses, G. Barbanti-Brodano, M. Bendinelli, and H. Friedman, eds. (Springer US), pp. 1–43.

Takahashi, K., and Yamanaka, S. (2013). Induced pluripotent stem cells in medicine and biology. Development *140*, 2457–2461.

Takahashi, K., and Yamanaka, S. (2006). Induction of pluripotent stem cells from mouse embryonic and adult fibroblast cultures by defined factors. Cell *126*, 663–676.

Tesar, P.J. (2005). Derivation of germ-line-competent embryonic stem cell lines from preblastocyst mouse embryos. Proc. Natl. Acad. Sci. U S A *102*, 8239–8244.

Thyagarajan, B., Scheyhing, K., Xue, H., Fontes, A., Chesnut, J., Rao, M., and Lakshmipathy, U. (2009). A single EBV-based vector for stable episomal maintenance and expression of GFP in human embryonic stem cells. Regen. Med. *4*, 239–250.

Tiscornia, G., Singer, O., and Verma, I.M. (2006). Production and purification of lentiviral vectors. Nat. Protoc. *1*, 241–245.

Untergasser, A., Cutcutache, I., Koressaar, T., Ye, J., Faircloth, B.C., Remm, M., and Rozen, S.G. (2012). Primer3–new capabilities and interfaces. Nucleic Acids Res. *40*, e115.

Varisli, O., Agca, C., and Agca, Y. (2013). Short-term storage of rat sperm in the presence of various extenders. J. Am. Assoc. Lab. Anim. Sci. *52*, 732–737.

Wagner, S., McCracken, J., Bruszies, S., Broadhurst, R., Wells, D.N., Oback, B., Bode, J., and Laible, G. (2019). Episomal minicircles persist in periods of transcriptional inactivity and can be transmitted through somatic cell nuclear transfer into bovine embryos. Mol. Biol. Rep. *13*, 1677–1710.

Warlich, E., Kuehle, J., Cantz, T., Brugman, M.H., Maetzig, T., Galla, M., Filipczyk, A.A., Halle, S., Klump, H., Schöler, H.R., et al. (2011). Lentiviral vector design and imaging approaches to visualize the early stages of cellular reprogramming. Mol. Ther. *19*, 782–789.

Wilber, A., Linehan, J.L., Tian, X., Woll, P.S., Morris, J.K., Belur, L.R., McIvor, R.S., and Kaufman, D.S. (2007). Efficient and stable transgene expression in human embryonic stem cells using transposon-mediated gene transfer. Stem Cells *25*, 2919–2927.

Willemsen, J., Wicht, O., Wolanski, J.C., Baur, N., Bastian, S., Haas, D., A, Matula, P., Knapp, B., Meyniel-Schicklin, L., Wang, C., et al. (2017). Phosphorylation-dependent feedback inhibition of RIG-I by DAPK1 identified by kinome-wide siRNA screening. Mol. Cell *65*, 403–415.e8.

Wong, S.P., Argyros, O., Coutelle, C., and Harbottle, R.P. (2011). Non-viral SMAR vectors replicate episomally in vivo when provided with a selective advantage. Gene Ther. *18*, 82–87.

Yates, J.L., Warren, N., and Sugden, B. (1985). Stable replication of plasmids derived from Epstein-Barr virus in various mammalian cells. Nature *313*, 812–815.

Zhang, X.-Y., La Russa, V.F., Bao, L., Kolls, J., Schwarzenberger, P., and Reiser, J. (2002). Lentiviral vectors for sustained transgene expression in human bone marrow-derived stromal cells. Mol. Ther. *5*, 555–565.

Zufferey, R., Dull, T., Mandel, R.J., Bukovsky, A., Quiroz, D., Naldini, L., and Trono, D. (1998). Self-inactivating lentivirus vector for safe and efficient in vivo gene delivery. J. Virol. *72*, 9873–9880.

Zufferey, R., Nagy, D., Mandel, R.J., Naldini, L., and Trono, D. (1997). Multiply attenuated lentiviral vector achieves efficient gene delivery in vivo. Nat. Biotechnol. *15*, 871–875.

Stem Cell Reports, Volume *17*

Supplemental Information

An episomal DNA vector platform for the persistent genetic modification of pluripotent stem cells and their differentiated progeny

Alicia Roig-Merino, Manuela Urban, Matthias Bozza, Julia D. Peterson, Louise Bullen, Marleen Büchler-Schäff, Sina Stäble, Franciscus van der Hoeven, Karin Müller-Decker, Tristan R. McKay, Michael D. Milsom, and Richard P. Harbottle

SUPPLEMENTAL INFORMATION

Figure S1

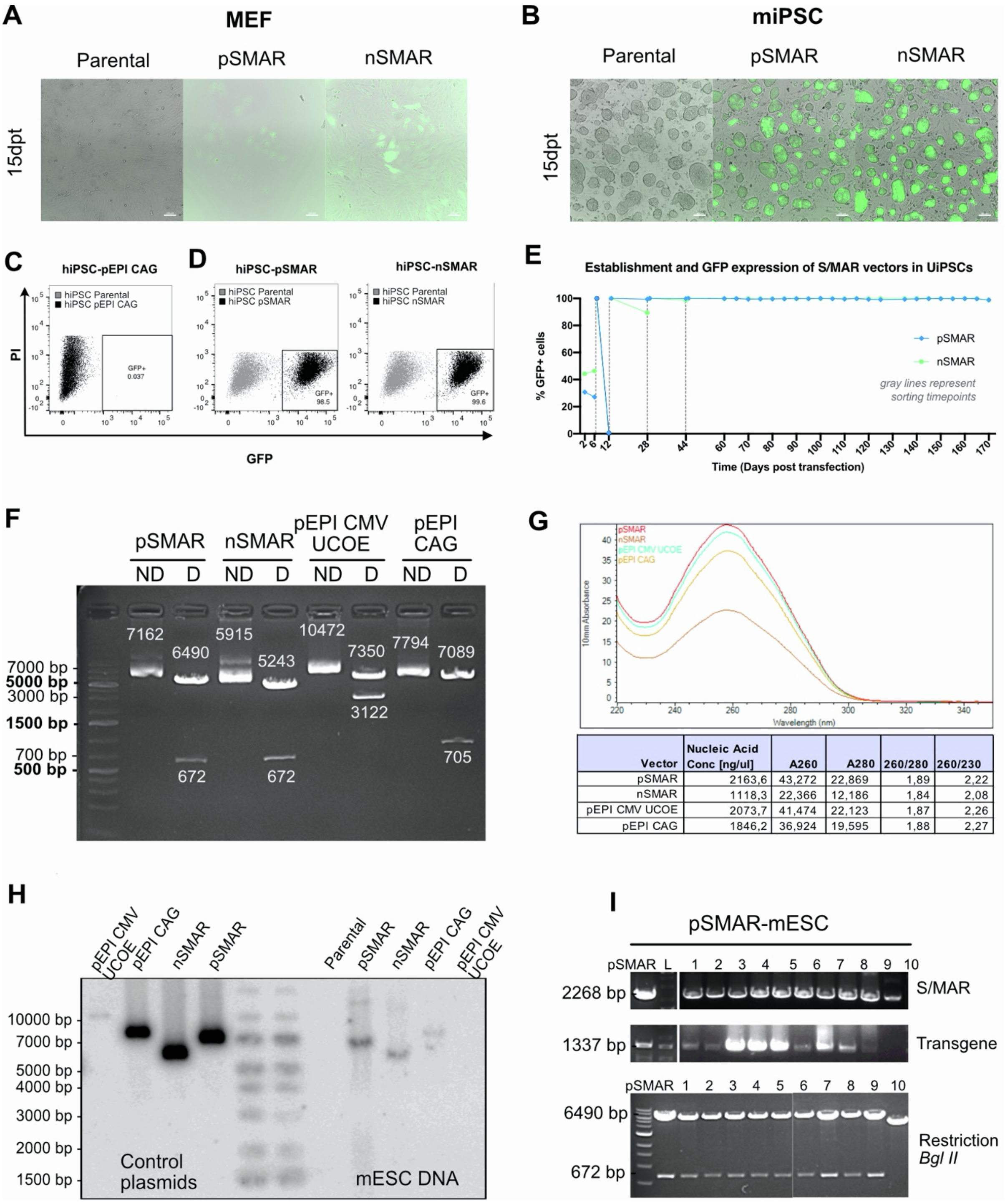

Vector	Nucleic Acid Conc [ng/ul]	A260	A280	260/280	260/230
pSMAR	2163,6	43,272	22,869	1,89	2,22
nSMAR	1118,3	22,366	12,186	1,84	2,08
pEPI CMV UCOE	2073,7	41,474	22,123	1,87	2,26
pEPI CAG	1846,2	36,924	19,595	1,88	2,27

Figure S1 : Confirmation of vector integrity and performance in other primary cells

(A) MEF cell line generated with pSMAR and nSMAR. Fluorescent images of %GFP+ cells and MFI, 15 days after transfection (Scale bars = 100µm).
(B) miPSC cell line generated with pSMAR and nSMAR. Fluorescent images of %GFP+ cells and MFI, 15 days after transfection (Scale bars = 100µm).
(C) Flow Cytometry analysis of outgrowing hiPSC-pEPI-CAG cells after G418 selection.
(D) NHDF-derived hiPSCs generated with pSMAR and nSMAR. Flow Cytometry analysis of GFP expression in parental and modified hiPSCs, 3 months after transfection and >2 months without selection.
(E) Passive establishment (antibiotic selection-free) and GFP expression of pSMAR and nSMAR vectors in urinary-derived hiPSC (UiPSC) over a period of 170 days. The gray lines represent FACS sorting timepoints.
(F) DNA electrophoresis assessing vector integrity. ND=non-digested, D=digested with *BglII*.
(G) Spectrophotometric analysis of DNA vector quality
(H) Southern Blot analysis of mESCs showing the integrity of the DNA vectors
(I) Plasmid rescue of pSMAR from stably transfected mESCs. PCR amplification of the SMAR motif and transgene (n=10 bacterial clones)

Figure S2

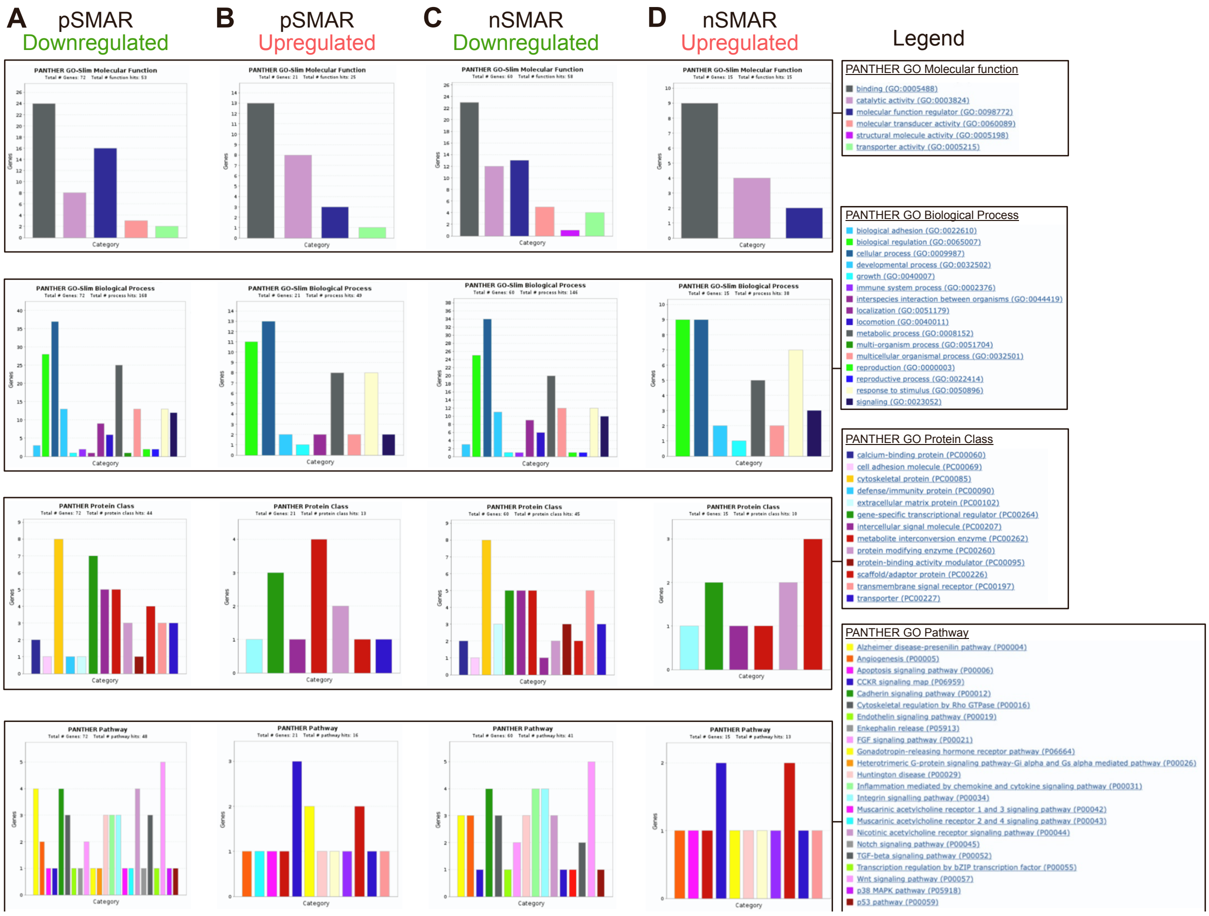

Figure S2: SMAR NanoVectors have a minimal impact on the cells' transcriptome

GO TERM analysis performed on the full list of significantly down or upregulated genes in pSMAR or nSMAR transfected hESCs using GO terms and the PANTHER classification system.

Figure S3

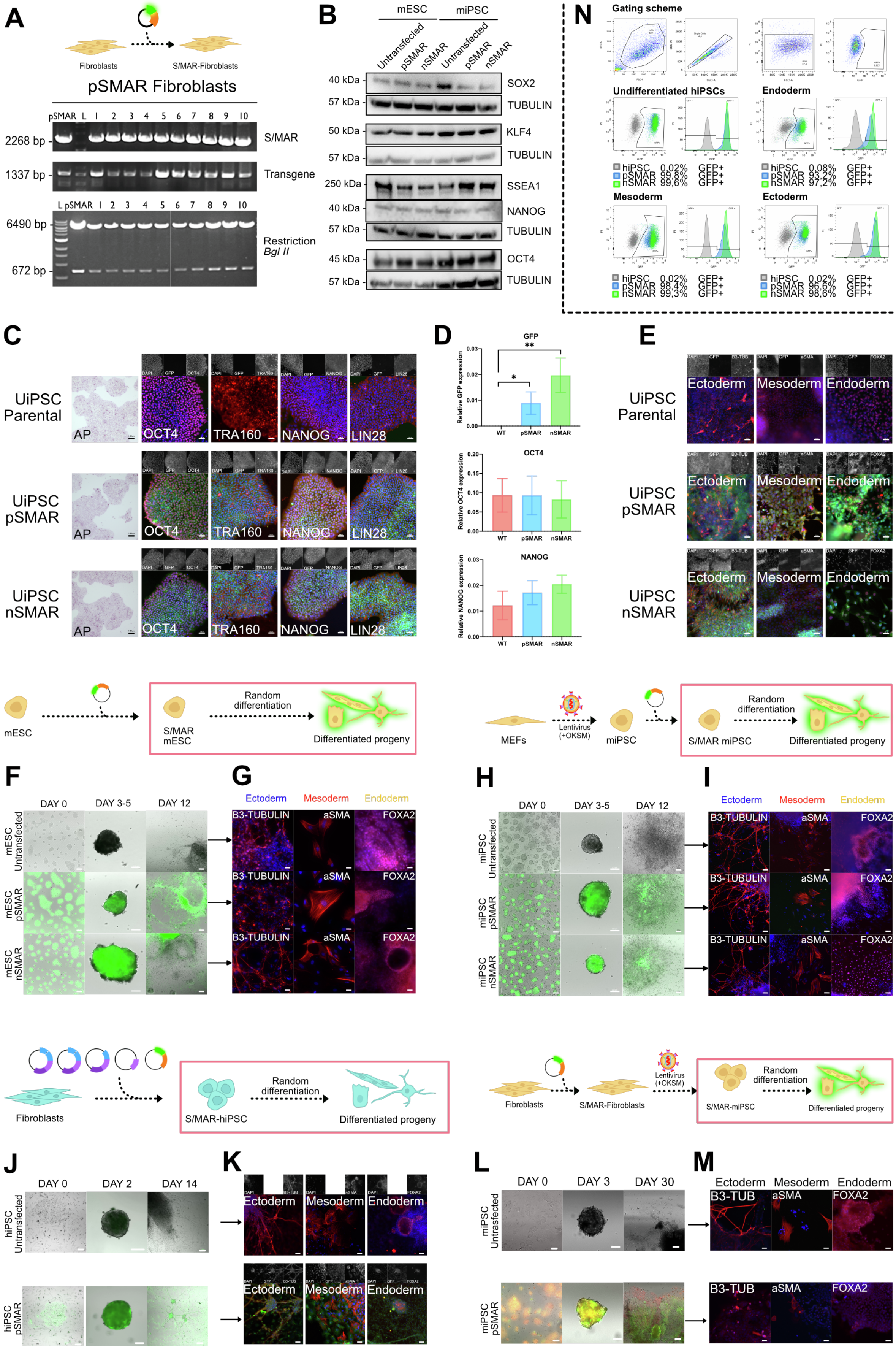

A
Fibroblasts
S/MAR-Fibroblasts
pSMAR Fibroblasts
2268 bp
S/MAR
1337 bp
Transgene
6490 bp
672 bp
Restriction Bgl II
B
mESC
miPSC
Untransfected
pSMAR
nSMAR
SOX2
TUBULIN
KLF4
SSEA1
NANOG
OCT4
N
Gating scheme
Undifferentiated hiPSCs
Endoderm
Mesoderm
Ectoderm
hiPSC 0,02% GFP+
pSMAR 99,8% GFP+
nSMAR 99,6% GFP+
hiPSC 0,08% GFP+
pSMAR 93,2% GFP+
nSMAR 97,2% GFP+
hiPSC 0,02% GFP+
pSMAR 98,4% GFP+
nSMAR 99,3% GFP+
hiPSC 0,02% GFP+
pSMAR 96,6% GFP+
nSMAR 98,6% GFP+
C
UiPSC Parental
UiPSC pSMAR
UiPSC nSMAR
AP
OCT4
TRA160
NANOG
LIN28
D
GFP
OCT4
NANOG
Relative GFP expression
Relative OCT4 expression
Relative NANOG expression
WT
pSMAR
nSMAR
E
Ectoderm
Mesoderm
Endoderm
mESC
S/MAR mESC
Random differentiation
Differentiated progeny
MEFs
Lentivirus (+OKSM)
miPSC
S/MAR miPSC
F
G
H
I
DAY 0
DAY 3-5
DAY 12
mESC Untransfected
mESC pSMAR
mESC nSMAR
miPSC Untransfected
miPSC pSMAR
miPSC nSMAR
B3-TUBULIN
aSMA
FOXA2
Fibroblasts
S/MAR-hiPSC
S/MAR-Fibroblasts
S/MAR-miPSC
J
K
L
M
DAY 2
DAY 14
DAY 3
DAY 30
hiPSC Untransfected
hiPSC pSMAR
miPSC Untrasfected
miPSC pSMAR
B3-TUB

Figure S3: SMAR vectors remain episomal and do not impair Stem Cells' pluripotency

(A) Plasmid rescue of pSMAR from stably transfected MEFs. PCR amplification of the SMAR motif and transgene. Restriction digestion of 10 clones with BglII
(B) Western Blot of pluripotency markers of mESCs and miPSCs generated from CF1-MEFs upon transduction with pWPI 4in1 Lentiviral vector
(C) Pluripotency staining of human UiPSCs reprogrammed using EBNA vectors and modified at the iPSC level (Scale bars = 100µm).
(D) Quantification of GFP and pluripotency markers in UiPSC. qRT-PCR analysis of transgene and the pluripotency markers Oct4 and Nanog, relative to GAPDH. 1-WAY ANOVA and Unpaired T-Tests were used for statistical analysis of three independent differentiations of the same cells (n=3, **p-val = 0,0073, *p-val=0,0236).
(E) Trilineage differentiation of UiPSC modified with pSMAR or nSMAR at the iPSC level (b3-Tubulin, ectoderm; aSMA, mesoderm; FoxA2, endoderm, Scale bars = 100µm).
(F) Random differentiation via EBs of parental and pSMAR-mESC, modified at the mESC level (Scale bars = 100µm).
(G) Exemplary immunofluorescence staining of germ layer derivatives (b3-Tubulin, ectoderm; aSMA, mesoderm; FoxA2, endoderm) from parental and pSMAR-mESCs (Scale bars = 100µm).
(H) Random differentiation via EBs of parental and pSMAR-miPSCs, derived from CF1-MEFs modified at the fibroblast level and reprogrammed using 4in1 Lentivirus (Scale bars = 100µm).
(I) Exemplary immunofluorescence staining of germ layer derivatives (b3-Tubulin, ectoderm; aSMA, mesoderm; FoxA2, endoderm) from parental and pSMAR-miPSCs (Scale bars = 100µm).
(J) Random differentiation via EBs of parental and pSMAR-hiPSC derived from NHDFs modified at the fibroblast level, and reprogrammed using EBNA vectors (Scale bars = 100µm).
(K) Exemplary immunofluorescence staining of germ layer derivatives (b3-Tubulin, ectoderm; aSMA, mesoderm; FoxA2, endoderm) from parental and pSMAR-hiPSCs (Scale bars = 100µm).
(L) Random differentiation via EBs of parental and pSMAR-miPSC, modified at the fibroblast level and reprogrammed using 4in1 Lentivirus (dTom) (Scale bars = 100µm).
(M) Exemplary immunofluorescence staining of germ layer derivatives (b3-Tubulin, ectoderm; aSMA, mesoderm; FoxA2, endoderm) from parental and pSMAR-miPSCs (Scale bars = 100µm).
(N) Quantification of transgene expression in differentiated progeny of genetically modified hiPSCs Gating scheme for GFP-quantification experiments. Dot plots and histograms depicting persistance of GFP expression from pSMAR or nSMAR engineered hiPSC, separated by germ-layer

Figure S4

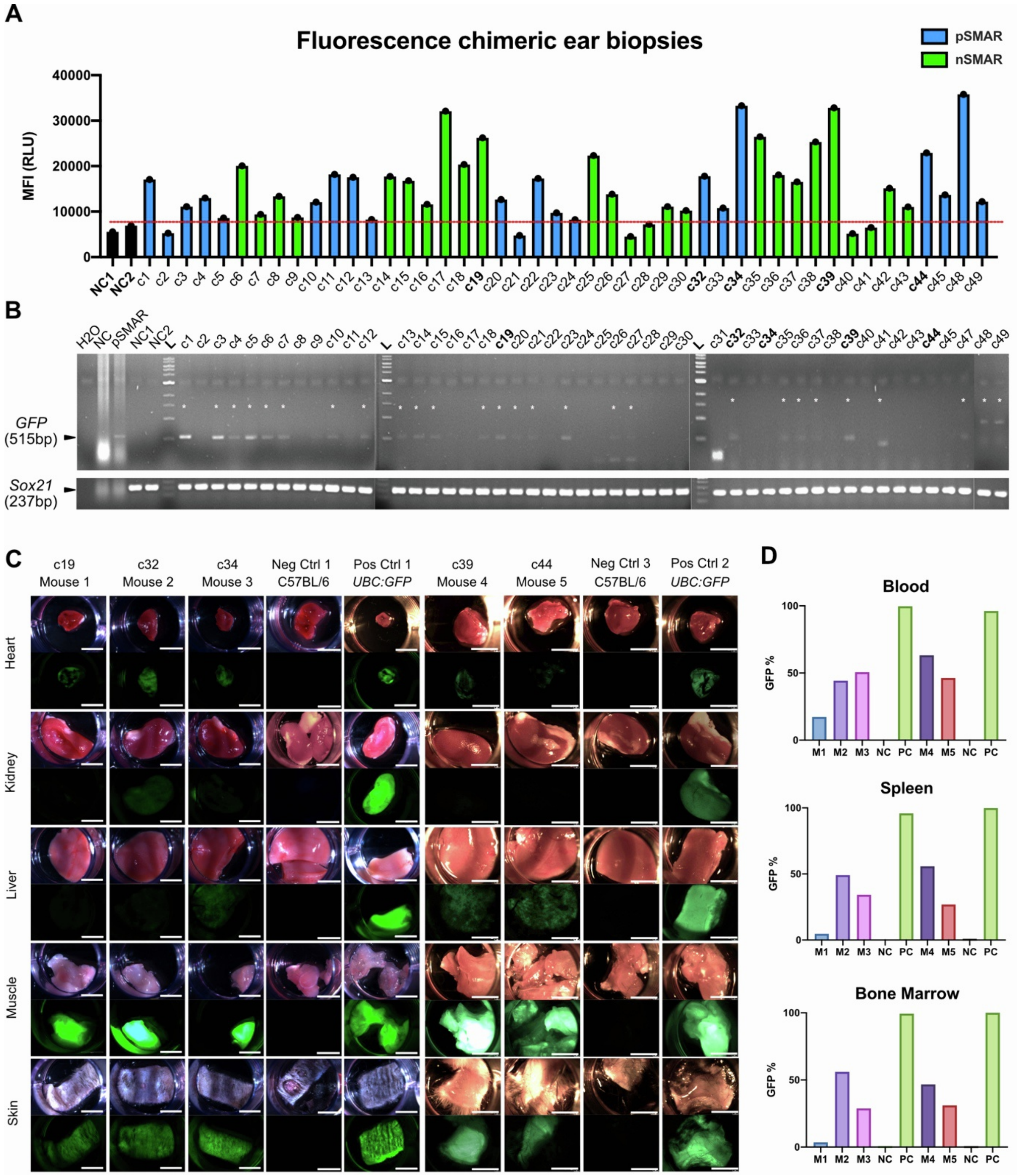
A
Fluorescence chimeric ear biopsies
pSMAR
nSMAR
MFI (RLU)
B
GFP (515bp)
Sox21 (237bp)
C
c19 Mouse 1
c32 Mouse 2
c34 Mouse 3
Neg Ctrl 1 C57BL/6
Pos Ctrl 1 UBC:GFP
c39 Mouse 4
c44 Mouse 5
Neg Ctrl 3 C57BL/6
Pos Ctrl 2 UBC:GFP
Heart
Kidney
Liver
Muscle
Skin
D
Blood
Spleen
Bone Marrow
GFP %

Figure S4: SMAR vectors survive in vivo differentiation and contribute to form chimeras (related to Table S3)

(A) Transgenic (GFP) expression measured from fluorescent microscopy images of ear biopsies of born chimeric pups (n=49) at the time of weaning, and C57BL/6N control mice (n=2). The mean fluorescence is expressed as RLU. The red line represents the threshold for autofluorescence background. Bolded animals were selected for further analysis

(B) Genotyping PCR from ear biopsies of chimeric mice (n=49) at the time of weaning, and C57BL/6N control mice (NC, n=2). Water (H_2O) was used as technical negative control. DNA from HEK293 wildtype or transfected with pSMAR vector (GFP- and GFP+ lysate) were used as biological controls. The transgene (GFP) amplicon corresponds to a 515bp band. An internal mammalian conserved Sox21 sequence (237bp) was used as internal control.

(C) Fluorescent images of chimeric organs derived from the three germ layers in the selected chimeras (c19, c32, c34, c39 and c44). The experiment was performed in 2 days. Constitutively expressing UBC:GFP mice (Jackson lab) were used as positive controls and a C57BL/6N mice as negative controls. (Leica M205FA, exposure 1s, amp gain 1,9x, digital exposure 4, Scale bars = 5mm).

(D) Flow Cytometry analysis of transgene expression in blood, bone marrow and spleen. A panel of surface markers was used to subgroup GFP+ blood cells into T cells (CD4, CD8a), B cells (B220) and Myeloid cells (CD11b). The pan-blood surface marker CD45 was used to gate GFP+ viable cells in bone marrow and spleen (The experiment was performed in 2 days with the respective 2 sets of controls)

Table S1

Table S 1: Downregulated and Upregulated genes upon delivery of pSMAR and nSMAR vectors

Table S1 is provided as a separate excel file

Table S2

Table S 2:Coat chimerism of pSMAR and nSMAR chimeras

mESC clone	coat color	% chimerism
pSMAR 71c22	Agouti/chinchilla/black	40% Chinchilla
pSMAR 71c22	Agouti/chinchilla/black	5% Chinchilla
pSMAR 71c22	Agouti/chinchilla/black	65% Chinchilla
pSMAR 71c22	Agouti/chinchilla/black	40% Chinchilla
pSMAR 71c22	Agouti/chinchilla/black	40% Chinchilla
pSMAR 71c22	Agouti/chinchilla/black	5% Chinchilla + 50% Agouti
pSMAR 71c22	Agouti/chinchilla/black	20% Chinchilla
pSMAR 71c22	Agouti/chinchilla/black	15% Chinchilla
pSMAR 71c22	Agouti/chinchilla/black	10% Chinchilla
pSMAR 71c22	Agouti/chinchilla/black	5% Chinchilla
pSMAR 71c22	Agouti/chinchilla/black	90% Agouti
pSMAR 71c22	Agouti/chinchilla/black	30% Agouti
pSMAR 71c22	Agouti/chinchilla/black	30% Agouti
pSMAR 71c22	Agouti/chinchilla/black	30% Chinchilla
pSMAR 71c22	Agouti/chinchilla/black	20% Chinchilla
pSMAR 71c22	Agouti/chinchilla/black	10% Chinchilla
pSMAR 71c22	Agouti/chinchilla/black	50% Agouti
nSMAR 85c17	Agouti/chinchilla/black	15% Chinchilla
nSMAR 85c17	Agouti/chinchilla/black	10% Chinchilla
nSMAR 85c17	Agouti/chinchilla/black	5% Chinchilla
nSMAR 85c17	Agouti/black	70% Agouti
nSMAR 85c17	Agouti/black	20% Agouti
nSMAR 85c17	Agouti/chinchilla/black	60% Chinchilla
nSMAR 85c17	Agouti/chinchilla/black	30% Chinchilla
nSMAR 85c17	Agouti/chinchilla/black	5% Chinchilla
nSMAR 85c17	Agouti/black	100% Agouti
nSMAR 85c17	Agouti/black	50% Agouti
nSMAR 85c17	Agouti/black	20% Agouti
nSMAR 85c17	Agouti/chinchilla/black	55% Chinchilla
nSMAR 85c17	Agouti/chinchilla/black	30% Chinchilla
nSMAR 85c17	Agouti/chinchilla/black	5% Chinchilla + 65% Agouti
nSMAR 85c17	Agouti/chinchilla/black	90% Chinchilla
nSMAR 85c17	Agouti/chinchilla/black	65% Chinchilla
nSMAR 85c17	Agouti/chinchilla/black	40% Chinchilla
nSMAR 85c17	Agouti/chinchilla/black	30% Chinchilla
nSMAR 85c17	Agouti/chinchilla/black	20% Chinchilla
nSMAR 85c17	Agouti/chinchilla	95% Chinchilla
nSMAR 85c17	Agouti/chinchilla	90% Chinchilla
nSMAR 85c17	Agouti/chinchilla/black	85% Chinchilla
nSMAR 85c17	Agouti/chinchilla/black	50% Chinchilla
nSMAR 85c17	Agouti/chinchilla/black	30% Chinchilla
nSMAR 85c17	Agouti/chinchilla/black	5% Chinchilla + 50% Agouti

Table S3

Table S 3: Transgenic expression in chimeric organs

Bolded mice were selected as examples to represent in the main figures and were analyzed for germline transmission. Transgenic expression of heart, kidney, liver, skeletal muscle, skin, testis and sperm, was assessed using fluorescence microscopy. Transgenic expression and quantification of GFP in spermatogonias, blood, spleen and bone marrow, was assessed using flow cytometry

Mouse ID	c19 Mouse 1	c32 Mouse 2	c34 Mouse 3	c39 Mouse 4	c44 Mouse 5	Neg Ctrl 1	Neg Ctrl 2	Neg Ctrl 3	Pos Ctrol 1	Pos Ctrol 2
Vector	**nSMAR**	**pSMAR**	**nSMAR**	**nSMAR**	**pSMAR**	**BL/6N**	**BL/6N**	**BL/6N**	**UBC:GFP**	**UBC:GFP**
Heart	yes	yes	yes	**yes**	**yes**	no	n/a	**no**	yes	**yes**
Kidney	no	yes	no	**no**	**no**	no	n/a	**no**	yes	**yes**
Liver	no	yes	yes	**yes**	**yes**	no	n/a	**no**	yes	**yes**
Muscle	yes	yes	yes	**yes**	**yes**	no	n/a	**no**	yes	**yes**
Skin	yes	yes	yes	**yes**	**yes**	no	n/a	**no**	yes	**yes**
Testis	n/a	n/a	n/a	**yes**	**yes**	n/a	n/a	**no**	n/a	**yes**
Sperm	n/a	n/a	n/a	**no**	**no**	n/a	n/a	**no**	n/a	**yes**
Spermatogonias	n/a	n/a	n/a	**8,72%**	**1,83%**	n/a	n/a	**0,22%**	n/a	**82,60%**
Blood	17,30%	44,30%	50,60%	**63,20%**	**46,30%**	0,04%	0,05%	**0,26%**	99,70%	**96,10%**
Bone Marrow	3,53%	56,00%	28,80%	**46,70%**	**31,00%**	0,09%	0,09%	**0,11%**	99,40%	**100,00%**
Spleen	4,74%	49,10%	34,20%	**55,80%**	**26,90%**	0,06%	0,07%	**0,40%**	95,90%	**99,90%**
Testis	n/a	n/a	n/a	**yes**	**yes**	n/a	n/a	**no**	n/a	**yes**
Sperm	n/a	n/a	n/a	**yes**	**yes**	n/a	n/a	**no**	n/a	**yes**

SUPPLEMENTAL METHODS

Table S 4: Reagents, Resources and Sequences

Table S4 is provided as a separate excel file

DNA sequencing

Sequencing of DNA plasmids was performed by GATC Biotech AG (Eurofins Genomics). For this, 5 µl of >100 ng/µl plasmid DNA was mixed with 5 µl of the appropriate primer at a final concentration of 5 µM.

Fluorescence microscopy

To image engineered SCs with SMAR vectors, GFP expression was imaged with a 10X or 20X objective in a Nikon Eclipse Ti/X-Cite 120Led microscope.
To detect the presence of SMAR vectors in the organs of chimeric mice, GFP was imaged in sections of heart, kidney, liver, skin, skeletal muscle and testis were with a motorized fluorescent stereomicroscope (Leica M205 FA. Exposure 1 sec, Amp gain 1,9x).

Immunofluorescence

Stem cell colonies or differentiating structures were washed twice in cold PBS, fixed with 100% methanol for seven min at -20˚C followed by a quick rinse in ice-cold acetone for 20 sec (note that this fixation method quenches endogenous GFP fluorescence). Then, the cells were permeabilized with 0.1% Tween20 in PBS (PBST) for five min at room temperature and washed three times with cold PBST. Blocking was done in PBS with 1% FCS, 0.5% BSA, 0.1%TritonX100 for 30 min at room temperature. The primary antibody was diluted in blocking solution and incubated at 4˚C over night. Thereafter, samples were washed three times with blocking solution and then incubated with the respective secondary Alexa-Fluor-conjugated antibodies (Donkey anti-Mouse 594 and Donkey anti-Rabbit 647,1:1000 Abcam) and 2 µg/ml DAPI (Sigma) as a nuclear counterstaining for 1h at room temperature and protected from the light. Finally, the stainings were imaged with a 20x objective in a Nikon Eclipse Ti/X-Cite120Led microscope.
To enable endogenous GFP imaging, the IF protocol was changed to fixation with 4% PFA (Himedia) and all steps were performed under protection from light. Furthermore, cells were permeabilized using PBST for five min and washed 3x for 5min with 0.1% Triton-X-100 in PBS. Blocking was performed with 3% BSA in PBST for 1 h at room temperature. Primary antibodies were diluted in this blocking buffer and incubated over night at 4˚C. The next day, cells were washed three times with PBS and incubated with the respective secondary antibody coupled to an Alexa-Fluor and DAPI for 1 h at room temperature before three further washings with PBS and imaging.
For pluripotency stainings, the cells were incubated with the following primary antibodies diluted in the respective blocking buffer. For **murine SCs:** rabbit polyclonal OCT4 (Abcam, 1:500), rabbit polyclonal NANOG (Abcam, 1:500), mouse monoclonal SSEA1 (Santa Cruz, 1:200), rabbit polyclonal SOX2 (Merck, 1:500, rabbit polyclonal KLF4 (Santa Cruz, 1:200)) and for **human SCs:** (mouse monoclonal TRA-160 (Santa Cruz, 1:100), goat polyclonal OCT-3/4 (Santa Cruz, 1:100), mouse monoclonal LIN28 (Santa Cruz, 1:50) rat monoclonal SSEA3 (Santa

Cruz, 1:100), mouse monoclonal SSEA4 (Santa Cruz, 1:100) and mouse monoclonal NANOG (Santa Cruz, 1:150) were used.
For differentiating structures from the **three germ layers**, the primary antibodies used were: mouse monoclonal β3-TUBULIN (Santa Cruz, 1:100) for ectoderm, mouse monoclonal αSMA [(Santa Cruz, 1:100) for methanol/acetone fixation or (Invitrogen, 1:200) for PFA fixation] for mesoderm and mouse monoclonal FOXA2 (Santa Cruz, 1:100) for endoderm.

Immunohistochemistry and HE stainings

Organs derived from the three germ layers from chimeric and transgenic pups, as well as *UBC:GFP* mice (Jackson lab) and C57BL/6J were fixed in 4% PFA in PBS for 24h, paraffin embedded and sectioned into 5 µm thick specimens and dried at 37˚C for 2h. Prior to staining, the tissue sections were deparaffinized in xylol (twice for 10 min), and rehydrated with consecutive five min washes in 100%, 100%, 96%, 80%, 70% ethanol and finally in distilled water. Routine Hematoxylin-Eosin (HE) staining was as previously described (Neufang et al., 2001).
For immunohistochemistry, the epitopes were exposed by damp heat-induced epitope retrieval by boiling the samples 15 min in a steam pot with citrate buffer pH 6.0, followed by a 30 min cooling period and a rinse with water. Then the sections were blocked with Avidin/Biotin (Avidin/Biotin blocking kit, SP-2001, Vector Laboratories) following the manufacturers' instructions. Then, the samples were incubated with the primary antibody rabbit anti-coGFP (Abcam, 1:500) diluted in Dako Real antibody diluent (Dako) for 30 min at room temperature followed by a rinse in PBST. The secondary antibody (Goat anti-rabbit from the Dako Peroxidase/AEC detection kit, Dako) was incubated for 20 min at room temperature. The endogenous peroxidase activity was blocked by incubation in Dako Real Peroxidase blocking solution (Dako) for five min. Then, the streptavidin-peroxidase HRP (Dako) was added and incubated for 20 min. Finally, the chromogen was added and the reaction was monitored under the microscope. The sections were counterstained for one min with hematoxylin, rinsed in tap water and mounted in Aquatex mounting media.

Microarray

RNA from hESC pellets was extracted using RNeasy Mini Kit (Qiagen), following the manufacturer's instructions. DNaseI treatment was performed before downstream applications (Ambion DNA-free Kit, Invitrogen), following the manufacturer's instructions. The array was performed in the DKFZ Genomic and Proteomic Core Facility using the IlluminaHuman12 chip and the normalization across the samples was performed there.
For the analysis, biological triplicates were used, and the gene expression was background corrected, quantile normalized, and $\log_2$ transformed using the Limma package from R (Ritchie et al., 2015; Shi et al., 2010). Linear modelling was performed and the Empirical Bayes method was used to assess differential expression (Phipson et al., 2016). *P* values were adjusted using the Benjamini-Hochberg adjustment method. Top differentially expressed genes between pairwise comparisons were determined using F-statistics and top differentially expressed genes within a pairwise comparison were determined using B-statistics. Median was used as a clustering method for hierarchical clustering as it gave the largest cophenetic correlation coefficient from the 8 methods tested. Volcano plots were designed using EnhancedVolcano from R (Blighe et al., 2019) with using an adjusted *P* value<0.05 ($-\log_{10}P$ of 1.3) and a FC>2 ($\log_2$FC of 1). Venn diagrams between each pairwise

comparison were adjusted with a P value<0.05 and FC> 2. Hierarchical clustering was performed using Euclidean as a distancing measure and Median as a clustering method. The average normalized expression from the top 100 differentially expressed genes, determined by F values, was plotted for each treatment. Hierarchical clustering heat maps were designed using ComplexHeatmap from R (Gu et al., 2016).

Animals care and use

Mice were kept in IVC cages under SPF conditions in the central animal facility at the German Cancer Research Center (DKFZ, Heidelberg). All animal experiments were performed following institutional and governmental regulations and were approved by the local authorities (Regierungspräsidium Karlsruhe, Germany). Mice of both sexes were used, and experimental mice were housed with 4-5 mice per greenline cage. All mice were immune-competent and healthy. Tail biopsies or ear punches were taken at the time of weaning and were used for genotyping. Necropsy was done under license DKFZ 345.

Derivation and culture of mES cells

The derivation of mESCs was done according to (Tesar, 2005) in the presence of 2i (**GSK3 inhibitor** CHIR99021, **MEK inhibitor** PD0325901)**.** ESC culture was done according to the protocols at www.eummcr.org
E14 129Ola mESCs were obtained from the EMBL, Heidelberg) and established in culture as described (Evans and Kaufman, 1981; Hooper et al., 1987)

Embryo collection

To obtain fertilized oocytes (zygotes), 5-8-week-old females were superovulated upon intraperitoneal hormone administration: 7 I.U. gonadotropin (PMSG, Pregnant Mares serum gonadotropin), 46-48h later 7 I.U. choriogonadotropin (hCG, human chorionic gonadotropin), each dissolved in 0.1 ml of physiological saline. At least one hour after the last hormone administration, the females were paired with males of the same strain. Females with vaginal plug (VP) were sacrificed the day after by cervical dislocation, their fallopian tubes were removed, and the eggs were rinsed from the ampulla.
To obtain blastocysts, VP-positive females were sacrificed by cervical dislocation 2,5 or 3.5 days after mating. The fallopian tubes and part of the uterus were removed and the morulae (E2.5) or blastocysts (E3.5) were isolated by flushing of the fallopian tube/uterus. Transgenic mESCs were injected into the blastocoel of E3.5 blastocysts.

Blastocyst injections

Blastocyst injections were performed under the License Number G-148/13 by the DKFZ Transgenic Service. 129Ola E14-1 Mouse Embryonic Stem Cells (mESCs) were electroporated *in vitro* as described below, either with pSMAR-CAG:GFP-2A-Puro-SMAR (pSMAR); or nSMAR-CAG:GFP-2A-Puro-SMAR (nSMAR). Electroporated cells were cultured as described and between 6 - 12 stem cells were injected into either *morulae* (E2.5) of CD1 embryos or blastocysts (E3.5) of C57BL/6N x B6D2F1 embryos. On the day after injection (for morula-injection or on the day of injection (for blastocysts), the embryos were transferred into pseudo-pregnant females (CD1) that were previously mated with sterile males (CD1). The

embryos were brought to term, and the pups were checked for coat chimerism as well as for GFP expression.

Pronuclear injections

Pronuclear injections were performed under the License Number: G-97/12 by the DKFZ Transgenic Service.
Before injection, 100 µl of plasmid DNA (vector 71) at a concentration 1000 ng/µl were filtered through a Millipore Millex-GV 0.22 µm by using a disposable 1ml syringe and with an air-bubble to fill up the rest of the volume. Then, 50 µl of filtered DNA was placed on top of a floating Millipore membrane VMWPO2500 (0.025 µm pore size) and desalted via dialysis in 50 - 100 ml of Dialysis Buffer (100 ml Ampuwa ddH_2O, 10 mM Tris and 0.1 mM EDTA) for 3 hours at 4˚C to avoid evaporation and DNA loss. After 3h, the DNA drop was carefully recovered, and both quality and quantity of DNA were assessed via spectrophotometry (Nanodrop) and Agarose Gel electrophoresis. DNA recoveries ranging from 70 - 80% were achieved. The DNA solution was stored at -20˚C or used for downstream applications.
The day of injection, 1 - 2 picoliters of plasmid DNA at a concentration of 1 – 3 ng/µl were injected into the pronucleus of E0.5 murine C57BL/6N zygotes. After injection, the zygotes were transferred into pseudo-pregnant females (CD1) that were previously mated with sterile males (CD1). Some embryos (n=12) were kept in culture in KSOM (Millipore) to check for transgene toxicity and developmental abnormalities. The rest of the embryos were brought to term and the pups were checked for GFP expression.

Transgenic sperm collection

Sperm collection was performed by the DKFZ Cryopreservation Service.
Two BL/6J males were euthanized with CO_2 inhalation and cervical dislocation. After an abdominal incision and a peritoneal cut, the testes and *cauda epididymis* were exposed, cut, and separately placed in PBS. With the aid of a stereomicroscope and tweezers, the *cauda epididymis* was cut, and the sperm was released into the buffer. Modified protocol from (Varisli et al., 2013).
For imaging, 10 µl of sperm in PBS were placed on a slide and covered with a coverslip. Then recordings of motility or fluorescence images were taken using a Nikon Ti microscope and a 20x objective. When the sperm could not be freshly imaged, it could be kept alive and motile at room temperature for up to 2 hours. The remaining sperm was pelleted and frozen for total DNA extraction and downstream applications.

Human cells

Normal human dermal fibroblasts (NHDFs, 3-year old male) were cultured on gelatin-coated dishes (0.1% gelatin in distilled water) and grown in DMEM (4500 mg/L glucose, L-glutamine, sodium bicarbonate, without sodium pyruvate), supplemented with 10% FBS (Gibco), 1% Pen/Strep (Gibco) and 1% NEAA (Gibco). The cells were cultured in a humidified atmosphere at 37˚C and 5% CO_2 and passaged when reaching >80% confluence. HDFs were purchased from PromoCell (C-12300), where authentication has been performed. **Urine Cells (UCs)** were obtained from healthy donors (ethical approval from the ethics committee of the medical faculty of the University of Heidelberg, study number S-550/2019) as described before (Mulder et al., 2020).

hESCs and hiPSCs, were grown on mitotically inactivated MEFs (passage 4), using Mitomycin-C at a final concentration of 10 ug/ml in complete DMEM, and 0.1% gelatin-coated plates. They were cultured in hESC media containing DMEM/F-12 + Glutamax (Gibco) supplemented with 20% KnockOut Serum Replacement (Gibco), 1% Pen/Strep (Gibco), 1% NEAA (Gibco) and 0.1mM β-mercaptoethanol (Gibco). A final concentration of 10-30 ng/µl of FGF2 (Peprotech) was added freshly. The media was replaced every second day and the cells were passaged by manual excision once a week. hiPSCs were derived from HDFs (3-year old male) or urine cells (male and female) by electroporation of pCXLE EBNA reprogramming vectors using feeder dependent or feeder-free reprogramming. hESCs were male.
For genetic modification at the SC stage, hiPSCs were transferred to a feeder-free culture system using iMatrix Laminin-511 (Amsbio) as coating reagent and Stemfit Basic02 media (Ajinomoto) supplemented with 10-30 ng/ul FGF2 (Peprotech). There, cells were passaged in clumps using ReLesR (Stem Cell Technologies) or as single cells using TrypLE (Gibco) or ProStem Accutase (Gibco) and media was changed three times per week.

Murine cells

MEFs and **lung fibroblasts** were cultured on gelatin-coated dishes (0.1% gelatin in distilled water) and grown in DMEM (with 4500 mg/L glucose, L-glutamine, and sodium bicarbonate, without sodium pyruvate), supplemented with 10% FBS (Gibco) and 1% Pen/Strep (Gibco). The cells were cultured in a humidified atmosphere at 37˚C and 5% CO_2. The cells were passaged on demand. CF1-MEFs (male, passage 3) were purchased from Merck. Primary lung fibroblasts were obtained from female C57BL/6N mice (Charles River, Sulzfeld, Germany) and generated as described before (Willemsen et al., 2017).
Immortalized Mouse Embryonic Fibroblasts (iMEFs) were used as feeder layers for co-culture of murine stem cells and reprogramming of human fibroblast. The cells were cultured as described above and grown until 90% confluency. Then the cells were washed, trypsinized and mitotically inactivated upon exposure to 60 Gy of γ-irradiation (Gamma cell 1000).
Mitotically inactivated MEFs (reference, Passage 4) using Mitomycin-C at a final concentration of 10 ug/ml in complete DMEM were used as feeder cells and co-cultured with human stem cells.
E14 129Ola mESC and miPSC were grown on feeder layers (on 0.1% gelatin-coated plates) and in KnockOut DMEM (Gibco) supplemented with 15% FCS superior (Biochrom), 1% Pen/Strep (Gibco), 1% NEAA (Gibco), 1% L-glutamine (Gibco), 0,1 mM β-mercaptoethanol (Gibco) and 1000 U/ml of Leukemia Inhibitory Factor (Merck). The differentiation inhibitors CHIR99021 and PD0325901 (Sigma) were added fresh to the media before use at a final concentration of 3 µM and 1 µM, respectively. The cells were cultured in a humidified atmosphere at 37˚C and 5% CO_2. The media was changed every second day and the cells were passaged twice a week. In order to separate mESCs from feeder layers, an intermediate differential sedimentation step was performed for 20 minutes at 37˚C and 5% CO_2 in IMDM media (Gibco) supplemented with 10% FCS (Gibco) and 1% Pen/Strep (Gibco). If needed, the cells were adapted into a feeder-free culturing system. mESC (E14 129Ola strain, male), miPSC were derived from CF1-MEF (Merck, male) or lung fibroblasts (female) by transduction with pWPI-4in1 lentiviral particles.

Lentiviral reprogramming

MEFs at a low passage (p3 - 5) were seeded (25.000 - 50.000 cells) per well of gelatinized 24 well plate. Transduction was performed in three rounds. The next morning (day 1), the media was aspirated, and the cells were infected with 200µl of viral particles suspended in DMEM media. The infection was repeated on the evening of the same day (day 1) and the following morning (day 2). Finally, the viral supernatant was aspirated, and without washing steps, the cells were fed with standard DMEM supplemented with 10% FCS and 1% Pen/Strep.

DNA Vector reprogramming

HDFs were dissociated with trypsin-EDTA (sigma) and 500.000 cells were transfected with either 2 µg of each episomal EBNA reprogramming plasmid (pCXLE-hUL, pCXLE-hSK, pCXLE-hOCT3/4-shp53-F, and pCXWB-EBNA1; Addgene) and if indicated, co-transfected together with labeling GFP vectors (pSMAR or nSMAR). Alternatively, HDFs were transfected with 2-3µg of SMAR reprogramming vectors (pPOP or nPOP) and co-transfected with 2 µg of the GFP-SMAR labeling vector pSMARt-shP53. The plasmid DNA was diluted in electroporation buffer (Lonza) and the cells were electroporated using the program P-022 and the Amaxa II Nucleofector device (Lonza) following the manufacturer's instructions. Following transfection, the cells were seeded on a six-well plate containing DMEM supplemented with 10% FCS (Gibco), 1% NEAA (Gibco) without PenStrep (Gibco), to allow the cells to recover. The PenStrep was added 24h after transfection and the media was changed every second day. At 8 days after transfection, 30.000 cells were seeded per well in triplicates in a six-well plate coated with 0.1% gelatin and containing feeder cells in hiPSC media containing DMEM/F12 with Glutamax supplement (Gibco), 20% Knockout Serum replacement (Gibco), 0.1 mM β-mercaptoethanol (Gibco), 1% Pen/Strep (Gibco), 1% NEAA (Gibco) and 10 ng/ml FGF2 (Peprotech). The hiPSC media was replaced every second day until hiPSC colonies emerged. Every experiment was repeated at least twice.
UCs were reprogrammed by transfection of episomal EBNA vectors as described elsewhere (Mulder et al., 2020) and transferred to feeder-free hiPSC conditions after 4 days and kept in these conditions.

Random differentiation

Murine random differentiation was achieved through the formation of EBs. For that, mESCs were passaged as described above and diluted in EB medium, containing KnockOut DMEM (Gibco), 15% KnockOut Serum Replacement (Gibco), 1% L-Glutamine (Gibco), 1% NEAA (Gibco), 1% Pen/Strep (Gibco) and 50 µg/ml Ascorbic acid (Sigma), devoid of LIF and the 2i inhibitors, to a density of $3x10^4$ cells/ml (or 600 cells per 20 µl). With the help of a multichannel pipette, 80-100 drops of 20 µl were placed on the lid of a bacterial petri dish, and the dish was filled with PBS to preserve the humidity. The drops were incubated at 37˚C and 5%CO_2 for 3 days. During this time, the cells collapsed in the bottom of the drop and formed undifferentiated aggregates. After 3 days, the drops from one lid were harvested and pooled with 4 ml of EB media, and the aggregates were cultured in suspension in EB media for additional 3 days. Then, the aggregates were transferred into a 15 ml conical tube with EB media and were sedimented at room temperature for 10 minutes. They were resuspended in fresh EB media and 1-2 aggregates were transferred per well of a gelatin-coated µ-plate 96 well (iBidi). The plate was incubated for 1-2 weeks and the EB media was

replaced every second day. The embryonic Bodies were checked regularly for differentiation and when obvious differentiated structures formed (*e.g.:* beating cardiomyocytes, neurons…), they were fixed and stained with antibodies for markers of the three germ layers (β3-TUBULIN, αSMA and FOXA2).
Human tri-germ layer differentiation was achieved using the StemMACS trilineage differentiation kit (Miiltenyi Biotec) following the manufacturer's recommendations. Briefly, feeder-free maintained hiPSCs were detached as single cells using StemPro Accutase (Gibco) and plated in the described respective density in stem cell media containing 10 µM ROCK inhibitor. The cells were cultivated for 7 days with the respective germ layer induction media and successful induction was verified via immunofluorescence staining. For quantification of GFP expression after 7 days of differentiation, GFP+ population was determined in single, alive cells using fluorescence cytometry and compared to the respective hiPSC lines maintained without differentiation.

Hematopoietic differentiation

mESCs were grown to confluency, washed, trypsinized and separated from feeder cells. Then, 75.000 cells were counted and plated in Ultra low attachment T25 flasks (Corning) containing EB-HSC differentiation medium (IMDM, 15% EB FCS (PAA Laboratories), 1% Pen/Strep (Gibco), 1% L-Glutamine (Gibco), 50 µg/ml Ascorbic acid, 4.5 mM monothioglycerol (Sigma) and 200 µg/ml Holotransferin (Sigma)), and were incubated for 2.5 days (60h) under hypoxic conditions (5% CO_2 and 5% O_2). After 60h, 5 ng/ml of each cytokine BMP-4 (R&D), Activin A (R&D), VEGF (R&D) and FGF2 (R&D) were added to the media and the cells were further incubated for 60h in hypoxia. At day 5, the EB were allowed to settle at the bottom of the flask. Then, the majority of the media containing cytokines was removed and centrifuged to pellet cell debris. Fresh EB media without cytokines was added to the embryonic bodies, and the conditioned media was added back to the flask, which was then incubated for another 24h in hypoxic conditions.
At day 6, the EBs were collected, washed and dissociated in 250 µl Enyzme mix containing 10 mg/ml Collagenase, 0.02 g/ml Hyaluronidase and 80 Units/ml DNaseI in 1 ml of PBS and incubated at 37˚C for 20 min with occasional swirling of the tube. Another 8 ml of enzyme-free dissociation media were added to the cells, which were then incubated for five minutes at room temperature. Finally, the cells were mechanically dissociated and washed with PBS, collected by centrifugation (1500 rpm for five min at 4˚C) and used for downstream analysis such as FACS.
The hematopoietic differentiation panel of antibodies used, included: CD41 (1:300), CD144/VE-Cadherin (1:400), CD117/c-Kit (1:2000) and 7-AAD (1:200) as life/death staining.

Bacterial strains

Bacterial competent cells DH5α (Life Technologies), DH10β (Life Technologies), Stbl3 (Life Technologies), and Stellar competent cells (Clontech), were grown in Luria-Bertani (LB) media or LB-agar plates with the corresponding antibiotic (Ampicillin 100 µg/ml; Carbenicillin 50 µg/ml; Kanamycin 30 µg/ml) at 37˚C.

Viral vectors

Lentiviral particles were generated in the laboratory of Dr. Marco Binder (DKFZ, Germany). Briefly, HEK293T cells were infected with viral vectors derived from the plasmid pWPI-BLR. The lentiviral particles were produced as described elsewhere (Koutsoudakis et al., 2006) by calcium phosphate transfection of the three individual plasmids at a 3:1:3 ratio: (i) pCMV-

ΔR8.91, coding for HIV Gag-Pol; (ii) pMD.2G, encoding the VSV-G glycoprotein; and (iii) the lentiviral vector pRRL.PPT.SF.hOKSM-IRES-dTOM or empty pWPI-Puro. (pCMV-ΔR8.91 and pMD.2G) were kind gifts from Didier Trono, Lausanne (Zufferey et al., 1997). Finally, the supernatants of HEK293T cells were collected, filtered, and frozen for further use, without viral particle titration.

Genotyping

DNA from tail biopsies or ear punches was extracted with the Phire Tissue Direct PCR Master Mix (Thermo Fischer) according to the manufacturer's instructions, in a total volume of 20 µl per reaction. Degenerate primers amplifying a non-coding mammalian genomic DNA region of *SOX21* were provided with the kit and used as internal controls (Primers G11-12). GFP primers for amplification of *coGFP* (515bp) or *eGFP* (237bp) were designed using Primer3 and validated *in silico (*Primers G7 - G8 for *coGFP* and G9 -G10 for *eGFP).* The PCR reactions for GFP and the internal controls were performed separately to get better amplification of GFP bands.

Alkaline Phosphatase (AP) staining

AP stainings were performed in black µ-Plate 96 Well (Ibidi) coated with gelatin or iMatrix Laminin-511 or in regular coated 24-well plates. The staining was performed using Alkaline Phosphatase Staining Kit II (Stemgent) as of manufacturer's instructions. The images were taken with a Keyence microscope (Keyence).

FACS

Flow Cytometry analysis was performed on a LSR Fortessa (BD Biosciences) and the data acquisition was done in a BD CellQuest Pro^{TM} or BD FACSDiva^{TM} software (BD Biosciences). Briefly, cells were washed, detached using trypsin or accutase and resuspended in 200ul PBS. Propidium Iodide (Life technologies) or 7-AAD (Invitrogen) were used as life-death discrimination and added to the cells shortly before FACS (PI, 1:1000; 7-AAD, 1:200). Data analysis was done with the software FlowJo. For transfected cells, no staining was needed as endogenous GFP expression could be detected. EB staining: CD41-PE-Cy7 (eBioscience, 1:300), CD144/VE-Cadherin-PE (BD Pharmigen, 1:400), CD117/c-Kit-Alexa 780 (eBioscience, 1:2000) and 7-AAD (1:200) as life/death staining. Blood Staining: CD45.1-APC-Cy7 (eBioscience, 1:300), CD11b - APC (eBioscience, 1:2000, B220-APC (eBioscience, 1:500), CD4-PE-Cy7 (eBioscience, 1:2000), and CD8a-PE-Cy7 (eBioscience, 1:3000), antibodies were prepared in 2% FCS/PBS. 7-AAD/2% FCS/PBS (5µl/ml, 1:200 dilution). Spleen staining: spleen was smashed through a filter (40 µm EASYstrainer, Greiner bio-one) and collected in 2% FCS/PBS. The cells were then stained with CD45-PB (eBioscience, 1:1000). Bone marrow: bones were cleaned from muscle, and the bone marrow was flushed out in 2 ml 2% FCS/PBS with the help of a syringe. The bone marrow cells were then stained with CD45-PB (eBioscience, 1:1000).

Plasmid rescue

500 - 1000ng of genomic DNA extracted from stable cells were used for transformation into DH10β *E.coli* competent cells (Life Technologies) in a Bio-Rad Gene Pulser using a standard pulse for bacteria. Transformed colonies were selected on agar plates containing the appropriate antibiotic. DNA was isolated from individual resistant clones, subjected to restriction analysis with the appropriate enzymes, and analyzed by gel electrophoresis on 1% agarose gels.

Southern Blot

Genomic DNA was extracted from cells using the DNeasy blood and tissue kit (Qiagen) following the manufacturers' instructions and was digested with a DNA vector single-cutter (usually *BamHI or AgeI*) for at least 4h at 37˚C. Additionally, 2 – 5 ng of parental plasmids were digested as positive controls. The digested genomic and plasmid DNA fragments were separated on a 0.8% agarose gel overnight and transferred into a Hybond-XL nylon membrane (Amersham Biosciences). DNA probes used for Southern blot analysis were radioactively labeled with [32P]dATP (3000 Ci/mmol) or [32P]dCTP (3000 Ci/mmol) using Prime-it II Random Primer Labeling Kit (Agilent) and following the manufacturer's instructions. 100 µl of radioactively labeled DNA probe was denatured, diluted in 1 ml of Church buffer and hybridized overnight at 65˚C.

Western Blot

Between $5x10^5$ and $1x10^6$ cells were lysed in 40 µl RIPA lysis buffer containing 2% SDS, 10 mM Tris pH 7.5 and 0.1 mg/ml protease inhibitor (Roche) and centrifuged at 14000 g for 30 min at 4˚ C. The protein concentration of the cell lysate was determined using the Pierce™ BCA Protein Assay Kit (Thermo Fischer) and equal amounts (30 µg) were separated on a 4-12% SDS-PAGE gel (Bio-Rad) with a protein marker PageRuler Plus Prestained Protein Ladder (Fermentas). Following separation, total protein was transferred into PVDF iBlot 2 Transfer Stacks (Invitrogen) using an iBlot2 device (Invitrogen). The membrane was blocked in 5% non-fat milk in TBS-Tween20, and incubated with primary antibodies at the appropriate dilution, overnight at 4˚C. For detection, secondary antibodies conjugated to HRP were incubated with the membrane at the appropriate dilution for 1h at room temperature. The membrane was developed using SignalFire™ ECL Reagent (Cell Signaling Technology) by enhanced chemiluminescent detection system by FusionSL Vilber Lourmat system.
Pluripotency Western analysis was performed using: primary rabbit polyclonal KLF4 antibody (Santa Cruz, 1:500 dilution), goat polyclonal OCT3/4 (Santa Cruz, 1:500), rabbit polyclonal SOX2 (Merck, 1:1000), mouse monoclonal C-MYC (Santa Cruz, 1:150); mouse monoclonal LIN28 (Santa Cruz, 1:200), mouse monoclonal α-TUBULIN (Thermo Fischer, 1:10.000) or mouse monoclonal GAPDH (Santa Cruz, 1:500) as loading controls; and the secondary antibodies anti-mouse-HRP, anti-rabbit-HRP and anti-goat-HRP (Life Technolgies,1:10.000). For FancA western blots: the primary antibody rabbit polyclonal FANCA (Merck, 1:5000), mouse monoclonal GAPDH (Santa Cruz, 1:500) and the secondary antibodies anti-mouse-HRP, anti-rabbit-HRP and anti-goat-HRP (Life Technolgies, 1:10.000).

qRT-PCR

For qRT-PCR, approximately half a million cells were harvested and lysed in 500ul trizol. RNA was isolated as of manufacturers recommendation by addition of 100ul chloroform, separation of aqueous phase and precipitation using isopropanol. RNA was digested with DNase I using the DNA-free DNA removal kit (Invitrogen). Subsequent cDNA synthesis was performed using the RevertAid H minus first-strand cDNA synthesis kit (Invitrogen) and a mixture of oligo dT and random primers. For qRT-PCR, cDNA was diluted to 5ng/ul and 2ul was combined with a 23ul master mix composed of 12.5ul primaQuant cybr blue 2x qPCR sybrgreen master mix (Steinbrenner, SL-9902B), 8.5ul water, and 1ul of forward and reverse primer. (5uM) The reaction was run on a LightCycler96 instrument (Roche).
